# Decomposing neuroanatomical heterogeneity in depression: insights from an ENIGMA major depressive disorder working group study in 5146 individuals

**DOI:** 10.1038/s41398-026-04189-x

**Published:** 2026-06-23

**Authors:** Lukas Sempach, Sarah Ulrich, Stéphanie E. E. C. Bauduin, Jochen Bauer, Francesco Benedetti, Klaus Berger, Bianca Besteher, Robin Bülow, Colm G. Connolly, Emmanuelle Corruble, Baptiste Couvy-Duchesne, Kathryn R. Cullen, Udo Dannlowski, Christopher G. Davey, Danai Dima, Annemiek Dols, Jennifer W. Evans, Cynthia Fu, Paola Fuentes-Claramonte, Ali Saffet Gonul, Ian H. Gotlib, Roberto Goya-Maldonado, Hans J. Grabe, Nynke A. Groenewold, Dominik Grotegerd, Oliver Gruber, Tim Hahn, J. Paul Hamilton, Laura K. M. Han, Ben J. Harrison, Sean N. Hatton, Marco Hermesdorf, Ian B. Hickie, Tiffany C. Ho, Julia M. Hubbert, Neda Jahanshad, Hamidreza Jamalabadi, Alec J. Jamieson, Christoph Jurischka, Antonia Jüllig, Toshiharu Kamishikiryo, Tilo Kircher, Sheri-Michelle Koopowitz, Bernd Krämer, Anna Kraus, Judith Krieger, Axel Krug, Jim Lagopoulos, Meng Li, Andrew McIntosh, Susanne Meinert, Elisa M. T. Melloni, Benson Mwangi, Igor Nenadic, Amar Ojha, Go Okada, Mardien L. Oudega, Brenda W. J. H. Penninx, Sara Poletti, Edith Pomarol-Clotet, Maria J. Portella, Liesbeth Reneman, Elena Rodriguez-Cano, Matthew Sacchet, Raymond Salvador, Anouk Schrantee, Hotaka Shinzato, Kang Sim, Theresa M. Slump, Jair C. Soares, Dan J. Stein, Frederike Stein, Lea Teutenberg, Florian Thomas-Odenthal, Sophia I. Thomopoulos, Nic J. A. van der Wee, Steven J. A. van der Werff, Henry Völzke, Martin Walter, Heather C. Whalley, Sarah Whittle, Katharina Wittfeld, Mon-Ju Wu, Tony T. Yang, Carlos Zarate, Giovana B. Zunta-Soares, Paul M. Thompson, Dick J. Veltman, Elena Pozzi, Lianne Schmaal, André Schmidt

**Affiliations:** 1 Department of Clinical Research (DKF), University of Basel, Basel, Switzerland; 2 Center for Affective, Stress and Sleep Disorders, University Psychiatric Clinics (UPK) Basel, Basel, Switzerland; 3 Experimental Cognitive and Clinical Affective Neuroscience (ECAN) Laboratory, Department of Clinical Research (DKF), University of Basel, Basel, Switzerland; 4 Department of Psychiatry, Leiden University Medical Center, Leiden, the Netherlands; 5 Department of Radiology, University of Münster, Münster, Germany; 6 Division of Neuroscience, IRCCS Ospedale San Raffaele, Milano, Italy; 7 University Vita-Salute San Raffaele, Milano, Italy; 8 Institute of Epidemiology and Social Medicine, University of Münster, Münster, Germany; 9 Department of Psychiatry and Psychotherapy, Jena University Hospital, Jena, Germany; 10 Institute of Diagnostic Radiology and Neuroradiology, University Medicine Greifswald, Greifswald, Germany; 11 Department of Biomedical Science, Florida State University, Tallahassee, FL USA; 12 Service Hospitalo-Universitaire de Psychiatrie de Bicêtre, Mood Center Paris Saclay, Assistance Publique-Hôpitaux de Paris, Hôpitaux Universitaires Paris-Saclay, Hôpital de Bicêtre, Le Kremlin Bicêtre, Paris, France; 13 MOODS Team, INSERM 1018, CESP (Centre de Recherche en Epidémiologie et Santé des Populations), Université Paris-Saclay, Faculté de Médecine Paris-Saclay, Le Kremlin Bicêtre, Paris, France; 14 Institute for Molecular Bioscience, the University of Queensland, St Lucia, QLD Australia; 15 Sorbonne University, Paris Brain Institute - ICM, CNRS, Inria, Inserm, AP-HP, Hôpital de la Pitié Salpêtrière, Paris, France; 16 Department of Psychiatry and Behavioral Sciences, University of Minnesota, Minneapolis, MN USA; 17 Institute for Translational Psychiatry, University of Münster, Münster, Germany; 18 Department of Psychiatry, The University of Melbourne, Melbourne, VIC Australia; 19 Department of Psychology, School of Health and Psychological Sciences, City St George’s, University of London, London, UK; 20 Department of Neuroimaging, Institute of Psychiatry, Psychology and Neuroscience, King’s College London, London, UK; 21 Department of Psychiatry, UMC Utrecht Brain Center, University Medical Center Utrecht, Utrecht University, Utrecht, the Netherlands; 22 Amsterdam UMC, Vrije Universiteit Amsterdam, Department of Psychiatry, Amsterdam Neuroscience, Amsterdam, the Netherlands; 23 Experimental Therapeutics and Pathophysiology Branch, National Institute of Mental Health, National Institutes of Health, Bethesda, MD USA; 24 Centre for Affective Disorders, Department of Psychological Medicine, School of Academic Psychiatry, Institute of Psychiatry, Psychology and Neuroscience, King’s College London, London, UK; 25 Department of Psychology, University of East London, London, UK; 26 FIDMAG Germanes Hospitalaries Research Foundation, Barcelona, Spain; 27 CIBERSAM, ISCIII, Madrid, Spain; 28 Department of Psychiatry, Ege University, Izmir, Turkey; 29 Department of Psychology, Stanford University, Stanford, CA USA; 30 Laboratory of Systems Neuroscience and Imaging in Psychiatry (SNIP-Lab), Department of Psychiatry and Psychotherapy, University Medical Center Göttingen, Göttingen, Germany; 31 Department of Psychiatry and Psychotherapy, University Medicine Greifswald, Greifswald, Germany; 32 Department of Psychiatry and Mental Health, Neuroscience Institute, University of Cape Town, Cape Town, South Africa; 33 Section for Experimental Psychopathology and Neuroimaging, Department of General Psychiatry, Heidelberg University, Heidelberg, Germany; 34 Department of Biological and Medical Psychology, University of Bergen, Bergen, Norway; 35 Centre for Youth Mental Health, The University of Melbourne, Parkville, Australia; 36 Brain and Mind Center, University of Sydney, Sydney, NSW Australia; 37 Department of Psychology, University of California, Los Angeles, Los Angeles, CA USA; 38 Interdepartmental Graduate Program in Neurosciences, University of California, Los Angeles, Los Angeles, CA USA; 39 Mark and Mary Stevens Neuroimaging and Informatics Institute, Keck School of Medicine of USC, University of Southern California, Marina del Rey, CA USA; 40 Department of Psychiatry, University of Marburg, Marburg, Germany; 41 Department of Psychiatry and Neurosciences, Graduate School of Biomedical and Health Sciences, Hiroshima University, Hiroshima, Japan; 42 Department of Psychiatry and Psychotherapy, University Hospital Bonn, Bonn, Germany; 43 Thompson Institute, University of the Sunshine Coast, Sippy Downs, QLD Australia; 44 Centre for Clinical Brain Sciences, University of Edinburgh, Edinburgh, UK; 45 Institute for Translational Neuroscience, University of Münster, Münster, Germany; 46 School of Behavioral Health Sciences, The University of Texas Health Science Center at Houston, Houston, TX USA; 47 Center of Excellence on Mood Disorders, Louis A. Faillace, MD, Department of Psychiatry and Behavioral Sciences, McGovern Medical School, The University of Texas Health Science Center at Houston, Houston, TX USA; 48 Center for Neuroscience, University of Pittsburgh, Pittsburgh, PA USA; 49 Center for the Neural Basis of Cognition, University of Pittsburgh, Pittsburgh, PA USA; 50 GGZinGeest, Specialized Mental Health Care, Amsterdam, the Netherlands; 51 Amsterdam Neuroscience, Mood, Anxiety, Psychosis, Sleep & Stress program, Amsterdam, the Netherlands; 52 Institut de Recerca Sant Pau (IR Sant Pau), Barcelona, Spain; 53 Universitat Autònoma de Barcelona, Bellaterra, Spain; 54 Department of Radiology and Nuclear Medicine, Amsterdam UMC, University of Amsterdam, Amsterdam, the Netherlands; 55 Consorci Sanitari del Maresme, Barcelona, Spain; 56 Meditation Research Program, Department of Psychiatry, Massachusetts General Hospital, Harvard Medical School, Boston, MA USA; 57 Department of Neuropsychiatry, Graduate School of Medicine, University of the Ryukyus, Okinawa, Japan; 58 West Region, Institute of Mental Health, Singapore, Singapore; 59 Yong Loo Lin School of Medicine, National University of Singapore, Singapore, Singapore; 60 Lee Kong Chian School of Medicine, Nanyang Technological University, Singapore, Singapore; 61 SAMRC Unit on Risk and Resilience in Mental Disorders, Department of Psychiatry and Mental Health, Neuroscience Institute, University of Cape Town, Cape Town, South Africa; 62 Imaging Genetics Center, Stevens Institute for Neuroimaging and Informatics, Keck School of Medicine, University of Southern California, Marina del Rey, CA USA; 63 Leiden Institute for Brain and Cognition, Leiden University Medical Center, Leiden, the Netherlands; 64 Institute for Community Medicine, University Medicine Greifswald, Greifswald, Germany; 65 German Center for Mental Health (DZPG), Halle-Jena-Magdeburg, Germany; 66 Orygen, Parkville, Australia; 67 Department of Psychiatry and Behavioral Sciences, University of California San Francisco, San Francisco, CA USA; 68 Division of Child and Adolescent Psychiatry, University of California San Francisco, San Francisco, CA USA; 69 Weill Institute for Neurosciences, University of California San Francisco, San Francisco, CA USA; 70 National Institute of Mental Health, National Institutes of Health, Bethesda, MD USA

**Keywords:** Depression, Neuroscience, Biomarkers

## Abstract

The clinical and biological heterogeneity of major depressive disorder (MDD) may reflect the aggregation of different conditions with distinct pathologies under a single diagnostic label. Neuroanatomical heterogeneity in MDD was examined using a harmonized, age- and sex-matched sample from the ENIGMA MDD consortium (N = 5146; age range: 9–82 years; 64% female). Analyses of global neurostrucutral variability revealed greater cortical thickness heterogeneity in MDD compared with healthy controls (Cohen’s d = –0.26). Regionally, increased variability in cortical thickness was most prominent in the cingulate (+6.1 to +6.6% more variation in MDD) and insular (+5.8%) cortices, as well as in the frontal (+5.7 to +6.8%) and temporal (+6.1 to +6.8%) lobes. Heterogeneity in cortical thickness was more pronounced among patients using antidepressant medication (Cohen’s d = –0.39). Patient-specific analyses further showed that individuals with markedly increased cortical thickness variability (<5^th^ percentile relative to the normative range) exhibited greater depressive symptom severity than those within the normative range (5^th^–95^th^ percentile; Cohen’s d = 0.19–0.36). Overall, the results indicate that neuroanatomical heterogeneity in MDD is primarily expressed in cortical thickness, offering refined insights into the neurobiological complexity of structural alterations associated with depression. These findings could guide future stratification efforts examining whether regionally confined changes in cortical thickness within areas of pronounced variability reflect clinically meaningful patient subgroups.

## Introduction

Major Depressive Disorder (MDD) is the leading mental health contributor to the global burden of disease [1]. Clinically, MDD is a polythetic disorder characterized by substantial symptom heterogeneity, with a pattern of few prevalent, prototypical symptom combinations and numerous combinations with low probabilities of occurrence [2]. Only about 2% of patients with MDD share a fully overlapping symptom profile, while 14% present with unique profiles [3]. This heterogeneity extends to treatment outcomes: while initial antidepressant (AD) therapy achieves remission in approximately 40% of patients, about 30% remain symptomatic despite multiple treatment attempts [4, 5]. Such clinical heterogeneity may reflect the aggregation of distinct conditions with differing etiologies and pathologies under the same diagnostic label. Research has demonstrated that in psychiatric disorders reliance on group-level comparisons and the concept of an "average" patient can be misleading when applied to individuals [6, 7]. To address these challenges, research efforts should aim to characterize the clinical and biological heterogeneity in psychiatric populations.

Despite advances in understanding neuroanatomical heterogeneity in other psychiatric disorders [8–11], this approach has not been applied to patients with MDD. Studies investigating neuroanatomical differences between MDD patients and healthy controls (HCs) have relied on case-control comparisons. Based on structural magnetic resonance imaging (sMRI) analysis software with automated assessment and parcellation of neuroanatomical features [12], studies traditionally compared cortical thickness (CT), cortical surface area (CSA), and subcortical volume (SV) measures between MDD patients and HCs. Seminal research by the Enhancing NeuroImaging Genetics through Meta-Analysis (ENIGMA) consortium’s MDD working group has identified significant neuroanatomical differences between MDD patients and HCs [13, 14]. Patients with MDD show reductions in bilateral hippocampal volumes, as well as the amygdala and striatum [13, 15]. Additionally, decreased CT has been reported in regions including the prefrontal cortex (PFC), orbitofrontal cortex (OFC), anterior and posterior cingulate cortex (ACC and PCC), insular cortex, and temporal gyrus [14]. While these volumetric reductions are consistently detectable, the effect sizes are modest, with Cohen's d ranging from -0.1 to -0.15 [13, 14]. Furthermore, a recent large-scale case-control study reported that neuroanatomical differences between MDD patients and HCs accounted for less than 2% of the variance between the groups [16]. This suggests that neuroanatomical differences are minimal in patients with MDD, with greater similarities to HCs prevailing.

A potential limitation of these research efforts is that, although neuroanatomical differences have been identified, they rely on comparing group-means [17]. This approach inherently assumes within-group homogeneity, implying that mean comparisons accurately represent the distributions of all individuals [18]. Such an assumption overlooks the possibility of increased inter-individual variability (i.e., heterogeneity) within the patient population [19, 20]. Neuroanatomical heterogeneity in MDD could be an explanation for the modest effect sizes observed in previous case–control studies of neuroanatomical differences [21], as averaging across patients with diverse alterations could obscure coherent patterns of structural alteration. Accordingly, shifting the focus from mean-based group comparisons of structural alterations toward analyses of individual variability and neuroanatomical heterogeneity may yield more informative insights into the neurobiological deficits of MDD.

Against this backdrop, the present analysis investigated neuroanatomical heterogeneity in MDD by comparing global and regional measures of structural variability between patients and HCs. Regional heterogeneity was measured using the coefficient of variation ratio (CVR), which quantifies increased variance in specific neuroanatomical metrics (e.g., cortical thickness) within distinct brain regions relative to controls [8]. Global heterogeneity, in turn, was assessed with the person-based similarity index (PBSI), which reflects inter-individual variation in the overall configuration of brain structure by computing the similarity of each individual’s multivariate neuroanatomical pattern to the group-level pattern [22]. Together, these measures provide complementary perspectives on neuroanatomical heterogeneity in depression, extending insights from previous case–control studies of structural alterations [13, 14]. By quantifying inter-individual variability as a continuous property of the data, PBSI and CVR capture both global and region-specific sources of structural variability, offering an efficient and model-free framework for characterizing heterogeneity. Evidence from other psychiatric conditions (e.g., psychosis, schizophrenia, and bipolar disorder) has already shown pronounced increases in global and regional neuroanatomical heterogeneity with these measures [8–11]. Such a characterization, which allows a more nuanced understanding of structural deficits that could provide the foundation for future, more guided stratification approaches, is currently lacking in MDD.

Addressing this gap, this study provides a systematic investigation of neuroanatomical heterogeneity in MDD. We hypothesized that patients with MDD would exhibit increased global and regional neuroanatomical heterogeneity compared to HCs. Prior research has indicated that demographic and clinical variables (e.g., AD use, age at illness onset, and recurrence status) influence neuroanatomical differences between MDD patients and HCs [23]. Therefore, moderation analyses were conducted to assess their influence on neuroanatomical heterogeneity. To examine the clinical relevance of global neuroanatomical heterogeneity, we applied a normative referencing approach to identify MDD patients whose global neuroanatomical variability was markedly increased (<5^th^ percentile in similarity relative to the normative range) [24]. We hypothesized that those patients would exhibit more severe clinical features of depression.

## Materials and methods

### Samples

The study sample was drawn from the ENIGMA MDD Working Group, an international research consortium aggregating neuroimaging and clinical data from individuals diagnosed with MDD and HC volunteers. The initial dataset comprised 9 831 participants (MDD: 4 205; HC: 5 626) from 41 cohorts. After data processing and subsequent matching, 4 685 participants were excluded (MDD: 1 632; HC: 3 053; see below and Supplementary Tables S1–S2 for details), yielding a final analysis sample of 5 146 participants (MDD: 2 573; HC: 2 573). Participants in the analysis sample were aged 9–82 years, and 64% were female. All participating sites obtained local ethics approval, and all participants, or their parents or legal guardians where applicable, provided written informed consent. All methods were performed in accordance with the relevant guidelines and regulations.

### Neuroimaging data acquisition and processing

Structural 3D T1-weighted MRI scans were acquired and processed locally at each site using standard FreeSurfer pipelines [12, 25–27] in accordance with the ENIGMA protocol (https://github.com/ENIGMA-git/). This common processing approach was applied across all participating cohorts, including individuals younger than 18 years; no separate pediatric-specific FreeSurfer pipeline was used in the present consortium dataset. FreeSurfer segmentation yielded 68 regional measures of cortical thickness (CT), 68 of cortical surface area (CSA), and 14 subcortical volumes (SV) based on the Desikan–Killiany atlas [28]. ENIGMA quality control comprised automated outlier detection (±2 SD) and site-level visual inspection; regional measures with evidence of poor segmentation were excluded. Site-specific acquisition parameters are detailed in Supplementary Tables S3–S4.

For the present analysis, participants with >5% missing measures across CT, CSA, and SV were removed as indicative of poor parcellation [9]. Neuroimaging measures for the remaining participants were harmonized separately for CT, CSA, SV using neuroComBat [29], adjusting for site-related batch effects while preserving variance attributable to diagnosis, age, and sex (specified as preserved covariates in the model matrix); code is available at GitHub: https://github.com/lusemp/Decomposing-Neuroanatomical-Heterogeneity-in-Depression-Preprocessing-Harmonization-Code-/tree/main. To assess harmonization performance, multivariate site-effect analyses were performed separately for CT, CSA, and SV before and after harmonization using PERMANOVA and MDMR (999 permutations), and MDS plots were generated to visualize clustering by site (see Supplementary Material, Supplementary Table S5, and Supplementary Fig. S1). After harmonization, MDD participants missing predefined clinical variables (AD use, age at illness onset, recurrence status) were excluded. The final case–control sample was then matched 1:1 on age and sex across cohorts. Additional details on analysis-specific processing steps are described in the Supplementary Material.

### Clinical variables

The clinical variables assessed in this study included AD use (at the time of MRI scanning; present vs. absent), age at illness onset (adolescent-onset [≤21 years] vs. adult-onset [>21 years]), recurrence status (first episode vs. recurrent), remission status (acutely depressed [episode within the past 6 months] vs. remitted), depression severity, and number of depressive episodes. Depression severity was indexed by the clinician-rated Hamilton Depression Rating Scale-17 (HDRS-17; total score) and the self-report Beck Depression Inventory-II (BDI-II; total score). As an additional severity indicator, the number of DSM-IV MDD criteria met from a structured interview was used. Variables were collected by local sites according to the ENIGMA MDD Working Group protocol and operationalized in line with prior work [13, 14].

### Statistical analyses

Prior work from the ENIGMA MDD working group on a partially overlapping sample documented case–control mean differences in neurostructural measures [13, 14]. Here, these findings are extended by quantifying inter-individual neuroanatomical heterogeneity in MDD using cross-sectional clinical and neuroimaging data. Statistical analyses were conducted in R (4.3.2) and MATLAB (R2022b, 9.13). All statistical tests were two-sided, and false discovery rate correction was applied where appropriate.

#### Group-level global heterogeneity – person-based similarity index (PBSI)

Subject-specific estimates of inter-individual variability within each group (MDD or HC) were quantified using the PBSI [22]. PBSI scores were calculated separately for CT, CSA, and SV, using the concatenation of all regional measures of each neuroanatomical metric. This concatenation created a global brain profile per subject and metric, and the PBSI score for each subject was computed as the average of all pairwise Spearman correlations between that subject’s brain profile and the brain profiles of all other subjects within the same group (see formula below). The PBSI score reflects how similar an individual’s global brain profile is to all other members of the same group. PBSI scores range from 0 to 1, with higher scores indicating greater neuroanatomical similarity to others in the group. The function used to compute the PBSI scores is available at: https://www.mathworks.com/matlabcentral/fileexchange/69158-similarityscore. To investigate whether group differences in heterogeneity varied across the three neuroanatomical metrics (CT/CSA/SV), a linear mixed-effects model was used, with PBSI score as the dependent variable and Group (MDD/HC), Metric (CT/CSA/SV), and their interaction (Group × Metric) as fixed effects. The model included the covariates age and sex as well as a random intercept for participant (1 | ID). Post-hoc comparisons were conducted using estimated marginal means for pairwise group comparisons, adjusting for multiple comparisons using the false discovery rate (FDR). Effect sizes of the global neuroanatomical heterogeneity differences between MDD patients and HCs were calculated using Cohen's d.

$${{PBSI}}_{i}=\frac{1}{N-1}\mathop{\sum }\limits_{\begin{array}{c}j=1\\ j\ne i\end{array}}^{N}{cor}\left({y}_{i},{y}_{j}\right)$$

The PBSI_i_ score is the average Spearman correlation between all regional measures of the i^th^ individual (y_i_) and the measures of all other individuals of the same reference group (y_j_, for j ≠ i). For a given participant i, y_i_ represents the global brain profile (vector of all regional measures for a given neuroanatomical metric [CT, CSA or SV]).

#### Group-level regional heterogeneity – coefficient of variation ratio (CVR)

The CVR was calculated separately for all regional measures of CT, CSA, and SV to estimate regional differences in inter-individual variability between the MDD and HC groups. CVR analyses were conducted using the natural logarithm of the CVR as a mean-scaled measure of group-level heterogeneity differences (see formula below). The CVR was computed using the escalc() function from the *metafor* package [30], and summarized in forest plots adjusting for multiple comparisons using the FDR within each neuroanatomical metric. To aid interpretation, log-transformed CVRs were back-transformed to the original scale; a CVR of 1 indicates no difference in heterogeneity between MDD and HC groups, a CVR > 1 suggests greater heterogeneity in MDD, and a CVR < 1 indicates greater heterogeneity in HCs. The CVR serves as a direct measure of effect size, quantifying the percentage increase or decrease in variation between groups for a given regional measure. However, no standardized thresholds exist for interpreting the magnitude of CVRs in psychiatric neuroimaging studies.

$${\mathrm{ln}}\,{CVR}={\mathrm{ln}}\left(\frac{{\hat{{\rm\sigma }}}_{{\rm{MDD}}}/{\overline{\rm{X}}}_{{\rm{MDD}}}}{{\hat{{\rm\sigma }}}_{{\rm{HC}}}/{\overline{\rm{X}}}_{{\rm{HC}}}}\right)={\mathrm{ln}}\left(\frac{{{\rm{S}}}_{{\rm{MDD}}}/{\overline{\rm{X}}}_{{\rm{MDD}}}}{{{\rm{S}}}_{{\rm{HC}}}/{\overline{\rm{X}}}_{{\rm{HC}}}}\right)+\frac{1}{2\left({n}_{{MDD}}-1\right)}-\frac{1}{2\left({n}_{{HC}}-1\right)}$$

The CVR was computed using the following values: the group means (${\bar{{\rm{X}}}}_{{\rm{MDD}}}$ and ${\bar{{\rm{X}}}}_{{\rm{HC}}}$) and the unbiased population standard deviations for each group (${\hat{{\rm{\sigma }}}}_{{\rm{MDD}}}$ and ${\hat{{\rm{\sigma }}}}_{{\rm{HC}}}$), based on the respective sample sizes (n_MDD_ and n_HC_) and standard deviations (S_MDD_ and S_HC_) of the groups.

#### Moderation analyses of group-level heterogeneity effects

Demographic (age, sex) and clinical (AD use, recurrence status, age at illness onset) variables were examined as moderators of group differences in neuroanatomical heterogeneity at both the global (PBSI) and regional (CVR) levels.

For global heterogeneity, two complementary ANOVA models were specified. First, a Type III ANOVA in the full sample tested the influence of demographic variables, with PBSI as the dependent variable and Group (MDD/HC), Age (continuous), Sex (male/female), and their interactions (Group × Age, Group × Sex) as predictors. For significant effects, post hoc comparisons were performed using estimated marginal means, adjusting for multiple comparisons using the FDR, and effect sizes were expressed as Cohen’s d. Second, a Type II ANOVA restricted to the MDD group tested the influence of clinical variables, with PBSI as the dependent variable and AD use (yes/no), recurrence status (first-episode/recurrent), and age at illness onset (adolescent/adult) as predictors, adjusting for Age and Sex. For significant effects, post hoc comparisons were performed using estimated marginal means, adjusting for multiple comparisons using the FDR, and effect sizes were expressed as Cohen’s d.

For regional heterogeneity, MDD patients were stratified by each moderator (with age dichotomized as adolescent [≤21 years] vs. adult [>21 years]). Subgroup-specific CVRs were then estimated by contrasting each MDD subgroup with its matched HC subsample, resulting in separate CVRs for each subgroup–control comparison. The FDR was used to control for multiple comparisons.

#### Patient-specific global variability – Norm-PBSI

Norm-PBSI scores for the MDD group were computed separately for CT, CSA, and SV. The analysis followed the same procedure as the standard PBSI computation, with one key difference: for the norm-PBSI, each MDD patient’s global brain profile was correlated with the global brain profiles of all HCs, which served as the normative reference set. The resulting norm-PBSI quantified each patient’s similarity to the HC reference set, with lower values indicating greater neuroanatomical dissimilarity (i.e., higher variability relative to the normative range). To classify MDD patients according to their deviation from the HC normative range, norm-PBSI values were z-standardized to the HC reference distribution, and patients were categorized using the 5^th^ and 95^th^ percentiles as thresholds [24]:

I. *norm*: scores between the 5^th^ and 95^th^ percentiles (neuroanatomical variability within the HC normative range);
II. *supra-norm*: scores above the 95^th^ percentile (markedly reduced neuroanatomical variability relative to the HC norm);
III. *infra-norm*: scores below the 5^th^ percentile (markedly increased neuroanatomical variability relative to the HC norm-range).

Pairwise comparisons among the *norm*, *supra-norm*, and *infra-norm* groups were conducted to assess demographic and clinical differences. Continuous variables were compared using the Mann–Whitney U test, dichotomous variables using the chi-squared test, and effect sizes for continuous contrasts were expressed as Cohen’s d.

## Results

Neuroanatomical heterogeneity in MDD was assessed in a harmonized, age- and sex-matched case–control sample of 5 146 participants (MDD: 2 573, HC: 2 573). Detailed demographic and clinical characteristics of the analysis sample are presented in Table 1.

**Table 1:** Demographics and clinical characteristics of the final study sample (1:1 matched for age and sex across sites).

Feature	MDD Patients (n = 2573)	Healthy Controls (n = 2573
*Sample composition*		
Total participants, n	5146	
Included cohorts, n	41	
*Demographics (both groups)*		
Age (years), mean (SD)	40.57 (15.13)	
Sex, female/male, n (%)	1651 (64) / 922 (36)	
*Clinical features (MDD only)*		
Age at onset of illness (years), mean (SD)	29.88 (14.27)	-
Antidepressant use at time of scanning, n (%)	Yes: 1405 (55) No: 1168 (45)	-
Recurrence status, n (%)	First episode: 1 028 (40) Recurrent: 1 545 (60)	-
HDRS-17, mean (SD)	13.87 (8.16)*	-
BDI-II, mean (SD)	19.50 (12.12)*	-
Number of DSM-IV criteria for MDD met, mean (SD)	4.79 (2.77)*	-

* Missing data: HDRS-17 = 47.1%; BDI-II = 36.7%; DSM-IV criteria = 60.4%.

*n* number, *SD* standard deviation, *MDD* major depressive disorder, *HDRS-17* hamilton depression rating scale-17, *BDI-II* beck depression inventory-II, *DSM-IV* diagnostic and statistical manual of mental disorders, fourth edition.

### Neuroanatomical heterogeneity in depression – global (PBSI): increased only in cortical thickness

**Fig. 1:**
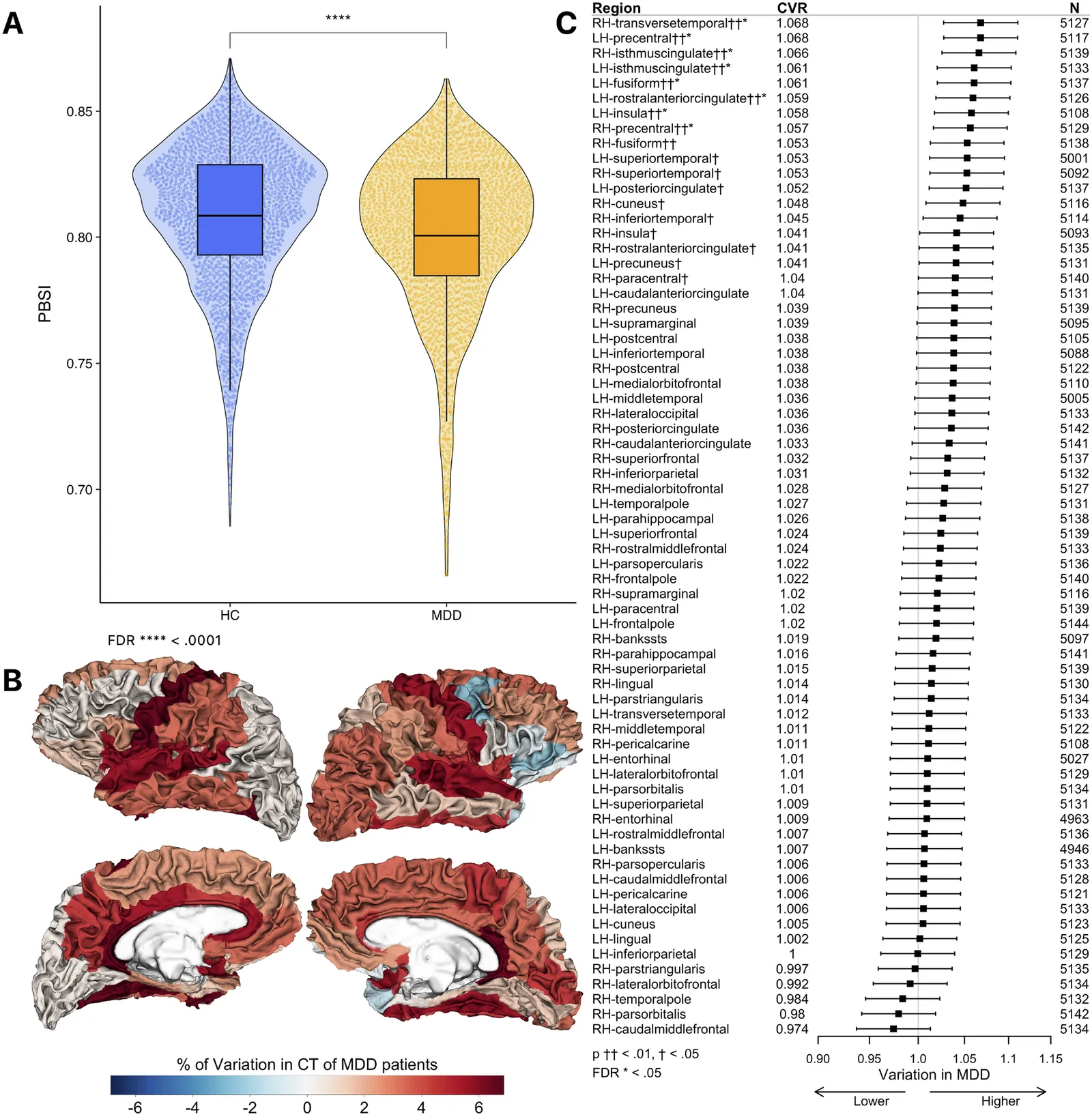
Global and regional cortical thickness heterogeneity in MDD compared with HCs. **A** Violin and box plots of global cortical thickness heterogeneity quantified using the person-based similarity index, in MDD patients and age- and sex-matched HCs (median and interquartile range). **B** Brain maps illustrating the percentage of variation in cortical thickness among MDD patients relative to HCs, with color intensity reflecting the magnitude and direction of effects (red = higher, blue = lower variation). **C** Forest plot of the coefficient of variation ratio for cortical thickness across brain regions, where CVR > 1 indicates greater heterogeneity in MDD. CVR = coefficient of variation ratio; CT = cortical thickness; FDR = false discovery rate; HC = healthy control; LH = left hemisphere; MDD = major depressive disorder; PBSI = person-based similarity index; RH = right hemisphere.

MDD patients showed significantly greater global CT heterogeneity compared with HCs, reflected by a lower mean PBSI score (PBSI_MDD_ = 0.801, PBSI_HC_ = 0.809, P_FDR_ < 0.001, d = −0.26, 95% CI [ − 0.32, −0.21]) (Fig. 1A). The averaged PBSI score indexes the degree of global neuroanatomical similarity within a group, with lower mean PBSI values indicating reduced within-group similarity and, consequently, greater global heterogeneity [11]. No group differences were observed in global neuroanatomical similarity for CSA or SV (Supplementary Fig. S2; Supplementary Table S6).

### Neuroanatomical heterogeneity in depression – regional (CVR): increased only in cortical thickness, confined to cingulate, insular, frontal, and temporal regions

Regional analyses revealed focal increases in CT heterogeneity in MDD compared with HCs. Significant effects were observed in the frontal and temporal lobes, as well as the cingulate and insular cortices (CVR > 1, P_FDR_ < 0.05) (Fig. 1B). Regions surviving multiple-comparison correction included the right transverse temporal gyrus (+6.8% more variation, 95% CI [2.7, 11.1]), bilateral precentral gyrus (left: +6.8%, 95% CI [2.7, 11.0]; right: +5.7%, 95% CI [1.6, 9.8]), bilateral isthmus cingulate cortex (left: +6.1%, 95% CI [2.0, 10.3]; right: +6.6%, 95% CI [2.5, 10.9]), left fusiform gyrus (+6.1%, 95% CI [2.0, 10.3]), left rostral anterior cingulate cortex (+5.9%, 95% CI [1.9, 10.2]), and left insula (+5.8%, 95% CI [1.7, 10.0]) (Supplementary Table S7). The CVR quantifies regional heterogeneity as the relative variability in patients versus controls and can be interpreted as the percentage increase in variation observed in patients [31]. The significant CT effects clustered within a relatively narrow range (5.7%–6.8% higher variability in MDD), indicating similar effect sizes across the affected regions. No group differences in regional heterogeneity were observed for CSA or SV after FDR correction (Supplementary Figs. S3, S4).

### Moderation analysis of demographic factors: stable patterns without age- or sex-specific effects on cortical thickness heterogeneity in depression

For global CT heterogeneity (PBSI), the Type III ANOVA revealed significant main effects of group (p < 0.001), age (p < 0.01), and sex (p < 0.01), with no significant group × age (p = 0.567) or group × sex (p = 0.064) interactions (Supplementary Table S8). Subgroup contrasts confirmed higher global CT heterogeneity in MDD relative to matched HCs across all comparisons: adults (N = 4 490 [MDD: 2 245, HC: 2 245]; P_FDR_ < 0.001, d = −0.27, 95% CI [ − 0.32, −0.21]), adolescents (N = 656 [MDD: 328, HC: 328]; P_FDR_ < 0.001, d = −0.25, 95% CI [ − 0.41, −0.10]), males (N = 1 844 [MDD: 922, HC: 922]; P_FDR_ < 0.01, d = −0.35, 95% CI [ − 0.44, −0.26]), females (N = 3 302 [MDD: 1 651, HC: 1 651]; P_FDR_ < 0.001, d = −0.22, 95% CI [ − 0.29, −0.15]) (Supplementary Tables S9–S10).

**Fig. 2:**
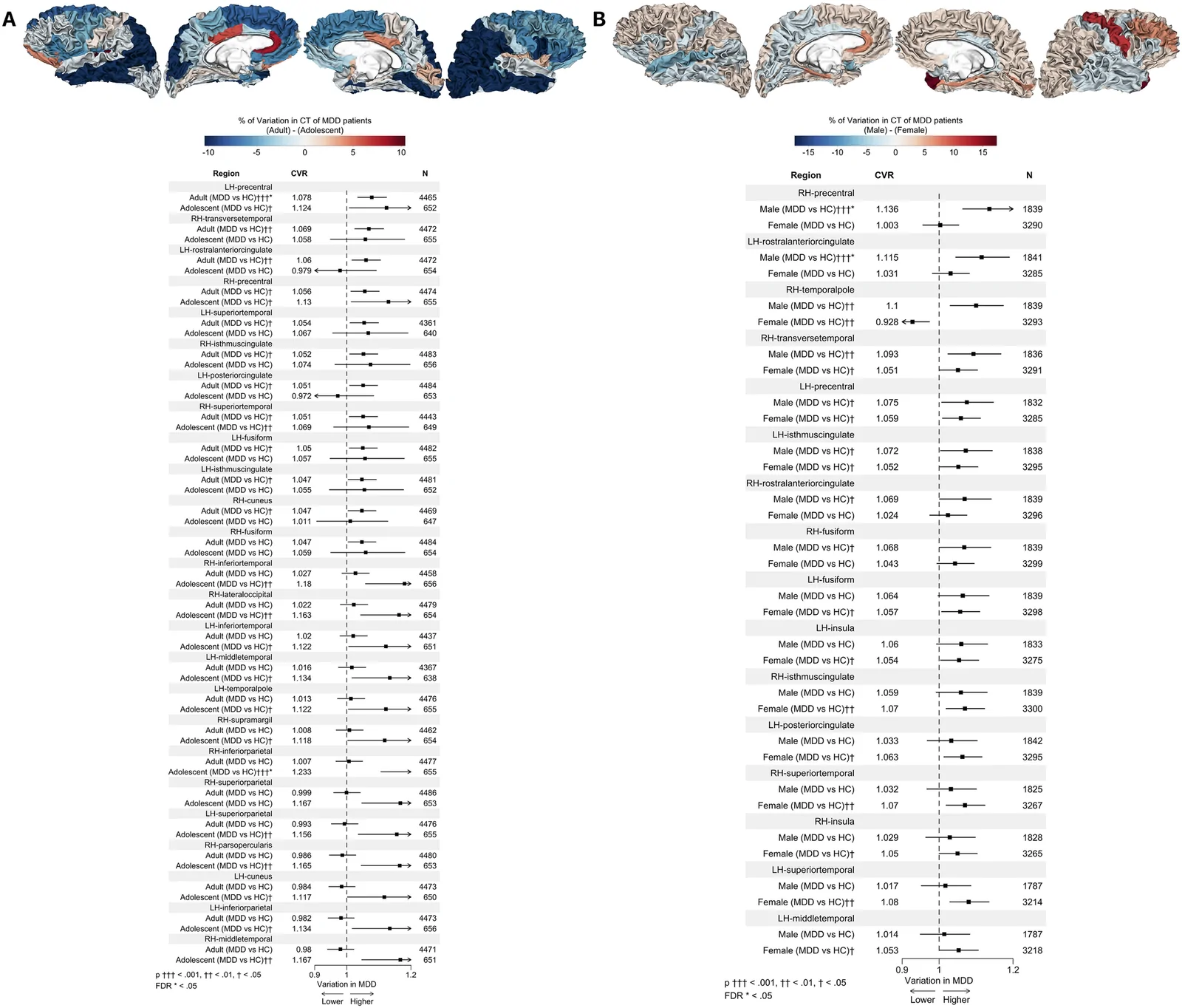
Age and sex effects on regional cortical thickness heterogeneity in MDD compared with HCs. **A** Forest plots and brain maps showing significant coefficients of variation ratio values (P_FDR_ < 0.05) for cortical thickness heterogeneity in adult (> 21 years) and adolescent (≤ 21 years) MDD subgroups relative to age- and sex-matched HCs. **B** Forest plots and cortical surface maps showing significant coefficients of variation ratio values (P_FDR_ < 0.05) for cortical thickness heterogeneity in male and female MDD subgroups relative to age-matched HCs. For both (**A**) and **B**, CVR > 1 indicates greater heterogeneity in MDD, and color intensity represents the magnitude of the percentage difference in variation between subgroups (red = higher, blue = lower variation). CVR = coefficient of variation ratio; CT = cortical thickness; HC = healthy control; LH = left hemisphere; MDD = major depressive disorder; RH = right hemisphere.

For regional CT heterogeneity (CVR), significant focal increases emerged after FDR correction (CVR > 1, P_FDR_ < 0.05). Specifically, adults with MDD showed greater heterogeneity than adult HCs in the right precentral gyrus (+7.8%, 95% CI [3.4, 12.4]); adolescents with MDD showed greater heterogeneity in the inferior parietal cortex (+23.3%, 95% CI [10.6, 37.5]); and males with MDD exhibited increased heterogeneity in the right precentral gyrus (+13.6%, 95% CI [6.4, 21.2]) and left rostral anterior cingulate cortex (+11.5%, 95% CI [4.5, 19.0]). No regional effects were observed in females with MDD after FDR correction (Fig. 2A–B; Supplementary Tables S11–S14; Supplementary Figs. S5–S6).

### Moderation analysis of clinical factors: antidepressant use linked to greater cortical thickness heterogeneity in depression

**Fig. 3:**
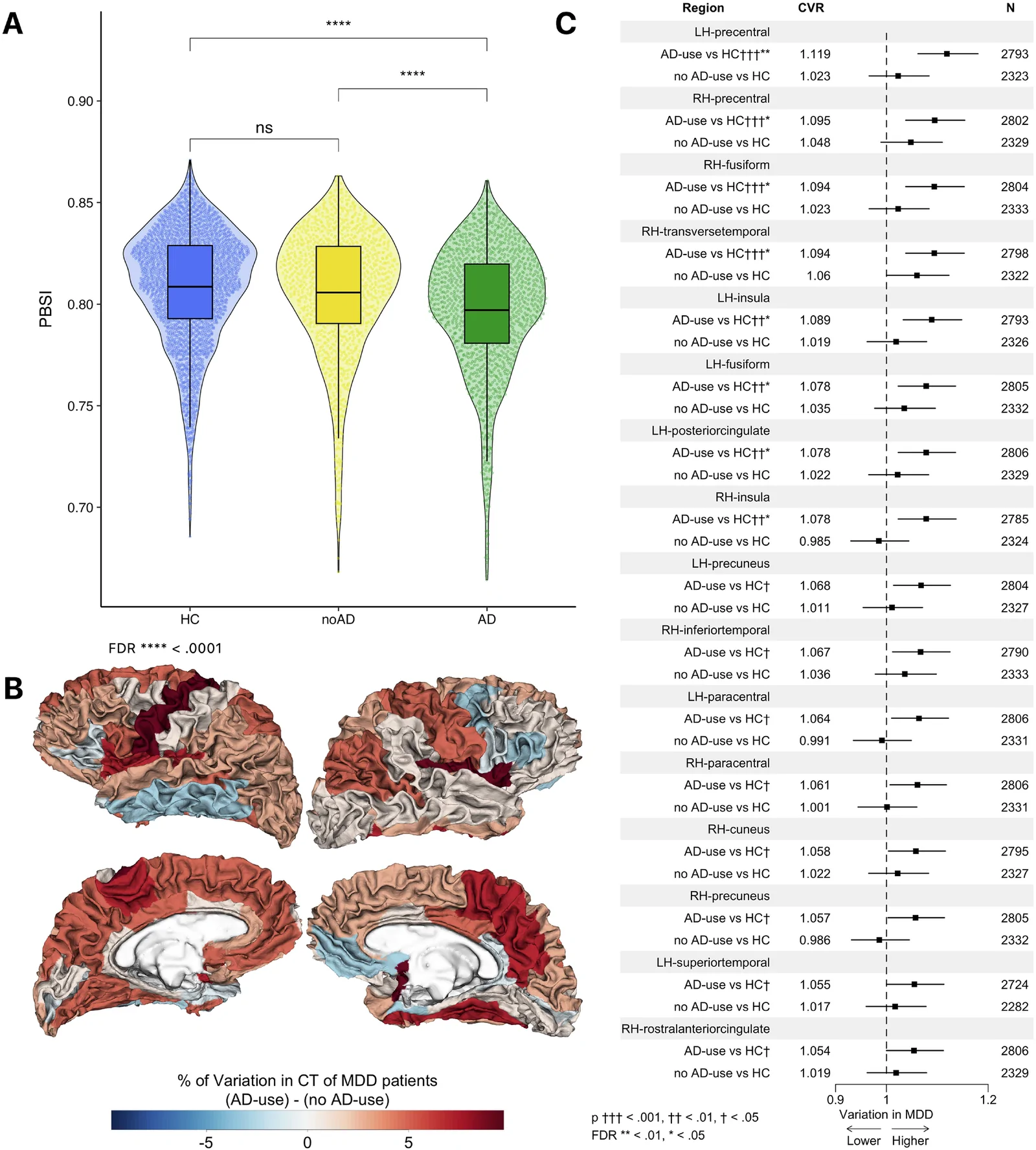
Effect of antidepressant use on global and regional cortical thickness heterogeneity in MDD compared with HCs. **A** Violin and box plots of global cortical thickness heterogeneity (PBSI scores) in MDD subgroups with and without antidepressant use compared with age- and sex-matched HCs (median and interquartile range). **B** Forest plots showing significant coefficients of variation ratio values (P_FDR_ < 0.05) for cortical thickness heterogeneity in AD-use and no-AD-use subgroups compared with HCs, where CVR > 1 indicates greater heterogeneity in MDD. **C** Brain maps illustrating differences in cortical thickness heterogeneity between AD-use and no-AD-use MDD patients relative to HCs, with color intensity reflecting the percentage difference in variation between subgroups (red = higher, blue = lower variation). AD = antidepressant; CVR = coefficient of variation ratio; CT = cortical thickness; FDR = false discovery rate; HC = healthy control; IQR = interquartile range; LH = left hemisphere; MDD = major depressive disorder; PBSI = person-based similarity index; RH = right hemisphere.

For global cortical thickness (CT) heterogeneity (PBSI), the MDD-only Type II ANOVA revealed a significant main effect of AD use (p < 0.001), whereas recurrence status (p = 0.364) and age at illness onset (p = 0.187) were not significant (Supplementary Table S15; Supplementary Figs. S8–S11). Subgroup contrasts indicated greater global heterogeneity in MDD patients using antidepressants (N = 1 405) compared with HCs (N = 2 573; P_FDR_ < 0.001, d = −0.39, 95% CI [ − 0.45, −0.32]), and with MDD patients without antidepressants (N = 1 168; P_FDR_ < 0.001, d = −0.27, 95% CI [ − 0.34, −0.20]). In contrast, MDD patients without antidepressants did not differ significantly from HCs (P_FDR_ = 0.056). (Fig. 3A; Supplementary Table S16).

For regional CT heterogeneity (CVR), significant focal increases emerged for MDD patients with antidepressants versus matched HCs after FDR correction (CVR > 1, P_FDR_ < 0.05) (Fig. 3B). Specifically, higher heterogeneity was observed in the bilateral precentral gyrus (left: +11.9%, 95% CI [6.2, 18.0]; right: +9.5%, 95% CI [3.8, 15.4]), bilateral fusiform gyrus (left: +7.8%, 95% CI [2.3, 13.6]; right: +9.4%, 95% CI [3.8, 15.3]), right transverse temporal gyrus (+9.4%, 95% CI [3.8, 15.3]), bilateral insula (left: +8.9%, 95% CI [3.3, 14.8]; right: +7.8%, 95% CI [2.3, 13.7]), and left posterior cingulate cortex (+7.8%, 95% CI [2.3, 13.6]) (Fig. 3C; Supplementary Table S17). No regional CVR differences survived FDR correction for MDD without antidepressants versus matched HCs (Fig. 3C; Supplementary Table S18), nor for MDD with versus without antidepressants (Supplementary Fig. S7).

### Patient-specific global variability (Norm-PBSI): no differences in the number of norm-deviating patients across neuroanatomical metrics

Based on the individual norm-PBSI classification, 20% of MDD patients (N = 509) were identified as *infra-norm* deviators, showing marked neuroanatomical dissimilarity to HCs (<5^th^ percentile) in at least one neuroanatomical metric (N_CT_ = 211, N_CSA_ = 187, N_SV_ = 229), including 49 patients deviating in multiple metrics and 3 in all three. In contrast, 78% of MDD patients (N = 1 965) were within the normative range (5^th^–95^th^ percentile), and 2% (N = 49) were classified as *supra-norm* deviators, showing greater similarity to HCs (>95^th^ percentile) in a single metric (N_CT_ = 14, N_CSA_ = 34, N_SV_ = 1), with no patients deviating in more than one metric (Supplementary Fig. S12).

### Patient-specific global variability (Norm-PBSI): infra-norm deviation in cortical thickness linked to higher symptom severity

**Fig. 4:**
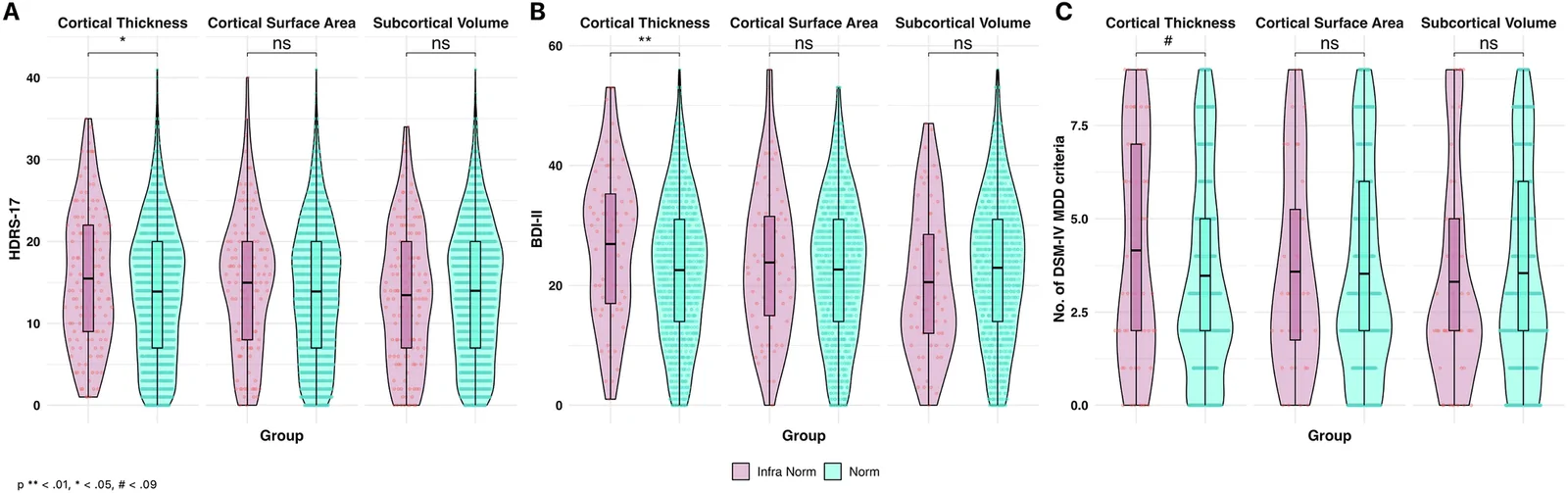
Depression severity by neuroanatomical variability in MDD. Violin and box plots comparing depression severity between *infra-norm* MDD patients (with markedly increased dissimilarity in structural variability patterns to HCs, < 5^th^ percentile; pink) and *norm* MDD patients (with similar structural variability patterns to HCs, 5t^h^–95^th^ percentile; turquoise) across measures of cortical thickness, cortical surface area, and subcortical volume. Panels show (**A**) HDRS-17 total scores, (**B**) BDI-II total scores, and (**C**) number of DSM-IV MDD criteria met. BDI-II = Beck Depression Inventory-II; CT = cortical thickness; CSA = cortical surface area; DSM-IV = Diagnostic and Statistical Manual of Mental Disorders, Fourth Edition; HC = healthy control; HDRS-17 = Hamilton Depression Rating Scale-17; MDD = major depressive disorder; SV = subcortical volume.

Patients identified as *infra-norm* deviators for CT (N = 211) showed significantly higher depression severity compared with *norm* patients for CT (N = 2 331). *Infra-norm* classification indicates markedly increased global neuroanatomical variability, meaning that CT patterns in these patients deviated markedly from the HC reference range. Specifically, *infra-norm* patients exhibited higher clinician-rated symptom scores (HDRS-17: mean difference = 1.59, p < 0.05, d = 0.19, 95% CI [0.02, 0.37]) and self-reported symptom scores (BDI-II: mean difference = 4.34, p < 0.01, d = 0.36, 95% CI [0.11, 0.63]), as well as a trend-level difference in the number of DSM-IV criteria met for MDD (mean difference = 0.68, p = 0.065, d = 0.25, 95% CI [–0.03, 0.54]) (Fig. 4). No significant differences in depression severity were observed for *supra-norm* deviators for CT (Supplementary Table S19).

A sex difference was also observed between the *infra-norm* and *norm* groups for CT (χ² = 5.14, p < 0.05), with the *infra-norm* group showing a higher proportion of females (71%) compared with the *norm* group (62%) (Supplementary Table S19). No significant differences in sex distribution were observed for the supra-*norm* group for CT, and no additional clinical or demographic differences were found among the *infra-norm*, *norm*, and *supra-norm* groups for CT (Supplementary Table S19).

Detailed results of subgroup contrasts between *infra-norm*, *norm*, and *supra-norm* patients for CSA and SV are provided in Supplementary Tables S20–S21.

## Discussion

The aim of this study was to investigate global and regional neuroanatomical heterogeneity in MDD compared with HCs across three structural metrics: CT, CSA, and SV. In addition, patient-specific variability relative to the normative range defined by the HC group was examined to explore the potential clinical relevance of increased neuroanatomical variability.

Three key findings emerged: First, MDD patients showed greater global (whole-brain) and regional (region-specific) heterogeneity in CT, whereas no such differences were observed for CSA or SV. Second, increased CT heterogeneity (global & regional) was most pronounced among patients receiving AD medication at the time of scanning, while AD-free patients and HCs displayed comparable levels of CT heterogeneity. Third, patients exhibiting markedly increased CT variability (<5^th^ percentile relative to the normative range) showed higher depressive symptom severity compared with patients whose CT variability fell within the normative range.

The finding of increased global CT heterogeneity in MDD extends prior work showing widespread case–control CT differences [14, 15, 32, 33]. The absence of similar effects for CSA or SV suggests that CT may be the most dynamic structural marker of disorder-related variation. CT is known to be particularly sensitive to neuroplastic processes such as synaptic remodeling, dendritic pruning, and glial changes, all mechanisms that have been repeatedly implicated in the pathophysiology of depression [23]. In contrast, CSA and SV represent more stable neuroanatomical traits, reflecting early neurodevelopmental or genetic influences rather than state-dependent alterations [34–37]. This distinction aligns with evidence that CSA is more heritable and potentially less influenced by environmental or illness-related factors compared to CT [35, 36].

Regionally, increased CT heterogeneity in MDD was most pronounced in the precentral, transverse temporal, and fusiform gyri, as well as in the isthmus and rostral anterior cingulate and insular cortices, brain areas consistently implicated in both structural and functional alterations in depression [14, 38–40]. These regions support diverse functions spanning motor control, sensory integration, and emotion regulation, suggesting that structural heterogeneity in MDD may arise across diverse yet interacting functional domains. Previous meta-analyses have reported predominantly cortical thinning in these regions, though localized CT increases, particularly in the transverse temporal gyrus and posterior cingulate cortex, have also been observed [41, 42]. Such bidirectional changes in CT could help explain the observed heterogeneity and may reflect region-specific variability in neuroplastic adaptations to illness progression or treatment exposure. This interpretation aligns with evidence suggesting that CT changes may vary in relation to different behavioral manifestations. For example, variability in the precentral gyrus may correspond to individual differences in psychomotor symptoms such as retardation or agitation [43], whereas heterogeneity in the fusiform gyrus and cingulate cortex may relate to altered perceptual or emotional processing [44, 45]. Although such interpretations remain largely speculative at this stage, the present findings nonetheless suggest that CT heterogeneity in certain regions may reflect variable structural adaptations rather than uniform cortical changes. Whether this variability represents distinct pathophysiological mechanisms, treatment-related effects, or downstream consequences of symptom expression remains to be determined.

Beyond the overall increase in CT heterogeneity in MDD, moderation analyses identified AD use as a key factor contributing to structural variability in CT. Only patients receiving ADs at the time of scanning showed greater global and regional CT heterogeneity, whereas non-medicated patients and HCs exhibited comparable levels. This pattern may reflect inter-individual differences related to treatment exposure. ADs have been shown to promote neuroplastic processes in mood-regulating regions such as the frontal lobe and cingulate cortex [46, 47]. Regionally, increased CT heterogeneity in AD-treated patients was most pronounced within precentral, fusiform, and insular regions, aligning with previous reports linking structural and metabolic alterations in these areas to antidepressant response and remission [48, 49]. However, the current cross-sectional data do not allow any causal or mechanistic inferences. AD-treated patients were also older, more symptomatic, and more often recurrent, clinical features previously associated with structural brain alterations in MDD [14, 19]. Although the groups were age and sex matched and analyses adjusted for clinical and demographic factors, residual confounding by unmeasured variables such as concomitant or lifetime medication exposure cannot be ruled out. In particular, antipsychotic augmentation, which has been shown to affect CT in other psychiatric disorders [50], may have contributed to the observed CT variability. Therefore, the present findings should be interpreted with caution. Rather than representing a unitary effect of ADs, the observed increase in structural variability likely reflects a complex interplay between treatment-related processes and greater illness burden, suggesting that multiple factors jointly shape CT variability in MDD. Prospective and longitudinal studies will be crucial to disentangle these influences and clarify the mechanisms underlying increased CT heterogeneity in AD-treated patients.

To examine the clinical relevance of neuroanatomical variability at the individual level, a normative referencing approach was applied to identify patients exhibiting markedly increased structural variability relative to the HC range. Across all three neuroanatomical metrics, 78% of patients remained within the normative range, suggesting that pronounced neuroanatomical deviation was absent in most individuals with MDD. This was consistent with the absence of significant heterogeneity differences for CSA and SV. Although CT heterogeneity was increased in MDD, most patients also remained within the normative range for CT, suggesting a subtle group-level shift and/or greater dissimilarity concentrated in a subset of patients rather than widespread marked deviation. Patients with markedly increased CT variability showed significantly higher symptom severity compared to those within the normative range. This pattern was consistent across both clinician-rated (HDRS-17) and self-reported (BDI-II) measures of depression, suggesting that patients with the most atypical CT profiles relative to controls tend to experience more severe depressive symptomatology. Prior studies have reported both cortical thinning and thickening in relation to depressive symptom severity, reflecting potentially divergent neuroplastic mechanisms that encompass both degenerative and compensatory processes [51–53]. Accordingly, greater CT variability in more severely affected patients may capture the interplay of these opposing structural dynamics across individuals. Notably, this link between patient-level variability and symptom severity was specific to CT and not observed for CSA or SV, reinforcing the view that cortical thickness is particularly sensitive to dynamic neuroplastic processes in depression [32, 54]. However, these findings represent only an initial step, and further work is needed to clarify the clinical relevance of variable CT alterations in depression.

This study is limited by its cross-sectional design, which precludes conclusions about causality or temporal dynamics in neuroanatomical variation. A further limitation relates to the matching procedure, which was performed at the pooled rather than within-site level in order to retain sufficient sample size. In addition, FreeSurfer version varied across cohorts and was closely linked to site, such that some residual processing- and site-related influences may remain, despite validation analyses indicating marked attenuation of site-related multivariate structure after harmonization. Harmonization was performed using neuroComBat, although the broad age range means that nonlinear age effects cannot be fully excluded. Future lifespan studies may therefore benefit from harmonization approaches such as ComBat-GAM, which explicitly model nonlinear age effects. Interpretation of clinical factors associated with greater CT heterogeneity is further limited by the lack of detailed information on AD class, dosage, duration, prior exposure, and concurrent medications. Moreover, the comparison between medicated and non-medicated patients is constrained by the likelihood that the non-medicated group included both antidepressant-naïve and previously treated individuals, limiting attribution of the observed effects in AD-treated patients to direct pharmacological influences. Limited item-level clinical data across sites further precluded dimensional analyses of specific symptom domains in relation to neuroanatomical heterogeneity.

Despite these limitations, several methodological strengths support the findings. The large sample size provided substantial statistical power and broad representation across cohorts. Standardized ENIGMA MRI processing and quality-control procedures enhanced comparability and reproducibility of the imaging measures. Age- and sex-matching improved the precision of case–control comparisons, and the additional harmonization validation analyses supported effective attenuation of site-related multivariate structure. Finally, this study provides one of the first large-scale characterizations of neuroanatomical heterogeneity in MDD and extends prior case–control work through complementary global, regional, and patient-specific measures of neurostructural variability.

Taken together, this study advances understanding of structural brain alterations in MDD by highlighting increased heterogeneity in CT. Future neuroimaging research should build on these findings by disentangling sources of neurostructural variability through item-level symptom data and transdiagnostic frameworks that map specific symptom dimensions onto patterns of neuroanatomical change. Prospective multimodal studies using advanced computational approaches will be crucial to determine whether the CT variability observed here reflects a dynamic process linked to illness course and treatment, and whether distinct biological or clinical subtypes can be identified within these heterogeneous patterns. Recent advances in data-driven phenotyping provide promising directions for such efforts [55–57].

## Supplementary information

Supplementary Material

Supplementary Tables

Supplementary Figures

## Data Availability

The individual-level datasets analysed during the current study are not publicly available due to participant confidentiality, privacy restrictions, and cohort-specific data-sharing agreements.
