# Supplementary material for: Decomposing neuroanatomical heterogeneity in depression: insights from an ENIGMA major depressive disorder working group study in 5146 individuals
Source: Transl Psychiatry. 2026 Jun 23;16:452. doi: 10.1038/s41398-026-04189-x (PMC13547417; doi:10.1038/s41398-026-04189-x)
Supplement: Supplementary file 1 [file 41398_2026_4189_MOESM1_ESM.docx]

**Decomposing neuroanatomical heterogeneity in depression – Supplementary Material**

**Neuroimaging sample exclusion at scanning site**

At each scanning site, neuroimaging experts visually inspected image segmentations by overlaying segmentation labels on T1-weighted scans. Site-wide statistical measures (means, standard deviations, and histograms) were generated to identify non-normal distributions and significant outliers. Samples deviating more than 2.698 standard deviations from the global mean, falling outside the 99th percentile, were flagged as statistical outliers^1,2^. Each site re-inspected flagged cases to confirm accurate segmentation. Properly segmented samples were retained, while inaccurately segmented outliers were excluded.

**Preprocessing and exclusion criteria for harmonization**

The initial dataset, derived from 41 sites, included N=9,831 individuals (MDDs: 4 205; Controls: 5 626). Individual FreeSurfer features across cortical thickness (CT), cortical surface area (CSA), and subcortical volume (SV) metrics were assessed, and samples with >5% missing data, indicating possible parcellation or reliability issues, were excluded. Following deduplication and the removal of individuals with values other than HC/MDD for diagnosis, male/female for sex, and numeric values for age, N=1 314 participants (MDD: 564; HC: 750) were excluded, yielding a final preprocessed dataset of N=8 517 individuals (MDD: 3 641; HC: 4 876).

**Harmonization of neuroimaging sample**

The preprocessed dataset (N=8 517) was harmonized separately for each neuroanatomical metric (CT, CSA, SV) using neuroComBat^3^, an enhanced version of ComBat^4^, to adjust for site-specific effects (e.g., scanner type, protocol variations, FreeSurfer version) while preserving covariate effects (age, sex, diagnosis). This approach effectively reduces site-related variability while retaining meaningful biological variance related to demographics and diagnostic status^5^. R code for the harmonization procedure is available on GitHub (<https://github.com/lusemp/Decomposing-Neuroanatomical-Heterogeneity-in-Depression-Preprocessing-Harmonization-Code-/tree/main>), no additional data processing or imputation was needed.

**Validation of harmonization**

To assess whether harmonization effectively attenuated residual site-related batch effects, we performed multivariate validation analyses separately for CT, CSA, and SV before and after neuroComBat harmonization. For each modality, the full set of regional features was examined using distance-based analyses with scanning site specified as the grouping factor. Residual site effects were quantified using permutational multivariate analysis of variance (PERMANOVA) and multivariate distance matrix regression (MDMR), each based on 999 permutations, to estimate the proportion of multivariate variance attributable to site. In addition, multidimensional scaling (MDS) plots were generated to visualize the extent of clustering by site in the raw and harmonized data. Together, these analyses allowed us to determine whether the multivariate structure of the data remained driven by site after harmonization.

Across all three modalities, site-related multivariate effects were markedly reduced following neuroComBat harmonization, indicating substantial attenuation of batch-related variance while preserving the data for downstream case-control analyses. Detailed results are provided in Supplementary Table S5, and corresponding MDS visualizations are shown in Supplementary Figures S1–S3.

**Matching and exclusion criteria for analysis**

From the harmonized sample (N=8 517), patients with MDD with missing clinical information on current antidepressant use (at time of scanning), recurrence status, or age of onset were excluded (N=937), enhancing the clinical applicability of findings. To control for age and sex effects, the remaining sample (N =7 580; MDD: 2 704; HC: 4 876) was 1:1 matched on age and sex across all cohorts using exact matching. This approach involves pairing each MDD participant with an HC participant who has the exact same age and sex, ensuring a perfectly balanced comparison between groups. Matching was performed without replacement, meaning each participant was used only once to form unique pairs. The final dataset for analysis after matching consisted of N=5 146 participants (MDD: 2 573; HC: 2 573).

**References**

1.	Schmaal, L. *et al.* Subcortical brain alterations in major depressive disorder: findings from the ENIGMA Major Depressive Disorder working group. *Mol Psychiatry* 21, 806–812 (2016).

2.	Schmaal, L. *et al.* Cortical abnormalities in adults and adolescents with major depression based on brain scans from 20 cohorts worldwide in the ENIGMA Major Depressive Disorder Working Group. *Mol Psychiatry* 22, 900–909 (2017).

3.	Fortin, J. P. *et al.* Harmonization of cortical thickness measurements across scanners and sites. *Neuroimage* 167, 104–120 (2018).

4.	Johnson, W. E., Li, C. & Rabinovic, A. Adjusting batch effects in microarray expression data using empirical Bayes methods. *Biostatistics* 8, 118–127 (2007).

5.	Radua, J. *et al.* Increased power by harmonizing structural MRI site differences with the ComBat batch adjustment method in ENIGMA. *Neuroimage* 218, (2020).
