# Supplementary material for: Decomposing neuroanatomical heterogeneity in depression: insights from an ENIGMA major depressive disorder working group study in 5146 individuals
Source: Transl Psychiatry. 2026 Jun 23;16:452. doi: 10.1038/s41398-026-04189-x (PMC13547417; doi:10.1038/s41398-026-04189-x)
Supplement: Supplementary file 2 [file 41398_2026_4189_MOESM2_ESM.docx]

**Decomposing neuroanatomical heterogeneity in depression – Supplementary Tables**

Content:

Sample preporcessing and demographics

**Supplementary Table S1.** ENIGMA – Major Depressive Disorder Working Group Summary of sample preprocessing.

**Supplementary Table S2.** ENIGMA – Major Depressive Disorder Working Group Demographics. Age (in years), sex, and number of MDD patients and HCs included per participating cohort before and after matching for age and sex.

Cohort specific exclusion criteria and image acquisition

**Supplementary Table S3.** ENIGMA – Major Depressive Disorder Working Group Instrument for diagnosing Major Depressive Disorder and exclusion criteria by participating cohort.

**Supplementary Table S4.** ENIGMA – Major Depressive Disorder Working Group Image acquisition and processing by participating cohort.

Validation of harmonization

**Supplementary Table S5.** PERMANOVA and MDMR results for site effects before and after neuroComBat harmonization in SV, CSA, and CT

Global and regional neuroanatomical heterogeneity results

**Supplementary Table S6.** ENIGMA – Major Depressive Disorder Working Group PBSI score differences between MDD patients and age-/sex-matched HCs in CT, CSA and SV.

**Supplementary Table S7.** ENIGMA – Major Depressive Disorder Working Group CVR differences between MDD patients and age-/sex-matched HCs in CT.

Moderator analyses on global and regional neuroanatomical heterogeneity results

**Supplementary Table S8.** ENIGMA – Major Depressive Disorder Working Group ANOVA of clinical and demographic variables on PBSI score differences between MDD patients and HCs in CT.

**Supplementary Table S9.** ENIGMA – Major Depressive Disorder Working Group PBSI score differences between age-stratified MDD patients and HCs in CT.

**Supplementary Table S10.** ENIGMA – Major Depressive Disorder Working Group PBSI score differences between sex-stratified MDD patients and HCs in CT.

**Supplementary Table S11.** ENIGMA – Major Depressive Disorder Working Group CVR differences between adult MDD patients and age-/sex-matched adult HCs in CT.

**Supplementary Table S12.** ENIGMA – Major Depressive Disorder Working Group CVR differences between adolescent MDD patients and age-/sex-matched adolescent HCs in CT.

**Supplementary Table S13.** ENIGMA – Major Depressive Disorder Working Group CVR differences between male MDD patients and age-matched male HCs in CT.

**Supplementary Table S14.** ENIGMA – Major Depressive Disorder Working Group CVR differences between female MDD patients and age-matched female HCs in CT.

**Supplementary Table S15.** ENIGMA – Major Depressive Disorder Working Group ANOVA of clinical characteristics within the MDD group on PBSI scores in CT.

**Supplementary Table S16.** ENIGMA – Major Depressive Disorder Working Group PBSI score differences between MDD patients using ADs, MDD patients not using ADs and age-/sex-matched HCs in CT.

**Supplementary Table S17.** ENIGMA – Major Depressive Disorder Working Group CVR differences between MDD patients using ADs and age-/sex-matched HCs in CT.

**Supplementary Table S18.** ENIGMA – Major Depressive Disorder Working Group CVR differences between MDD patients not using ADs and age-/sex-matched HCs in CT.

Patient-specific neuroanatomical variability results

**Supplementary Table S19.** ENIGMA – Major Depressive Disorder Working Group Clinical and demographic differences between *infra-norm*, *norm*, and *supra-norm* MDD patients in CT.

**Supplementary Table S20.** ENIGMA – Major Depressive Disorder Working Group Clinical and demographic differences between *infra-norm*, *norm*, and *supra-norm* MDD patients in CSA.

**Supplementary Table S21.** ENIGMA – Major Depressive Disorder Working Group Clinical and demographic differences between *infra-norm*, *norm*, and *supra-norm* MDD patients in SV.

**Supplementary Table S1.** ENIGMA – Major Depressive Disorder Working Group Summary of sample preprocessing.

| **Cohort** | | All available individuals | | Removed after preprocessing* | | Removed before matching** | |
| --- | --- | --- | --- | --- | --- | --- | --- |
|  |  | N  MDD | N  HC | N  MDD | N  HC | N  MDD | N  HC |
| 1 | AFFDIS | 29 | 20 | 2 | 1 | 0 | 0 |
| 2 | AMC | 51 | 0 | 0 | 0 | 10 | 0 |
| 3 | Barcelona | 62 | 32 | 14 | 5 | 0 | 0 |
| 4 | Basel | 63 | 55 | 0 | 6 | 44 | 0 |
| 5 | BiDirect | 577 | 435 | 16 | 8 | 85 | 0 |
| 6 | Bipolar Family Study | 244 | 564 | 7 | 9 | 237 | 0 |
| 7 | BRCDECC London | 69 | 61 | 0 | 0 | 15 | 0 |
| 8 | CLiNG | 49 | 319 | 23 | 129 | 26 | 0 |
| 9 | CSAN | 60 | 49 | 0 | 0 | 60 | 0 |
| 10 | DCHS | 24 | 75 | 13 | 30 | 11 | 0 |
| 11 | DIP | 22 | 23 | 5 | 3 | 3 | 0 |
| 12 | EPISCA | 19 | 30 | 1 | 0 | 18 | 0 |
| 13 | ETPB | 34 | 26 | 0 | 0 | 1 | 0 |
| 14 | FIDMAG | 36 | 34 | 7 | 2 | 5 | 0 |
| 15 | FOR2017 – Munster | 172 | 224 | 19 | 43 | 3 | 0 |
| 16 | FOR2107 – Marbourg | 328 | 412 | 60 | 66 | 28 | 0 |
| 17 | Hiroshima | 150 | 169 | 0 | 0 | 12 | 0 |
| 18 | Houston | 76 | 100 | 0 | 0 | 17 | 0 |
| 19 | Houston adolescents | 28 | 86 | 0 | 0 | 26 | 0 |
| 20 | Melbourne | 156 | 102 | 17 | 6 | 15 | 0 |
| 21 | Minnesota | 70 | 40 | 0 | 0 | 36 | 0 |
| 22 | MODECT | 42 | 0 | 2 | 0 | 40 | 0 |
| 23 | MOODS / DEP-ARREST CLIN | 64 | 32 | 0 | 0 | 1 | 0 |
| 24 | Moral Dilemma | 24 | 46 | 1 | 1 | 23 | 0 |
| 25 | Munster Neuroimaging Cohort | 283 | 725 | 107 | 156 | 21 | 0 |
| 26 | NESDA | 154 | 65 | 0 | 0 | 0 | 0 |
| 27 | PreNeSt | 82 | 0 | 2 | 0 | 80 | 0 |
| 28 | San Francisco UCSF | 75 | 90 | 5 | 9 | 14 | 0 |
| 29 | San Raffaele Milano OSR | 160 | 0 | 134 | 0 | 1 | 0 |
| 30 | Sexpect | 20 | 20 | 20 | 20 | 0 | 0 |
| 31 | SHIP-START | 138 | 448 | 15 | 51 | 0 | 0 |
| 32 | SHIP-Trend | 312 | 938 | 45 | 164 | 0 | 0 |
| 33 | Singapore | 23 | 17 | 1 | 1 | 0 | 0 |
| 34 | SoCAT | 79 | 100 | 0 | 0 | 30 | 0 |
| 35 | Stanford FAA | 14 | 18 | 2 | 1 | 0 | 0 |
| 36 | Stanford T1w Aggregate | 56 | 59 | 33 | 30 | 7 | 0 |
| 37 | StanfqMRI | 27 | 19 | 3 | 2 | 24 | 0 |
| 38 | Sydney | 212 | 105 | 8 | 2 | 9 | 0 |
| 39 | TAD | 42 | 0 | 1 | 0 | 6 | 0 |
| 40 | TIGER | 49 | 11 | 0 | 0 | 0 | 0 |
| 41 | TiPs | 30 | 77 | 1 | 5 | 29 | 0 |
|  | **Total** | **4205** | **5626** | **564** | **750** | **937** | **0** |
|  |  | **9831** | | **1314** | | **937** | |
| *Deduplication based on SubjID; removed if NA or other than HC/MDD in Diagnosis; NA or other than Male/Female in Sex; NA in Age; > 5% missing measures across all neuroanatomical metrics (CT, CSA, SV)  **Removed if NA in the clinical variables current AD use; Recurrence status; AO  MDD, major depressive disorder; HC, healthy control; Dx, diagnosis; AD, antidepressant; AO, age of illness onset; CT, cortical thickness; CSA, cortical surface area; SV, subcortical volume. | | | | | | | |

**Supplementary Table S2.** ENIGMA – Major Depressive Disorder Working Group Demographics. Summary of age (in years), sex, and number of MDD patients and HCs per participating cohort of the final sample included after matching for age and sex.

| **Cohort** | | Final sample after 1:1 matching* between MDD patients and HCs | | | | | |
| --- | --- | --- | --- | --- | --- | --- | --- |
|  |  | Age (Mean ± SD)  MDD | Age (Mean ± SD)  HC | % Female  MDD | % Female  HC | N  MDD | N  HC |
| 1 | AFFDIS | (38.78 ± 15.46) | (32.06 ± 12.63) | 44.44 | 50.00 | 27 | 18 |
| 2 | AMC | (29.15 ± 4.79) | NA | 100.00 | NA | 41 | 0 |
| 3 | Barcelona | (46.65 ± 7.55) | (45.88 ± 8.03) | 75.00 | 76.92 | 48 | 26 |
| 4 | Basel | (35.79 ± 8.07) | (25.73 ± 3.46) | 52.63 | 55.10 | 19 | 49 |
| 5 | BiDirect | (49.28 ± 7.22) | (48.02 ± 6.16) | 58.82 | 60.00 | 476 | 255 |
| 6 | Bipolar Family Study | NA | (57.68 ± 10.21) | NA | 63.00 | 0 | 327 |
| 7 | BRCDECC London | (48.67 ± 8.69) | (48.73 ± 6.42) | 66.67 | 72.73 | 54 | 11 |
| 8 | CliNG | NA | (23.78 ± 6.28) | NA | 0.00 | 0 | 36 |
| 9 | CSAN | NA | (32.29 ± 12.12) | NA | 75.56 | 0 | 45 |
| 10 | DCHS | NA | (31.24 ± 7.14) | NA | 100.00 | 0 | 45 |
| 11 | DIP | (45.93 ± 12.68) | (38.55 ± 10.24) | 64.29 | 90.91 | 14 | 11 |
| 12 | EPISCA | NA | (14.73 ± 1.55) | NA | 86.67 | 0 | 30 |
| 13 | ETPB | (36.09 ± 9.72) | (34.09 ± 10.64) | 60.61 | 72.73 | 33 | 22 |
| 14 | FIDMAG | (49.38 ± 12.46) | (42.82 ± 11.6) | 75.00 | 81.82 | 24 | 22 |
| 15 | FOR2017 – Munster | (35.87 ± 12.87) | (26.97 ± 9.1) | 67.33 | 77.00 | 150 | 100 |
| 16 | FOR2107 – Marbourg | (38.84 ± 13.79) | (31.93 ± 11.22) | 65.83 | 79.01 | 240 | 262 |
| 17 | Hiroshima | (44.28 ± 11.96) | (40.25 ± 7.87) | 49.28 | 83.95 | 138 | 81 |
| 18 | Houston | (39.37 ± 13.38) | (39.24 ± 10.14) | 66.10 | 85.29 | 59 | 34 |
| 19 | Houston adolescents | (11.5 ± 3.54) | (14.88 ± 1.8) | 0.00 | 48.84 | 2 | 43 |
| 20 | Melbourne | (19.2 ± 2.8) | (17.7 ± 1.67) | 54.84 | 60.66 | 124 | 61 |
| 21 | Minnesota | (15.53 ± 1.85) | (16.33 ± 1.51) | 70.59 | 63.64 | 34 | 33 |
| 22 | MODECT | NA | NA | NA | NA | NA | NA |
| 23 | MOODS / DEP-ARREST CLIN | (34.76 ± 12.32) | (36.88 ± 9.76) | 69.35 | 75.00 | 62 | 8 |
| 24 | Moral Dilemma | NA | (18.12 ± 1.49) | NA | 100.00 | 0 | 40 |
| 25 | Munster Neuroimaging Cohort | (39.6 ± 12.21) | (40.36 ± 10.21) | 60.28 | 86.84 | 141 | 76 |
| 26 | NESDA | (36.6 ± 10.69) | (43.09 ± 5.26) | 63.83 | 100.00 | 141 | 11 |
| 27 | PreNeSt | NA | NA | NA | NA | NA | NA |
| 28 | San Francisco UCSF | (15.48 ± 1.39) | (15.76 ± 0.96) | 67.86 | 66.00 | 56 | 50 |
| 29 | San Raffaele Milano OSR | (51.46 ± 14.41) | NA | 70.83 | NA | 24 | 0 |
| 30 | Sexpect | NA | NA | NA | NA | NA | NA |
| 31 | SHIP-START | (52.99 ± 10.96) | (50.11 ± 10.55) | 72.73 | 54.90 | 121 | 286 |
| 32 | SHIP-Trend | (49.11 ± 11.48) | (44.17 ± 10.39) | 67.42 | 49.91 | 267 | 563 |
| 33 | Singapore | (39.74 ± 8.12) | (44 ± 9.9) | 36.84 | 100.00 | 19 | 2 |
| 34 | SoCAT | (35.38 ± 14.38) | (40.46 ± 11.18) | 84.44 | 100.00 | 45 | 13 |
| 35 | Stanford FAA | NA | NA | NA | NA | NA | NA |
| 36 | Stanford T1w Aggregate | NA | NA | NA | NA | NA | NA |
| 37 | StanfqMRI | NA | (30 ± 15.56) | NA | 100.00 | 0 | 2 |
| 38 | Sydney | (36.31 ± 22.03) | (42 ± 28.91) | 69.73 | 75.00 | 185 | 4 |
| 39 | TAD | NA | NA | NA | NA | NA | NA |
| 40 | TIGER | (15.79 ± 1.42) | (15.17 ± 0.75) | 55.17 | 66.67 | 29 | 6 |
| 41 | TiPs | NA | (43 ± NA) | NA | 100.00 | 0 | 1 |
|  | Total | (40.57 ± 15.13) | (40.57 ± 15.13) | 64.17 | 64.17 | 2573 | 2573 |
| *1:1 matching between MDD patients and HCs based on age and sex across cohorts  MDD, major depressive disorder; HC, healthy control; SD, standard deviation. | | | | | | | |

**Supplementary Table S3.** ENIGMA – Major Depressive Disorder Working Group Instrument for diagnosing Major Depressive Disorder and exclusion criteria by participating cohort.

| Cohort | | Country | Diagnosis instrument | Exclusion criteria |
| --- | --- | --- | --- | --- |
| 1 | AFFDIS | Germany | MDD diagnosis criteria according to ICD-10, DSM-IV | All subjects exclusion criteria: current or history of neurological disorder or brain injury, current substance abuse or dependence (not including nicotine), pregnancy, MRI contraindications, inability to give consent. MDD specific: Comorbid psychiatric diagnosis. Healthy control specific: current or history of psychiatric diagnosis. |
| 2 | AMC | Netherlands | M.I.N.I. Plus neuropsychiatric interview | Less than three-week medication-free interval before scanning, current psychotropic medication use, a history of chronic or neurological disorder, family history of sudden heart failure or epileptic attacks, pregnancy (tested via urine sampling prior to the assessment), breast feeding, alcohol dependence and contra-indications for an MRI scan (e.g., ferromagnetic fragments). Participants agreed to abstain from smoking, caffeine and alcohol use for 24 hours prior to the assessments. |
| 3 | Barcelona | Spain | MDD-TR diagnosis criteria according to DSM-IV, CIDI-interview and HDRS | The exclusion criteria for healthy participants were. Lifetime psychiatric diagnoses, first-degree relatives with psychiatric diagnoses and clinically significant physical or neurological illnesses. Axis I comorbidity according to DSM-IV-TR criteria was an exclusion criteria for all participants. |
| 4 | Basel | Switzerland | HDRS, BDI, SHAPS | Exclusion criteria patients: Presence of additional primary psychiatric disorders other than MDD, acute suicidal tendencies, history of electroconvulsive therapy or ketamine treatment within the past 6 months, pregnant or lactating women |
| 5 | BiDirect | Germany | MDD diagnosis criteria according to ICD-10, M.I.N.I. neuropsychiatric interview, IDS, HDRS, CESD | Dementia, addiction |
| 6 | Bipolar Family Study | Scotland | SCID interview | MDD subjects: presence of other axis I diagnoses. Control subjects: no medical history, including neurological and psychiatric history, as well as no previous or actual use of psychotropic medication. All subjects: any major neurological disorder, learning disability, or any history of head injury that included loss of consciousness and any contraindications to MRI. |
| 7 | BRCDECC London | England | SCAN interview | Contraindications to MRI, diagnosis of neurological disorder, head injury leading to loss of consciousness or conditions known to affect brain structure or function (including alcohol or substance misuse), if they or a first-degree relative had ever fulfilled criteria for mania, hypomania, schizophrenia or mood-incongruent psychosis. |
| 8 | CliNG | Germany | ICD-10 interview | MDD subjects: past or actual presence of other axis I diagnoses other than anxiety disorders, alcohol/cannabis abuse and tobacco dependence; neurological or other medical conditions that could be related to affective symptoms. Control subjects: no medical history, including neurological and psychiatric history, as well as no previous or actual use of psychotropic medication |
| 9 | CSAN | USA | M.I.N.I. neuropsychiatric interview | Current MDD: a current DSM-5 diagnosis of substance use disorder, except nicotine; a psychotic disorder, except depression with mood-congruent psychotic features; new antidepressant medication during the month before study participation (two months for fluoxetine); change of the dose of psychotropic medications over the last month (antidepressant and antipsychotic medication) or the last two months (mood stabilizers and anticonvulsants). |
| 10 | DCHS | South Africa | M.I.N.I. neuropsychiatric interview | In the DCHS MRI substudy mothers were excluded for: loss of consciousness longer than 30 minutes; inability to speak English; current/lifetime alcohol and/or substance dependence or abuse; psychopathology other than PTSD and/or MDD; traumatic brain injury; standard MRI exclusion criteria. |
| 11 | DIP | Netherlands | M.I.N.I. SCAN neuropsychiatric interview | Exclusion MDD: Comorbid axis-I disorders other than anxiety disorders or past substance abuse, other psychotropic medication than stable use of SSRI/SNRI/TCA, established cardiovascular disease, active and concrete suicidal plans, inadequate language proficiency, cognitive impairments or neurological disease that interferes with task performance. Exclusion CTL: Same as MDD, lifetime history of MDD, BDI>8. |
| 12 | EPISCA | Netherlands | ADIS | Primary DSM-IV clinical diagnosis of ADHD, ODD, CD, pervasive developmental disorders, post-traumatic stress disorder, Tourette’s syndrome, obsessive–compulsive disorder, bipolar disorder, and psychotic disorders; current substance abuse; history of neurological disorders or severe head injury; age < 12 or > 21 years; pregnancy; left-handedness; IQ score < 80 as measured by the Wechsler Intelligence Scale for Children (WISC) (Wechsler, 1991) or Adults (Wechsler, 1997); and general MRI contra- indications. |
| 13 | ETPB | USA | HDRS, BDI, SHAPS, MADRS | Current or past diagnosis of Schizophrenia or any other psychotic disorder as defined in the DSM-IV. Subjects with a history of DSM-IV drug or alcohol dependency or abuse (except for nicotine or caffeine) within the preceding 3 months. Female subjects who are either pregnant or nursing. Serious, unstable illnesses including hepatic, renal, gastroenterologic, respiratory, cardiovascular (including ischemic heart disease), endocrinologic, neurologic, immunologic, or hematologic disease. Subjects with uncorrected hypothyroidism or hyperthyroidism. Subjects with one or more seizures without a clear and resolved etiology. Treatment with a reversible MAOI within 4 weeks prior to study phase I. Treatment with fluoxetine within 5 weeks prior to study phase I. Treatment with any other concomitant medication not allowed. |
| 14 | FIDMAG | Spain | MDD-TR diagnosis criteria according to DSM-IV | Patients were excluded (i) if they were left-handed; (ii) if they were younger than 18 or older than 65 years; (iii) if they had a history of brain trauma or neurological disease; (iv) if they had shown alcohol/ substance abuse within 12 months prior to participation; and (v) if they had undergone electroconvulsive therapy in the previous 12 months. |
| 15 | FOR2017 – Munster | Germany | SCID-1 interview | Exclusion criteria all: any MRI contraindications; any neurological abnormalities. Exclusion criteria controls: any current or former psychiatric disorder; Exclusion criteria patients: substance dependence or current benzodiazepine treatment (wash out of at least three half-lives before study participation)” |
| 16 | FOR2107 – Marbourg | Germany | SCID-1 interview | Exclusion criteria all: any MRI contraindications; any neurological abnormalities. Exclusion criteria controls: any current or former psychiatric disorder; Exclusion criteria patients: substance dependence or current benzodiazepine treatment (wash out of at least three half-lives before study participation)" |
| 17 | Hiroshima | Japan | M.I.N.I. neuropsychiatric interview | MDD patients: comorbid psychiatric disorders other than MDD, Control subjects: any history of psychiatric disorder. |
| 18 | Houston | USA | SCID interview | MDD subjects: head trauma with residual effects, neurological disorders, uncontrolled major medical conditions based on patient self reports and current drug abuse. In addition, Healthy controls (HC) were excluded if they had a history of any Axis I disorder or had a first-degree relative with any Axis I disorder. |
| 19 | Houston adolescents | USA | MDD diagnosis criteria according to DSM-IV | MDD subjects: age below 18; lifetime or current diagnosis of psychotic disorder, or bipolar I or II disorder; substance abuse/dependence in 6 months prior to study inclusion; current major medical problems. Control subjects: age below 18; current major medical problems; current psychiatric or neurologic disorder; history of psychiatric disorders in a first-degree relative; current major medical problems. Both groups: MRI contra-indications. |
| 20 | Melbourne | Australia | SCID interview | MDD subjects: lifetime or current SCID-I diagnosis of psychotic disorder, or bipolar I or II disorder. Control subjects: any SCID-I diagnosis or medication use. Both groups: Acute or unstable medical disorder; general MRI contraindications. |
| 21 | Minnesota | USA | KSADS Present and Lifetime Version, CDRS-R | Exclusion criteria for both groups included the presence of a neurologic or other chronic medical condition, mental retardation, pervasive developmental disorder, substance use disorder, bipolar disorder, or schizophrenia. |
| 22 | MODECT | Netherlands | M.I.N.I. neuropsychiatric interview | Exclusion criteria were another major DSM-IV-TR diagnosis, such as schizophrenia, bipolar or schizoaffective disorder and a history of major neurological illness (including Parkinson’s disease, stroke and dementia). |
| 23 | MOODS / DEP-ARREST CLIN | France | MDD diagnosis criteria according to DSM-V, M.I.N.I. neuropsychiatric interview | Patients suffering from bipolar disorder, psychotic disorder, eating disorder, and addictions, according to the DSM-5 criteria, or from nasal polyposis, chronic or acute sinusitis, chronic or acute rhinitis or pregnancy or breastfeeding, were not included. HCs were included based on the absence of current or past mental disorders or somatic conditions, particularly nasal polyposis and chronic or acute sinusitis or rhinitis. |
| 24 | Moral Dilemma | Australia | SCID interview | MDD subjects: lifetime or current SCID-I diagnosis of psychotic disorder, or bipolar I or II disorder; current antidepressant medication use. Control subjects: any SCID-I diagnosis or medication use. Both groups: Acute or unstable medical disorder; general MRI contraindications. |
| 25 | Munster Neuroimaging Cohort | Germany | SCID interview | MDD subjects: presence of bipolar disorder, schizoaffective disorders and schizophrenia; substance-related disorders or current benzodiazepine treatment (wash out of at least three half-lives before study participation), and former electroconvulsive therapy. Control subjects: any current or former psychiatric disorder. Both groups: any neurological abnormalities, MRI contra-indications. |
| 26 | NESDA | Netherlands | CIDI interview | MDD subjects: presence of axis-I disorders other than MDD, panic disorder, social anxiety disorder, or generalized anxiety disorder and any use of psychotropic medication other than stable use of SSRIs or infrequent benzodiazepine use (i.e., equivalent to 2 doses of 10 mg of oxazepam 3 times per week or use within 48 hours prior to scanning). Control subjects: no Axis-I diagnosis, no medication use. All subjects: presence or history of major internal or neurological disorder, dependence on or recent abuse (past year) of alcohol and/or drugs, hypertension, and general MRI contraindications. |
| 27 | PreNeSt | Germany | SCID interview | Exclusion criteria were epilepsy or other neurological diseases, pregnancy, metallic implants, claustrophobia, as contraindications to MRI or rTMS, or, in case of regular psychopharmacological medication intake, change of type or dosage in the 2 weeks prior to participation or during the course of the study. |
| 28 | San Francisco UCSF | USA | KSADS (semi-structured interview based on DSM) for MDD, DISC/DPS for Controls | Exclusion criteria for all participants included: 1) use of pharmacotherapeutics for treating psychiatric conditions within the past 6 months, 2) misuse of drugs within two months prior to MRI scanning; 3) two or more alcoholic drinks per week within the previous month (as assessed by the Customary Drinking and Drug Use Record; CDDR); 4) a full scale IQ score of less than 75 (as assessed by the Wechsler Abbreviated Scale of Intelligence; WASI); 5) contraindications for MRI including ferromagnetic implants and claustrophobia; 6) pregnancy or the possibility of pregnancy; 7) left-handedness; 8) prepubertal status (as assessed as Tanner stages of 1 or 2); 9) inability to understand and comply with procedures; 10) neurological disorder (including meningitis, migraine, or HIV); 11) head trauma; 12) learning disability; 13) serious health problems; and 14) complicated or premature birth (i.e., birth before 33 weeks of gestation). The MDD group was subject to the additional exclusion criterion of a primary psychiatric diagnosis other than MDD. The HCL group was subject to the additional exclusion criteria of: 1) history of mood or psychotic disorders in a first- or second-degree relative (as assessed by the Family Interview for Genetics; FIGS); and 2) current or lifetime DSM-IV-TR Axis I psychiatric disorder. |
| 29 | San Raffaele Milano OSR | Italy | SCID interview | Other diagnoses on Axis I; pregnancy, history of epilepsy, major medical and neurological disorders; absence of a history of drug or alcohol dependency or abuse within the last six months. inflammation-related symptoms, including fever and infectious or inflammatory disease; uncontrolled systemic disease; uncontrolled metabolic disease or other significant uncontrolled somatic disorder known to affect mood; somatic medications known to affect mood or the immune system, such as corticosteroids, non-steroid anti-inflammatory drugs and statins. |
| 30 | Sexpect | Germany | ICD-10 interview | MDD subjects: history of seizures, medication with glutamate modulating drugs (ketamine, riluzole, etc.) or benzodiazepines, prior electroconvulsive therapy (ECT) treatments and pregnancy, atypical forms of depression, any additional psychiatric disorder, and a history of substance abuse or dependence. Control subjects: psychiatric illness. Both groups: contraindications against MRI, major medical and neurological illness. |
| 31 | SHIP-START | Germany | M-CIDI interview | MDD subjects: presence of axis-I disorders other than MDD, anxiety disorders, conversion, somatization and eating disorder. Control subjects: no lifetime diagnosis of depression, no antidepressiva, and severity index=0 All subjects: We removed subjects with medical conditions (e.g. a history of cerebral tumor, stroke, Parkinson’s diseases, multiple sclerosis, epilepsy, hydrocephalus, enlarged ventricles, pathological lesions) or due to technical reasons (e.g. severe movement artifacts or inhomogeneity of the magnetic field). |
| 32 | SHIP-Trend | Germany | M-CIDI interview | MDD subjects: presence of axis-I disorders other than MDD, anxiety disorders, conversion, somatization and eating disorder. Control subjects: no lifetime diagnosis of depression, no antidepressiva, and severity index=0 All subjects: We removed subjects with medical conditions (e.g. a history of cerebral tumor, stroke, Parkinson’s diseases, multiple sclerosis, epilepsy, hydrocephalus, enlarged ventricles, pathological lesions) or due to technical reasons (e.g. severe movement artifacts or inhomogeneity of the magnetic field). |
| 33 | Singapore | Singapore | SCID interview | Exclusion criteria 1) History of significant head injury 2) Neurological diseases such as epilepsy, cerebrovascular accident 3) Impaired thyroid function 4) Steroid use 5) DSM IV alcohol or substance use or dependence 6) Contraindications to MRI (e.g. pacemaker, orbital foreign body, recent surgery/procedure with metallic devices/implants deployed) using standard MRI Request Form from NNI 7) Pregnant women 8) Claustrophobia. |
| 34 | SoCAT | Turkey | SCID interview | Exclusion criteria 1) History of significant head injury 2) Neurological diseases such as epilepsy, cerebrovascular accident 3) Other diagnoses on Axis I disorders4). |
| 35 | Stanford FAA | USA | SCID interview | MDD subjects: presence of axis-I disorders other than MDD, anxiety and eating disorders. Control subjects: control individuals did not meet diagnostic criteria for any current psychiatric. Both groups: alcohol / substance abuse or dependence within six months prior to MRI scanning, history of head trauma with loss of consciousness > 5 min, aneurysm, or any neurological or metabolic disorders that require ongoing medication or that may affect the central nervous system (including thyroid disease, diabetes, epilepsy or other seizures, or multiple sclerosis), MRI contraindications, or bad MRI data (e.g., extreme movement). |
| 36 | Stanford T1w Aggregate | USA | SCID interview | MDD subjects: presence of axis-I disorders other than MDD, anxiety and eating disorders. Control subjects: control individuals did not meet diagnostic criteria for any current psychiatric. Both groups: alcohol / substance abuse or dependence within six months prior to MRI scanning, history of head trauma with loss of consciousness > 5 min, aneurysm, or any neurological or metabolic disorders that require ongoing medication or that may affect the central nervous system (including thyroid disease, diabetes, epilepsy or other seizures, or multiple sclerosis), MRI contraindications, or bad MRI data (e.g., extreme movement). |
| 37 | StanfqMRI | USA | SCID interview | MDD subjects: presence of axis-I disorders other than MDD, anxiety and eating disorders . Control subjects: control individuals did not meet diagnostic criteria for any current psychiatric. Both groups: alcohol / substance abuse or dependence within six months prior to MRI scanning, history of head trauma with loss of consciousness > 5 min, aneurysm, or any neurological or metabolic disorders that require ongoing medication or that may affect the central nervous system (including thyroid disease, diabetes, epilepsy or other seizures, or multiple sclerosis), MRI contraindications, or bad MRI data (e.g., extreme movement). |
| 38 | Sydney | Australia | SCID interview | MDD subjects: presence of axis-I disorders other than MDD, panic disorder, social anxiety disorder, or generalized anxiety disorder. Control subjects: no Axis-I diagnosis, no medication use. Exclusion criteria for all subjects included medical instability (as determined by a psychiatrist), history of neurological disease (e.g. tumour, head trauma, epilepsy), medical illness known to impact cognitive and brain function (e.g. cancer), intellectual and/or developmental disability and insufficient English for neuropsychological assessment. All subjects were asked to abstain from drug or alcohol use for 48 hours prior to testing and informed about a drug screen protocol. |
| 39 | TAD | USA | KSADS | All subjects: Exclusion criteria were premenarchal status (for females), history of concussion within the past 6 weeks or history of any lifetime concussion with loss of consciousness, contraindications to MRI scanning (e.g. braces, metal implants, or claustrophobia), serious neurological or intellectual disorders that could interfere with the participant's ability to complete study components. MDD subjects: meeting lifetime or current DSM-IV criteria for any Bipolar Disorder, Psychosis, or Alcohol Dependence, or DSM-5 criteria for Moderate Substance Use Disorder with substance-specific threshold for withdrawal. Control subjects: any current or past DSM-IV Axis I Disorder and first-degree relative with confirmed or suspected history of depression, mania, psychosis, or substance dependence. |
| 40 | TIGER | USA | KSADS | All subjects: Exclusion criteria were premenarchal status (for females), history of concussion within the past 6 weeks or history of any lifetime concussion with loss of consciousness, contraindications to MRI scanning (e.g. braces, metal implants, or claustrophobia), serious neurological or intellectual disorders that could interfere with the participant's ability to complete study components. MDD subjects: meeting lifetime or current DSM-IV criteria for any Bipolar Disorder, Psychosis, or Alcohol Dependence, or DSM-5 criteria for Moderate Substance Use Disorder with substance-specific threshold for withdrawal. Control subjects: any current or past DSM-IV Axis I Disorder and first-degree relative with confirmed or suspected history of depression, mania, psychosis, or substance dependence. |
| 41 | TiPs | Germany | SCID interview | MDD subjects: presence of axis-I disorders other than MDD or adjustment disorders. Control subjects: no Axis-I diagnosis, no medication use. Exclusion criteria for all subjects included history of neurological disease (e.g. tumour, head trauma, epilepsy) or untreated internal medical condtitions, intellectual and/or developmental disability. Only German native speakers were allowed to participate. |
| ADIS: Anxiety Disorders Interview Schedule; BDI: Beck Depression Inventory; CDRS-R: Children's Depression Rating Scale-Revised; CESD: Center for Epidemiologic Studies Depression Scale; CIDI: Composite International Diagnostic Interview; DISC/DPS: Diagnostic Interview Schedule for Children / Depression Prevention Study; DSM-IV / -V: Diagnostic and Statistical Manual of Mental Disorders, 4th / 5th Edition; FIGS: Family Interview for Genetics; HDRS: Hamilton Depression Rating Scale; ICD-10: International Classification of Diseases, 10th Revision; IDS: Inventory of Depressive Symptomatology; KSADS: Schedule for Affective Disorders and Schizophrenia for School-Age Children; MADRS: Montgomery–Åsberg Depression Rating Scale; M-CIDI: Munich Composite International Diagnostic Interview; MDD: Major Depressive Disorder; MDD-TR: Major Depressive Disorder - Treatment-Resistant; M.I.N.I.: Mini International Neuropsychiatric Interview; M.I.N.I. Plus: Mini International Neuropsychiatric Interview, Extended Version; SCAN: Schedules for Clinical Assessment in Neuropsychiatry; SCID: Structured Clinical Interview for DSM Disorders; SCID-1: Structured Clinical Interview for DSM-IV Axis I Disorders; SHAPS: Snaith-Hamilton Pleasure Scale; WASI: Wechsler Abbreviated Scale of Intelligence; WISC: Wechsler Intelligence Scale for Children. | | | | |

**Supplementary Table S4.** ENIGMA – Major Depressive Disorder Working Group Image acquisition and processing by participating cohort.

| **Cohort** | | Scanner type | Sequence T1 | FreeSurfer version | Slice orientation | Operating system |
| --- | --- | --- | --- | --- | --- | --- |
| 1 | AFFDIS | 3T Siemens Magnetom TrioTim | 3D T1 (176 slices; TR = 2250 ms; TE = 3.26 ms; FOV 256; voxel size 1X1X1mm) | 5.3 | Sagittal | Linux CentOS |
| 2 | AMC | 3T Philips | T1 sequence details: 3D-TFE sequence TR= 9.7 ms, TE=4.6ms, matrix 192x192, voxel size = 0.875 x 0.875 x 1.2 mm; 120 slices. Axial plane. Philips 3T Ingenia 16 channel coil | 5.3 | Transverse | freesurfer-Linux-centos6_x86_64-stable-pub-v5.3.0 |
| 3 | Barcelona | 3T Philips Achieva | 3D MPRAGE images (Whole-brain T1-weighted); TR=6.7ms, TE=3.2ms; 170 slices, voxel size 0.89X0.89X1.2 mm. Image dimensions 288X288X170; field of view: 256X256X204; slice thickness: 1.2 mm; with a sagittal slice orientation, T1 contrast enhancement, flip angle: 8º, grey matter as a reference tissue, ACQ matrix MXP = 256X240 and turbo-field echo shots (TFE) = 218. | 6 | Sagittal | Scientific Linux 5 |
| 4 | Basel | 3T Siemens Magnetom Prisma | 3D T1-weighted MPRAGE (TR = 2000 ms, TE = 3.37 ms, FOV 256 × 256 mm, voxel size = 1 × 1 × 1 mm, flip angle = 8°) | 7.2 | Sagittal | Ubuntu 20.04.6  LTS |
| 5 | BiDirect | 3T Philips Intera scanner | 3D T1-weighted turbo field echo images were collected with a the following parameters: TR = 7.26, TE = 3.56, 9° flip angle, 160 sagittal slices, matrix dimension 256 x 256, FOV = 256 x 256mm, 2mm slice thickness (reconstructed to 1mm) and a resulting voxel size of 1x1x1mm | 6 | Sagittal | freesurfer-Linux-centos6_x86_64-dev-20161222-499fc91 |
| 6 | Bipolar Family Study | 1.5T GE Signa | T1-weighted sequence. TR=500 msec; TE=4 msec; flip angle 8°; matrix 192 x 192; 180 slices; voxel size 1.25 mm x 1.25 mm x 1.20 mm; FOV=24, phase FOV 1 | 5.3 | Coronal | linux 6, x86_64, kernel 2.6.32 |
| 7 | BRCDECC London | 1.5T GE Signa HDx | ADNI-1 MPRAGE pulse sequence (details at http://adni.loni.ucla.edu/research/protocols/mri-protocols/) | 5.3 | Sagittal | Linux-centos4_x86_64 |
| 8 | CliNG | 3T Siemens Tim Trio | Standard 3D T1-weighted turbo fast low angle shot (turbo FLASH); voxel size 1 mm x 1 mm x 1mm (based on the ADNI protocol; TR=225 msec; TE=3.26 msec, FOV=256 x 256 x 192 | 5.3 | Sagittal | Linux |
| 9 | CSAN | 3T Siemens MAGNETOM PRISMA | Whole-head t1-weighted MPRAGE (TR = 2300 ms, TE = 2.34 ms, FOV 250 × 250 mm, voxel size = 0.9 × 0.868 × 0.868 mm, flip angle = 8°) | 7.2 | Sagittal | Ubuntu |
| 10 | DCHS | 3T Siemens Skyra | 3D multi-echo MPRAGE, voxel size 1 mm x 1mm x 1.5mm, TR = 2530 ms, TE = 1.69 x 3.55 x 5.41 x 7.27ms, FOV: 256x256mm, flip angle = 7° | 5.3 | Sagittal | Linux-centos6_x86_64 |
| 11 | DIP | 3T Philips | 3D T1-weighted scan (170 slices; TR = 9ms; TE = 3.6ms; 256x231 matrix of 1×1×1 mm voxels) | 5.3 | Sagittal | SUSE Linux X86_64 |
| 12 | EPISCA | 3T Philips Achieva | a sagittal 3-dimensional gradient-echo T1-weighted image was acquired (repetition time = 9.8 ms; echo time = 4.6 ms; flip angle = 8°; 140 sagittal slices; no slice gap; field of view =256 × 256 mm; 1.17 × 1.17 × 1.2 mm voxels; duration = 4:56 min) | 5.3 | Sagittal | Ubuntu 14.04.5 LTS (Linux 3.13.0-153-generic x86_64) |
| 13 | ETPB | 3T GE HDx | Fast spoiled gradient recalled echo (FSPGR). Slice Thickness: 1. Repetition Time: 8.836. Echo Time: 3.496. Inversion Time: 450. Magnetic Field Strength: 3. Spacing Between Slices: 1. Echo Train Length: 1. Percent Sampling: 100. Percent Phase Field of View: 100. Pixel Bandwidth: 195.312. Reconstruction Diameter: 256. Acquisition Matrix: 0,256,256,0. In-plane Phase Encoding Direction: ROW. Flip Angle: 13 | 5.3 | Sagittal | Linux |
| 14 | FIDMAG | 1.5T GE Signa | 3D T1: matrix size = 512 × 512, 180 contiguous axial slices, voxel resolution = 0.47 × 0.47 × 1mm, no slice gap, TE = 3.93ms, TR = 2000ms and inversion time (TI) = 710ms, flip angle = 15 degrees | 6 | Axial | Linux-centos6_x86_64 |
| 15 | FOR2017 – Munster | 3T Siemens Magnetom TiroTim | Sequence: 3D T1-weighted magnetization prepared rapid acquisition gradient echo (MPRAGE) - Sagittal Acquisition Direction, # of Slices 176, 0.5mm Slice Gap, 1.0x1.0x1.0 Voxel Size (mm3), TI 900 ms, TE 2.26 ms, TR 1900 ms, Flip Angle 9. | 5.3 | Sagittal | Red Hat Enterprise Linux Server release 5.11 (Tikanga) |
| 16 | FOR2107 – Marbourg | 3T Siemens PRISMA | Sequence: 3D T1-weighted magnetization prepared rapid acquisition gradient echo (MPRAGE). - Sagittal Acquisition Direction, # of Slices 192, 0mm Slice Gap, 1.0x1.0x1.0 Voxel Size (mm3), TI 900 ms, TE 2.28 ms, TR 1900 ms, Flip Angle 8 | 5.3 | Sagittal | Red Hat Enterprise Linux Server release 5.11 (Tikanga) |
| 17 | Hiroshima | 3T GE Signa HDxt (Sites 1,2)  3T Siemens MAGNETOM (Sites 3,4) | T1 256x256x256 matrix of 1x1x1mm voxels (Siemens: ADNI MPRAGE (tfl), GRAPPA, 192 slices, GE: SPGR, 184 slices) *Detailed scanning parameter sheets are available for all 4 scanners on request. | 5.3 | Sagittal | Linux_Ubuntu_18.04 |
| 18 | Houston | 1.5T Philips Medical Systems Gyroscan Intera (IDs in 20000s)  3T Siemens Allegra (IDs in 30000s) | Subjects in the 20000s: Fast field echo sequence- repetition time (TR) = 24 ms, echo time (TE) = 4.99 ms, flip angle = 40°, slice thickness = 1 mm, matrix size = 256 × 256 and 150 slices. Subjects in 30000s: MPRAGE- repetition time (TR) = 1750 ms, echo time (TE) = 4.39 ms, flip angle = 8°, slice thickness = 1 mm, matrix size = 208 × 256 and 160 slices. | 5.3 | Sagittal (IDs in 20000s) Transverse (IDs in 30000s) | Fedora 19 |
| 19 | Houston adolescents | 1.5T Philips Medical Systems Gyroscan Intera (IDs in 20000s)  3T Siemens Allegra (IDs in 30000s) | Subjects in the 20000s: Fast field echo sequence- repetition time (TR) = 24 ms, echo time (TE) = 4.99 ms, flip angle = 40°, slice thickness = 1 mm, matrix size = 256 × 256 and 150 slices. Subjects in 30000s: MPRAGE- repetition time (TR) = 1750 ms, echo time (TE) = 4.39 ms, flip angle = 8°, slice thickness = 1 mm, matrix size = 208 × 256 and 160 slices. | 5.3 | Sagittal (IDs in 20000s) Transverse (IDs in 30000s) | Fedora 19 |
| 20 | Melbourne | 3T GE Signa Excite | 3D BRAVO sequence 140; TR=7900 ms; TE=3000 ms; flip angle=13º; FOV=256 mm; matrix=256 x 256 | 5.3 | Axial | Linux Debian x86 64 |
| 21 | Minnesota | 3T Siemens Tim Trio | A 5-minute structural scan was acquired using a T1-weighted, high-resolution, magnetization-prepared gradient-echo sequence: repetition time, 2530 milliseconds; echo time, 3.65 milliseconds; inversion time, 1100 milliseconds; flip angle, 7°; field of view, 256 × 176 mm; voxel size, 1-mm isotropic; 224 slices; and generalized, autocalibrating, partially parallel acquisition acceleration factor, 2. | 5.3 | Coronal | Linux |
| 22 | MODECT | 3T GE Signa HDxt | T1-weigthed dataset was acquired (flip angle=12°, repetition time=7.84 milliseconds, echo time=3.02 milliseconds; matrix 256x256, voxel size 0.94x0.94x1 mm; 180 slices). | 5.3 | Coronal | Linux |
| 23 | MOODS / DEP-ARREST CLIN | 3T Philips Achieva | 3D T1-weighted image: TR=7, TE=3.5, FOV=352x352x180, Flip angle=8 degrees, number of slices: 180 slices, Slice gap 1 mm, voxel size: 0.8x0.8x1 | 6 | Transverse (Axial) | CentOS Linux 7 |
| 24 | Moral Dilemma | 3T GE Signa Excite | 3D BRAVO sequence: 140 contiguous slices; repetition time, 7900 ms; echo time, 3000 ms; flip angle, 13°; in a 25.6-cm field of view, with a 256 × 256 pixel matrix and a slice thickness of 1 mm (1 mm gap). | 5.3 | Axial | Linux Debian x86 64 |
| 25 | Munster Neuroimaging Cohort | 3T Philips Gyroscan Intera | 3D fast gradient echo sequence (turbo field echo), repetition time = 7.4 milliseconds, echo time = 3.4 milliseconds, flip angle = 9°, two signal averages, inversion prepulse every 814.5 milliseconds, acquired over a field of view of 256 (feet -head [FH]) × 204 (anterior -posterior [AP]) × 160 (right -left [RL]) mm, phase encoding in AP and RL direction, reconstructed to cubic voxels of .5 mm × .5 mm × .5 mm | 5.3 | Sagittal | Red Hat Enterprise Linux Server release 5.11 (Tikanga) |
| 26 | NESDA | 3T Phillips Achieva/Intera | 3D gradient-echo T1-weighted sequence. TR=9 msec; TE=3.5 msec; flip angle 8º, FOV = 256 mm; matrix: 25x62x56; in plane voxel size = 1 mm × 1 mm x 1 mm; 170 slices. | 5 | Sagittal | SHARK HPC, Linux environment |
| 27 | PreNeSt | 3T Siemens Magnetom Prisma | 3D MPRAGE whole-brain T1 (176 slices; TR = 2250 ms; TE = 3.3 ms; flip angle=9°; FOV 256; voxel size 1 x 1 x 1mm) | 6.0.1 and 7.3.2 | Sagittal | Linux CentOS |
| 28 | San Francisco UCSF | 3T GE Discovery MR750 | SPGR T1-weighted: TR=8.1 ms; TE=3.17 ms; TI=450 ms; flip angle=12°; 256x256 matrix; FOV=250x250 mm; 168 sagittal slices; slice thickness=1 mm; in-plane resolution=0.98x 0.98 mm | 5.3 | Sagittal | Linux-centos6_x86_64-stable-pub-v5.3.0. |
| 29 | San Raffaele Milano OSR | 3T Philips Ingenia  3T Philips Intera scanner | 3D-MPRAGE sequence: TR 2500 ms, TE 4.6 ms, field of view FOV = 230 mm, matrix = 256 × 256, in-plane resolution 0.9 × 0.9 mm, yielding 220 transversal slices with a thickness of 0.8 mm. | 5.3 | Axial | Linux Ubuntu 16.04 |
| 30 | Sexpect | 3T Siemens MAGNETOM Trio | High resolution T1 -weighted structural MRI scans of the brain were acquired for structural reference using a 3D -MPRAGE sequence (TE = 4.77 ms, TR = 2500 ms, T1 = 1100 ms, flip angle = 7°, bandwidth = 140 Hz/pixel, acquisition matrix = 256 × 256 × 192, isometric voxel size = 1.0 mm3). | 5.3 | Sagittal | Oracle Linux Server_x86_64 |
| 31 | SHIP-START | 1.5T Siemens Avanto | 3D T1-weighted (MP-RAGE/ axial plane); TR=1900 msec; TE=3.4 msec; Flip angle=15°; voxel size 1 mm x 1 mm x 1 mm | 5.3 (CT, CSA)  5.1 (SV) | Axial | Centos6_x86_64 |
| 32 | SHIP-Trend | 1.5T Siemens Avanto | 3D T1-weighted (MP-RAGE/ axial plane); TR=1900 msec; TE=3.4 msec; Flip angle=15°; voxel size 1 mm x 1 mm x 1 mm | 5.3 (CT, CSA)  5.1 (SV) | Axial | Centos6_x86_64 |
| 33 | Singapore | 3T Philips Achieva | Whole brain high resolution 3D MP-RAGE (magnetisation-prepared rapid acquisition with a gradient echo) volumetric scans (TR/TE/TI/flip angle 8.4/3.8/3000/8; matrix 256x204; FOV 240mm2) with axial orientation (reformatted to coronal) | 5.3 | Axial | Linux_Ubuntu12.04_6 4 |
| 34 | SoCAT | 3T Siemens Verio Numaris/4 Syngo MR B17 | 3D T1 weighted MP-Rage/axial plane; TR=1900 msec; TE=3.4 msec; Flip angle=15°; Voxel size 1 mm x 1 mm x 1 mm | 7.1.1 | Axial | Ubuntu 18.04 LTS |
| 35 | Stanford FAA | 3T GE Discovery MR750 | Whole-brain T1-weighted images were collected using a spoiled gradient echo (SPGR) pulse sequence (186 sagittal slices; resolution = 0.9 mm isotropic; flip angle = 12°; repetition time [TR] = 6,240 ms; echo time [TE] = 2.34 ms) | 5.3 | Sagittal | Centos6_x86_64, Linux-based HPC |
| 36 | Stanford T1w Aggregate | 1.5T GE Signa Excite | Whole-brain T1-weighted images were collected using a spoiled gradient echo (SPGR) pulse sequence (116 sagittal slices; through-plane resolution = 1.5 mm; in-plane resolution = 0.86 x 0.86 mm; flip angle = 15 degrees; repetition time [TR] = 8.3-10.1 ms; echo time [TE] = 1.7-3.0; inversion time [TI] = 300 ms; matrix = 256 x 192). | 5.3 | Sagittal | Linux-centos6_x86_64 |
| 37 | StanfqMRI | 3T GE Discovery MR750 | Whole-brain T1-weighted images were collected using two protocols: for qMRI a spoiled gradient echo (spoiled-GE) image acquired with flip angle (α) = 20° and repetition time (TR) = 14 ms, echo time (TE) = 2.4 ms, in-plane resolution = 0.938×0.938 mm, through-plane resolution = 1mm, acquisition matrix = 256×256, and number of slices = 130; for MIG whole-brain images were collected using spoiled gradient echo (SPGR) T1-weighted sagittal anatomical scan (repetition time [TR] = 7.24 ms, echo time [TE] = 2.78 ms, flip angle = 12°, FOV = 232×232 mm, matrix = 256×256, voxel size = 0.90 mm3) | 6.0.1 | Sagittal | Centos6_x86_64 |
| 38 | Sydney | 3T GE MR750 | 3D T1-weighted sequence. TR=7.2 msec; TE=2.78 msec; matrix =256; FOV=240; No. slices=196; thick=0.9mm; inplane resolution=0.9375 | 5.1 | Coronal | Linux_Ubuntu16.04 lts 64bit |
| 39 | TAD | 3T GE MR750 | TR/TE/TI=8.2/3.2/600 ms; flip angle=12°; 156 axial slices; FOV=25.6 cm; matrix=256 mm x 256 mm, isotropic voxel=1 mm, total scan time: 3:40 | 6 | Axial | Linux |
| 40 | TIGER | 3T GE MR750 | TR/TE/TI=8.2/3.2/600 ms; flip angle=12°; 156 axial slices; FOV=25.6 cm; matrix=256 mm x 256 mm, isotropic voxel=1 mm, total scan time: 3:40 | 6 | Axial | Linux |
| 41 | TiPs | 3T Siemens MAGNETOM Prisma_fit | MPRAGE sequence: TR 2300 ms, TE 3.03 ms, α 9°, 192 contiguous sagittal slices, in-plane field of view 256 mm, voxel resolution 1 mm ~1 mm ~1 mm; acquisition time 5:21 min | 5.3 | Sagittal | Linux |
| 3D BRAVO: 3D Brain Volume Imaging; 3D MPRAGE: 3D Magnetization Prepared Rapid Gradient Echo; 3D T1: Three-dimensional T1-weighted Imaging; 3D-TFE: 3D Turbo Field Echo; ACQ: Acquisition; ADNI: Alzheimer’s Disease Neuroimaging Initiative; AP: Anterior-Posterior; CentOS: Community Enterprise Operating System; FFE: Fast Field Echo; FH: Feet-Head; FLAIR: Fluid-Attenuated Inversion Recovery; FOV: Field of View; FSPGR: Fast Spoiled Gradient Recalled Echo; MP-RAGE: Magnetization-Prepared Rapid Gradient Echo; MXP: Matrix Protocol; RL: Right-Left; ROW: Row Encoding Direction; SPGR: Spoiled Gradient Echo; TI: Inversion Time; TR: Repetition Time; TE: Echo Time. | | | | | | |

**Supplementary Table S5.** PERMANOVA and MDMR results for site effects before and after neuroComBat harmonization in SV, CSA, and CT

| Metric | State | Df (site) | Df (residual) | PERMANOVA R² | PERMANOVA F | PERMANOVA p | MDMR R² | MDMR F | MDMR F |
| --- | --- | --- | --- | --- | --- | --- | --- | --- | --- |
| SV | Pre-harmonization | 46 | 7025 | 0.2573 | 52.9039 | <0.001 | 0.2573 | 52.9039 | <0.001 |
| SV | Post-harmonization | 46 | 7025 | 0.0021 | 0.3276 | 1.000 | 0.0021 | 0.3276 | 1.000 |
| CT | Pre-harmonization | 46 | 6683 | 0.3825 | 89.9887 | <0.001 | 0.3825 | 89.9887 | <0.001 |
| CT | Post-harmonization | 46 | 6683 | 0.0020 | 0.2860 | 1.000 | 0.0020 | 0.2860 | 1.000 |
| CSA | Pre-harmonization | 46 | 6034 | 0.1357 | 20.5931 | <0.001 | 0.1357 | 20.5931 | <0.001 |
| CSA | Post-harmonization | 46 | 6034 | 0.0037 | 0.4825 | 1.000 | 0.0037 | 0.4825 | 1.000 |
| PERMANOVA, permutational multivariate analysis of variance; MDMR, multivariate distance matrix regression; CT, cortical thickness; CSA, cortical surface area; SV, subcortical volume; Df, degrees of freedom. P values were based on 999 permutations. | | | | | | | | | |

**Supplementary Table S6.** ENIGMA – Major Depressive Disorder Working Group PBSI score differences between MDDs and HCs in CT, CSA and SV

| Metric | Contrast | Cohen’s d  (95% CI) | Mean (SD)  MDD | Mean (SD)  HC | estimate | SE | df | t.ratio | FDR |
| --- | --- | --- | --- | --- | --- | --- | --- | --- | --- |
| CT | MDD - HC | -0.264  [-0.319, -0.209] | 0.801  (3.20E-02) | 0.809  (2.85E-02) | -8.00E-03 | 5.24E-04 | 1.53E+04 | -1.53E+01 | 3.10E-52 |
| CSA | MDD - HC | -0.079  [-0.134, -0.024] | 0.984  (2.42E-03) | 0.984  (2.29E-03) | -1.88E-04 | 5.24E-04 | 1.53E+04 | -3.59E-01 | 7.20E-01 |
| SV | MDD - HC | 0.029  [-0.026, 0.084] | 0.959  (1.14E-02) | 0.959  (1.13E-02) | 3.31E-04 | 5.24E-04 | 1.53E+04 | 6.31E-01 | 5.28E-01 |
| SD, standard deviation; SE, standard error; df, degrees of freedom; FDR, false discovery rate; CT, cortical thickness; CSA, cortical surface area; SV, subcortical volume; MDD, major depressive disorder; HC, healthy control. | | | | | | | | | |

**Supplementary Table S7.** ENIGMA – Major Depressive Disorder Working Group CVR differences between patients with MDDs and HCs in CT

| Region | CVR  (MDD vs HC) | Higher variation in MDD (%) | P-Value | FDR | N  MDD | N  HC | 95% CI |
| --- | --- | --- | --- | --- | --- | --- | --- |
| Right transverse temporal gyrus | 1.068 | 6.8 | 1.00E-03 | 0.030 | 2566 | 2561 | [1.027; 1.111] |
| Left precentral gyrus | 1.068 | 6.8 | 1.00E-03 | 0.030 | 2560 | 2557 | [1.027; 1.110] |
| Right isthmus cingulate cortex | 1.066 | 6.6 | 1.00E-03 | 0.030 | 2569 | 2570 | [1.025; 1.109] |
| Left isthmus cingulate cortex | 1.061 | 6.1 | 3.00E-03 | 0.041 | 2564 | 2569 | [1.020; 1.103] |
| Left fusiform gyrus | 1.061 | 6.1 | 3.00E-03 | 0.041 | 2567 | 2570 | [1.020; 1.103] |
| Left rostral anterior cingulate cortex | 1.059 | 5.9 | 4.00E-03 | 0.043 | 2562 | 2564 | [1.019; 1.102] |
| Left insula | 1.058 | 5.8 | 5.00E-03 | 0.046 | 2555 | 2553 | [1.017; 1.100] |
| Right precentral gyrus | 1.057 | 5.7 | 6.00E-03 | 0.048 | 2565 | 2564 | [1.016; 1.098] |
| Right fusiform gyrus | 1.053 | 5.3 | 9.00E-03 | 0.062 | 2566 | 2572 | [1.013; 1.095] |
| Left superior temporal gyrus | 1.053 | 5.3 | 1.00E-02 | 0.062 | 2496 | 2505 | [1.012; 1.095] |
| Right superior temporal gyrus | 1.053 | 5.3 | 1.00E-02 | 0.062 | 2543 | 2549 | [1.012; 1.095] |
| Left posterior cingulate cortex | 1.052 | 5.2 | 1.10E-02 | 0.062 | 2569 | 2568 | [1.012; 1.093] |
| Right cuneus | 1.048 | 4.8 | 1.70E-02 | 0.090 | 2555 | 2561 | [1.008; 1.090] |
| Right inferior temporal gyrus | 1.045 | 4.5 | 2.60E-02 | 0.125 | 2553 | 2561 | [1.005; 1.087] |
| Right insula | 1.041 | 4.1 | 4.10E-02 | 0.170 | 2551 | 2542 | [1.002; 1.083] |
| Right rostral anterior cingulate cortex | 1.041 | 4.1 | 4.40E-02 | 0.170 | 2567 | 2568 | [1.001; 1.082] |
| Left precuneus | 1.041 | 4.1 | 4.40E-02 | 0.170 | 2562 | 2569 | [1.001; 1.082] |
| Right paracentral lobule | 1.04 | 4.0 | 4.80E-02 | 0.170 | 2572 | 2568 | [1.000; 1.081] |
| Left caudal anterior cingulate cortex | 1.04 | 4.0 | 5.10E-02 | 0.170 | 2572 | 2559 | [1.000; 1.081] |
| Right precuneus | 1.039 | 3.9 | 5.30E-02 | 0.170 | 2569 | 2570 | [0.999; 1.080] |
| Left supramarginal cortex | 1.039 | 3.9 | 5.70E-02 | 0.170 | 2547 | 2548 | [0.999; 1.080] |
| Left postcentral gyrus | 1.038 | 3.8 | 5.90E-02 | 0.170 | 2561 | 2544 | [0.999; 1.079] |
| Left inferior temporal gyrus | 1.038 | 3.8 | 6.00E-02 | 0.170 | 2535 | 2553 | [0.998; 1.079] |
| Right postcentral gyrus | 1.038 | 3.8 | 6.00E-02 | 0.170 | 2566 | 2556 | [0.998; 1.079] |
| Left medial orbitofrontal cortex | 1.038 | 3.8 | 6.30E-02 | 0.171 | 2559 | 2551 | [0.998; 1.079] |
| Left middle temporal gyrus | 1.036 | 3.6 | 7.40E-02 | 0.186 | 2480 | 2525 | [0.996; 1.078] |
| Right lateral occipital cortex | 1.036 | 3.6 | 7.30E-02 | 0.186 | 2567 | 2566 | [0.997; 1.077] |
| Right posterior cingulate cortex | 1.036 | 3.6 | 7.70E-02 | 0.188 | 2571 | 2571 | [0.996; 1.077] |
| Right caudal anterior cingulate cortex | 1.033 | 3.3 | 1.00E-01 | 0.234 | 2569 | 2572 | [0.994; 1.074] |
| Right superior frontal gyrus | 1.032 | 3.2 | 1.17E-01 | 0.264 | 2568 | 2569 | [0.992; 1.072] |
| Right inferior parietal cortex | 1.031 | 3.1 | 1.22E-01 | 0.268 | 2565 | 2567 | [0.992; 1.072] |
| Right medial orbitofrontal cortex | 1.028 | 2.8 | 1.60E-01 | 0.341 | 2564 | 2563 | [0.989; 1.069] |
| Left temporal pole | 1.027 | 2.7 | 1.78E-01 | 0.366 | 2568 | 2563 | [0.988; 1.068] |
| Left parahippocampal gyrus | 1.026 | 2.6 | 1.97E-01 | 0.383 | 2566 | 2572 | [0.987; 1.067] |
| Left superior frontal gyrus | 1.024 | 2.4 | 2.31E-01 | 0.436 | 2569 | 2570 | [0.985; 1.065] |
| Right rostral middle frontal gyrus | 1.024 | 2.4 | 2.40E-01 | 0.441 | 2565 | 2568 | [0.985; 1.064] |
| Left pars opercularis | 1.022 | 2.2 | 2.70E-01 | 0.483 | 2569 | 2567 | [0.983; 1.063] |
| Right frontal pole | 1.022 | 2.2 | 2.78E-01 | 0.484 | 2569 | 2571 | [0.983; 1.063] |
| Right supramarginal gyrus | 1.02 | 2.0 | 3.13E-01 | 0.518 | 2554 | 2562 | [0.982; 1.061] |
| Left paracentral lobule | 1.02 | 2.0 | 3.26E-01 | 0.527 | 2570 | 2569 | [0.981; 1.060] |
| Left frontal pole | 1.02 | 2.0 | 3.38E-01 | 0.534 | 2573 | 2571 | [0.980; 1.060] |
| Right banks superior temporal sulcus | 1.019 | 1.9 | 3.52E-01 | 0.543 | 2548 | 2549 | [0.980; 1.060] |
| Right parahippocampal gyrus | 1.016 | 1.6 | 4.39E-01 | 0.649 | 2570 | 2571 | [0.977; 1.056] |
| Right superior parietal cortex | 1.015 | 1.5 | 4.65E-01 | 0.672 | 2571 | 2568 | [0.976; 1.055] |
| Right lingual gyrus | 1.014 | 1.4 | 4.88E-01 | 0.689 | 2563 | 2567 | [0.975; 1.054] |
| Left pars triangularis | 1.014 | 1.4 | 4.96E-01 | 0.689 | 2569 | 2565 | [0.975; 1.054] |
| Left transverse temporal gyrus | 1.012 | 1.2 | 5.69E-01 | 0.773 | 2567 | 2566 | [0.973; 1.052] |
| Right middle temporal gyrus | 1.011 | 1.1 | 5.80E-01 | 0.773 | 2559 | 2563 | [0.973; 1.051] |
| Right pericalcarine cortex | 1.011 | 1.1 | 5.96E-01 | 0.780 | 2553 | 2555 | [0.972; 1.051] |
| Left entorhinal cortex | 1.01 | 1.0 | 6.32E-01 | 0.786 | 2502 | 2525 | [0.971; 1.051] |
| Left lateral orbitofrontal cortex | 1.01 | 1.0 | 6.38E-01 | 0.786 | 2568 | 2561 | [0.971; 1.050] |
| Left pars orbitalis | 1.01 | 1.0 | 6.42E-01 | 0.786 | 2568 | 2566 | [0.971; 1.050] |
| Left superior parietal cortex | 1.009 | 0.9 | 6.47E-01 | 0.786 | 2569 | 2562 | [0.971; 1.049] |
| Right entorhinal cortex | 1.009 | 0.9 | 6.65E-01 | 0.794 | 2471 | 2492 | [0.970; 1.050] |
| Left rostral middle frontal gyrus | 1.007 | 0.7 | 7.46E-01 | 0.841 | 2569 | 2567 | [0.968; 1.047] |
| Left banks superior temporal sulcus | 1.007 | 0.7 | 7.55E-01 | 0.841 | 2450 | 2496 | [0.968; 1.047] |
| Right pars opercularis | 1.006 | 0.6 | 7.71E-01 | 0.841 | 2565 | 2568 | [0.968; 1.046] |
| Left caudal middle frontal gyrus | 1.006 | 0.6 | 7.80E-01 | 0.841 | 2566 | 2562 | [0.968; 1.046] |
| Left pericalcarine cortex | 1.006 | 0.6 | 7.91E-01 | 0.841 | 2562 | 2559 | [0.967; 1.046] |
| Left lateral occipital cortex | 1.006 | 0.6 | 7.91E-01 | 0.841 | 2566 | 2567 | [0.967; 1.045] |
| Left cuneus | 1.005 | 0.5 | 8.05E-01 | 0.842 | 2563 | 2560 | [0.967; 1.045] |
| Left lingual gyrus | 1.002 | 0.2 | 9.35E-01 | 0.949 | 2565 | 2560 | [0.964; 1.041] |
| Left inferior parietal cortex | 1 | 0.0 | 9.85E-01 | 0.985 | 2565 | 2564 | [0.962; 1.039] |
| Right pars triangularis | 0.997 | -0.3 | 8.82E-01 | 0.909 | 2569 | 2566 | [0.959; 1.036] |
| Right lateral orbitofrontal cortex | 0.992 | -0.8 | 6.85E-01 | 0.804 | 2568 | 2566 | [0.954; 1.031] |
| Right temporal pole | 0.984 | -1.6 | 4.21E-01 | 0.636 | 2570 | 2562 | [0.946; 1.023] |
| Right pars orbitalis | 0.98 | -2.0 | 3.06E-01 | 0.518 | 2569 | 2573 | [0.942; 1.019] |
| Right caudal middle frontal gyrus | 0.974 | -2.6 | 1.91E-01 | 0.382 | 2568 | 2566 | [0.937; 1.013] |
| CVR, coefficient of variation ratio; MDD, major depressive disorder; HC, healthy control; FDR, false discovery rate; CI, confidence interval. | | | | | | | |

**Supplementary Table S8.** ENIGMA – Major Depressive Disorder Working Group ANOVA of demographic variables on PBSI score differences between MDD patients and HCs in CT.

| Analysis of Variance Table | | | | | | |
| --- | --- | --- | --- | --- | --- | --- |
| **Response: PBSI** | | | | | | |
|  | Df | Sum Sq | Mean Sq | F value | Pr(>F) |  |
| Group | 1.00E+00 | 1.06E-02 | 1.06E-02 | 1.15E+01 | 6.70E-04 | *** |
| Age | 1.00E+00 | 6.42E-03 | 6.42E-03 | 7.01E+00 | 8.13E-03 | ** |
| Sex | 1.00E+00 | 6.68E-03 | 6.68E-03 | 7.29E+00 | 6.95E-03 | ** |
| Age x Group | 1.00E+00 | 2.99E-04 | 2.99E-04 | 3.30E-01 | 5.67E-01 |  |
| Sex x Group | 1.00E+00 | 4.22E-03 | 4.22E-03 | 4.60E+00 | 6.39E-02 | . |
| Residuals | 5.10E+03 | 4.68E+00 | 9.16E-04 |  |  |  |
| Signif. codes: 0.0001 ‘***’ 0.001 ‘**’ 0.01 ‘*’ 0.05 ‘.’ 0.1 ‘ ’ 1 | | | | | | |
| PBSI, person-based similarity index; Df, degrees of freedom; Sq, square. | | | | | | |

**Supplementary Table S9.** ENIGMA – Major Depressive Disorder Working Group PBSI score differences between age-stratified MDD patients and HCs in CT.

| Metric | Contrast | Cohen’s d  (95% CI) | Mean (SD)  MDD Adult | Mean (SD)  MDD Adol. | Mean (SD)  HC Adult | Mean (SD)  HC Adol. | estimate | SE | df | t.ratio | FDR |
| --- | --- | --- | --- | --- | --- | --- | --- | --- | --- | --- | --- |
| CT | HC Adol. -  HC Adult | -0.102  [-0.218, 0.014] |  |  | 0.809  (2.83E-02) | 0.806  (2.97E-02) | -2.80E-03 | 1.70E-03 | 5.11E+03 | -1.65E+00 | 9.93E-02 |
|  | MDD Adol. -  MDD Adult | -0.098  [-0.214, 0.018] | 0.801  (3.15E-02) | 0.798  (3.48E-02) |  |  | -3.15E-03 | 1.70E-03 | 5.11E+03 | -1.85E+00 | 7.77E-02 |
|  | MDD Adult -  HC Adult | -0.266  [-0.325, -0.207] | 0.801  (3.15E-02) |  | 0.809  (2.83E-02) |  | -8.48E-03 | 9.47E-04 | 5.11E+03 | -8.95E+00 | 2.82E-18 |
|  | MDD Adol. -  HC Adol. | -0.254  [-0.408, -0.100] |  | 0.798  (3.48E-02) |  | 0.806  (2.97E-02) | -8.84E-03 | 2.24E-03 | 5.11E+03 | -3.94E+00 | 1.64E-04 |
| Adol., Adolescent; SD, standard deviation; MDD, major depressive disorder; HC, healthy control; SE, standard error; df, degrees of freedom; FDR, false discovery rate; CT, cortical thickness | | | | | | | | | | | |

**Supplementary Table S10.** ENIGMA – Major Depressive Disorder Working Group PBSI score differences between sex-stratified MDD patients and HCs in CT.

| Metric | Contrast | Cohen’s d  95% CI) | Mean (SD)  MDD Male | Mean (SD)  MDD Female | Mean (SD)  HC Male | Mean (SD)  HC Female | estimate | SE | df | t.ratio | FDR |
| --- | --- | --- | --- | --- | --- | --- | --- | --- | --- | --- | --- |
| CT | HC Male -  HC Female | 0.118  [-0.002, 0.238] |  |  | 0.811  (2.73E-02) | 0.807  (2.91E-02) | 3.32E-03 | 1.25E-03 | 5.11E+03 | 2.66E+00 | 5.57E-02 |
|  | MDD Male - MDD Female | -0.013  [-0.133, 0.107] | 0.800  (3.27E-02) | 0.801  (3.16E-02) |  |  | -4.64E-04 | 1.25E-03 | 5.11E+03 | -3.72E-01 | 7.10E-01 |
|  | MDD Male - HC Male | -0.346  [-0.436, -0.256] | 0.800  (3.27E-02) |  | 0.811  (2.73E-02) |  | -1.66E-03 | 5.11E+03 | 6.34E+00 | -7.43E-10 | 1.66E-03 |
|  | MDD Female - HC Female | -0.218  [-0.288, -0.148] |  | 0.801  (3.16E-02) |  | 0.807  (2.91E-02) | -6.77E-03 | 1.35E-03 | 5.11E+03 | -5.01E+00 | 1.11E-06 |
| SD, standard deviation; MDD, major depressive disorder; HC, healthy control; SE, standard error; df, degrees of freedom; FDR, false discovery rate; CT, cortical thickness | | | | | | | | | | | |

**Supplementary Table S11.** ENIGMA – Major Depressive Disorder Working Group CVR differences between adult MDD patients and age-/sex-matched adult HCs in CT.

| Region | CVR  (adult MDD vs adult HC) | Higher variation in adult MDD (%) | P-value | FDR | N  adult MDD | N  adult HC | 95% CI |
| --- | --- | --- | --- | --- | --- | --- | --- |
| Left precentral gyrus | 1.078 | 7.8 | 0.00E+00 | 0.029 | 2234 | 2231 | [1.034; 1.124] |
| Right transverse temporal gyrus | 1.069 | 6.9 | 2.00E-03 | 0.062 | 2239 | 2233 | [1.025; 1.115] |
| Left rostral anterior cingulate cortex | 1.06 | 6 | 7.00E-03 | 0.148 | 2235 | 2237 | [1.016; 1.105] |
| Right precentral gyrus | 1.056 | 5.6 | 1.10E-02 | 0.153 | 2237 | 2237 | [1.013; 1.101] |
| Left superior temporal gyrus | 1.054 | 5.4 | 1.50E-02 | 0.153 | 2173 | 2188 | [1.010; 1.099] |
| Right isthmus cingulate cortex | 1.052 | 5.2 | 1.80E-02 | 0.153 | 2241 | 2242 | [1.009; 1.097] |
| Left posterior cingulate cortex | 1.051 | 5.1 | 1.80E-02 | 0.153 | 2242 | 2242 | [1.009; 1.096] |
| Right superior temporal gyrus | 1.051 | 5.1 | 1.90E-02 | 0.153 | 2219 | 2224 | [1.008; 1.096] |
| Left fusiform gyrus | 1.05 | 5 | 2.00E-02 | 0.153 | 2240 | 2242 | [1.008; 1.095] |
| Left isthmus cingulate cortex | 1.047 | 4.7 | 3.10E-02 | 0.176 | 2238 | 2243 | [1.004; 1.092] |
| Right cuneus | 1.047 | 4.7 | 3.10E-02 | 0.176 | 2232 | 2237 | [1.004; 1.091] |
| Right fusiform gyrus | 1.047 | 4.7 | 3.00E-02 | 0.176 | 2240 | 2244 | [1.004; 1.091] |
| Left insula | 1.04 | 4 | 6.60E-02 | 0.319 | 2228 | 2228 | [0.997; 1.084] |
| Left postcentral gyrus | 1.04 | 4 | 6.20E-02 | 0.319 | 2234 | 2218 | [0.998; 1.085] |
| Right postcentral gyrus | 1.037 | 3.7 | 8.90E-02 | 0.375 | 2239 | 2231 | [0.995; 1.081] |
| Right paracentral lobule | 1.036 | 3.6 | 9.60E-02 | 0.375 | 2244 | 2241 | [0.994; 1.080] |
| Left caudal anterior cingulate cortex | 1.033 | 3.3 | 1.28E-01 | 0.457 | 2244 | 2234 | [0.991; 1.077] |
| Right rostral anterior cingulate cortex | 1.032 | 3.2 | 1.41E-01 | 0.48 | 2240 | 2241 | [0.990; 1.076] |
| Left supramarginal cortex | 1.03 | 3 | 1.72E-01 | 0.558 | 2220 | 2226 | [0.987; 1.073] |
| Right insula | 1.029 | 2.9 | 1.81E-01 | 0.559 | 2223 | 2218 | [0.987; 1.073] |
| Right inferior temporal gyrus | 1.027 | 2.7 | 2.20E-01 | 0.651 | 2225 | 2233 | [0.985; 1.070] |
| Left parahippocampal gyrus | 1.025 | 2.5 | 2.42E-01 | 0.687 | 2240 | 2244 | [0.983; 1.069] |
| Left precuneus | 1.024 | 2.4 | 2.76E-01 | 0.693 | 2235 | 2242 | [0.982; 1.067] |
| Left pars opercularis | 1.023 | 2.3 | 2.77E-01 | 0.693 | 2243 | 2242 | [0.982; 1.067] |
| Right caudal anterior cingulate cortex | 1.022 | 2.2 | 3.03E-01 | 0.693 | 2241 | 2244 | [0.981; 1.066] |
| Right lateral occipital cortex | 1.022 | 2.2 | 3.14E-01 | 0.693 | 2240 | 2239 | [0.980; 1.065] |
| Right precuneus | 1.022 | 2.2 | 3.16E-01 | 0.693 | 2241 | 2243 | [0.980; 1.065] |
| Left medial orbitofrontal cortex | 1.021 | 2.1 | 3.31E-01 | 0.693 | 2232 | 2224 | [0.979; 1.065] |
| Left inferior temporal gyrus | 1.02 | 2 | 3.52E-01 | 0.693 | 2209 | 2228 | [0.979; 1.064] |
| Right posterior cingulate cortex | 1.02 | 2 | 3.46E-01 | 0.693 | 2243 | 2244 | [0.979; 1.064] |
| Right medial orbitofrontal cortex | 1.019 | 1.9 | 3.95E-01 | 0.726 | 2236 | 2236 | [0.977; 1.062] |
| Left transverse temporal gyrus | 1.018 | 1.8 | 4.21E-01 | 0.753 | 2239 | 2239 | [0.976; 1.061] |
| Right parahippocampal gyrus | 1.017 | 1.7 | 4.51E-01 | 0.753 | 2243 | 2243 | [0.975; 1.060] |
| Left middle temporal gyrus | 1.016 | 1.6 | 4.81E-01 | 0.779 | 2166 | 2201 | [0.974; 1.059] |
| Left frontal pole | 1.014 | 1.4 | 5.23E-01 | 0.803 | 2245 | 2243 | [0.973; 1.057] |
| Left temporal pole | 1.013 | 1.3 | 5.64E-01 | 0.803 | 2240 | 2236 | [0.971; 1.056] |
| Right banks superior temporal sulcus | 1.013 | 1.3 | 5.58E-01 | 0.803 | 2228 | 2226 | [0.972; 1.056] |
| Right superior frontal gyrus | 1.013 | 1.3 | 5.63E-01 | 0.803 | 2241 | 2241 | [0.971; 1.056] |
| Left lateral orbitofrontal cortex | 1.012 | 1.2 | 5.79E-01 | 0.803 | 2240 | 2233 | [0.971; 1.055] |
| Left paracentral lobule | 1.012 | 1.2 | 5.71E-01 | 0.803 | 2243 | 2241 | [0.971; 1.055] |
| Right pericalcarine cortex | 1.01 | 1 | 6.66E-01 | 0.906 | 2228 | 2230 | [0.968; 1.053] |
| Left pericalcarine cortex | 1.008 | 0.8 | 7.37E-01 | 0.922 | 2235 | 2234 | [0.966; 1.051] |
| Right frontal pole | 1.008 | 0.8 | 7.29E-01 | 0.922 | 2241 | 2245 | [0.966; 1.051] |
| Right supramarginal gyrus | 1.008 | 0.8 | 7.17E-01 | 0.922 | 2227 | 2235 | [0.967; 1.051] |
| Right inferior parietal cortex | 1.007 | 0.7 | 7.70E-01 | 0.935 | 2238 | 2239 | [0.966; 1.049] |
| Left superior frontal gyrus | 1.006 | 0.6 | 8.00E-01 | 0.95 | 2242 | 2242 | [0.965; 1.048] |
| Right rostral middle frontal gyrus | 1.004 | 0.4 | 8.70E-01 | 0.97 | 2238 | 2240 | [0.963; 1.046] |
| Left caudal middle frontal gyrus | 1.002 | 0.2 | 9.33E-01 | 0.988 | 2238 | 2235 | [0.961; 1.045] |
| Left entorhinal cortex | 1.002 | 0.2 | 9.44E-01 | 0.988 | 2180 | 2200 | [0.960; 1.045] |
| Left lateral occipital cortex | 1.002 | 0.2 | 9.29E-01 | 0.988 | 2239 | 2239 | [0.961; 1.045] |
| Left pars triangularis | 1 | 0 | 9.94E-01 | 0.994 | 2242 | 2240 | [0.959; 1.043] |
| Left rostral middle frontal gyrus | 0.999 | -0.1 | 9.74E-01 | 0.988 | 2242 | 2240 | [0.959; 1.042] |
| Right superior parietal cortex | 0.999 | -0.1 | 9.74E-01 | 0.988 | 2244 | 2242 | [0.959; 1.042] |
| Left lingual gyrus | 0.998 | -0.2 | 9.37E-01 | 0.988 | 2237 | 2233 | [0.957; 1.041] |
| Left banks superior temporal sulcus | 0.996 | -0.4 | 8.60E-01 | 0.97 | 2139 | 2180 | [0.955; 1.039] |
| Right lingual gyrus | 0.996 | -0.4 | 8.67E-01 | 0.97 | 2236 | 2240 | [0.956; 1.038] |
| Right entorhinal cortex | 0.994 | -0.6 | 8.11E-01 | 0.95 | 2149 | 2175 | [0.953; 1.038] |
| Left superior parietal cortex | 0.993 | -0.7 | 7.46E-01 | 0.922 | 2242 | 2234 | [0.952; 1.035] |
| Left pars orbitalis | 0.992 | -0.8 | 7.21E-01 | 0.922 | 2241 | 2239 | [0.952; 1.034] |
| Right pars opercularis | 0.986 | -1.4 | 5.32E-01 | 0.803 | 2238 | 2242 | [0.946; 1.028] |
| Left cuneus | 0.984 | -1.6 | 4.54E-01 | 0.753 | 2237 | 2236 | [0.944; 1.026] |
| Right temporal pole | 0.983 | -1.7 | 4.40E-01 | 0.753 | 2242 | 2236 | [0.943; 1.025] |
| Left inferior parietal cortex | 0.982 | -1.8 | 3.93E-01 | 0.726 | 2237 | 2236 | [0.942; 1.023] |
| Right middle temporal gyrus | 0.98 | -2 | 3.57E-01 | 0.693 | 2236 | 2235 | [0.940; 1.022] |
| Right lateral orbitofrontal cortex | 0.978 | -2.2 | 2.89E-01 | 0.693 | 2242 | 2238 | [0.938; 1.019] |
| Right pars triangularis | 0.977 | -2.3 | 2.75E-01 | 0.693 | 2241 | 2238 | [0.937; 1.018] |
| Right pars orbitalis | 0.966 | -3.4 | 9.90E-02 | 0.375 | 2244 | 2245 | [0.926; 1.007] |
| Right caudal middle frontal gyrus | 0.965 | -3.5 | 9.00E-02 | 0.375 | 2241 | 2238 | [0.925; 1.006] |
| CVR, coefficient of variation ratio; MDD, major depressive disorder; HC, healthy control; FDR, false discovery rate; CI, confidence interval. | | | | | | | |

**Supplementary Table S12.** ENIGMA – Major Depressive Disorder Working Group CVR differences between adolescent MDD patients and age-/sex-matched adolescent HCs in CT.

| Region | CVR  (adolescent MDD vs adolescent HC) | Higher variation in adolescent MDD (%) | P-value | FDR | N  adolescent MDD | N  adolescent HC | 95% CI |
| --- | --- | --- | --- | --- | --- | --- | --- |
| Right inferior parietal cortex | 1.233 | 23.3 | 0.00E+00 | 0.012 | 327 | 328 | [1.106; 1.375] |
| Right inferior temporal gyrus | 1.18 | 18 | 3.00E-03 | 0.073 | 328 | 328 | [1.058; 1.315] |
| Right middle temporal gyrus | 1.167 | 16.7 | 5.00E-03 | 0.073 | 323 | 328 | [1.047; 1.302] |
| Right superior parietal cortex | 1.167 | 16.7 | 5.00E-03 | 0.073 | 327 | 326 | [1.047; 1.301] |
| Right pars opercularis | 1.165 | 16.5 | 6.00E-03 | 0.073 | 327 | 326 | [1.045; 1.299] |
| Right lateral occipital cortex | 1.163 | 16.3 | 6.00E-03 | 0.073 | 327 | 327 | [1.043; 1.297] |
| Left superior parietal cortex | 1.156 | 15.6 | 9.00E-03 | 0.089 | 327 | 328 | [1.036; 1.288] |
| Left inferior parietal cortex | 1.134 | 13.4 | 2.30E-02 | 0.184 | 328 | 328 | [1.017; 1.264] |
| Left middle temporal gyrus | 1.134 | 13.4 | 2.50E-02 | 0.184 | 314 | 324 | [1.016; 1.266] |
| Right precentral gyrus | 1.13 | 13 | 2.70E-02 | 0.184 | 328 | 327 | [1.014; 1.260] |
| Left precentral gyrus | 1.124 | 12.4 | 3.50E-02 | 0.209 | 326 | 326 | [1.008; 1.253] |
| Left inferior temporal gyrus | 1.122 | 12.2 | 3.90E-02 | 0.209 | 326 | 325 | [1.006; 1.251] |
| Left temporal pole | 1.122 | 12.2 | 4.00E-02 | 0.209 | 328 | 327 | [1.005; 1.251] |
| Right supramarginal gyrus | 1.118 | 11.8 | 4.40E-02 | 0.211 | 327 | 327 | [1.003; 1.246] |
| Left cuneus | 1.117 | 11.7 | 4.70E-02 | 0.211 | 326 | 324 | [1.002; 1.246] |
| Right entorhinal cortex | 1.114 | 11.4 | 5.70E-02 | 0.243 | 322 | 317 | [0.997; 1.245] |
| Left banks superior temporal sulcus | 1.109 | 10.9 | 6.90E-02 | 0.268 | 311 | 316 | [0.992; 1.239] |
| Left insula | 1.105 | 10.5 | 7.10E-02 | 0.268 | 327 | 325 | [0.991; 1.233] |
| Right lingual gyrus | 1.103 | 10.3 | 7.70E-02 | 0.277 | 327 | 327 | [0.989; 1.230] |
| Left superior frontal gyrus | 1.099 | 9.9 | 8.80E-02 | 0.299 | 327 | 328 | [0.986; 1.225] |
| Right superior frontal gyrus | 1.09 | 9 | 1.20E-01 | 0.37 | 327 | 328 | [0.978; 1.215] |
| Left medial orbitofrontal cortex | 1.087 | 8.7 | 1.32E-01 | 0.38 | 327 | 327 | [0.975; 1.212] |
| Right rostral anterior cingulate cortex | 1.087 | 8.7 | 1.34E-01 | 0.38 | 327 | 327 | [0.975; 1.213] |
| Right postcentral gyrus | 1.076 | 7.6 | 1.87E-01 | 0.508 | 327 | 325 | [0.965; 1.200] |
| Right isthmus cingulate cortex | 1.074 | 7.4 | 2.03E-01 | 0.532 | 328 | 328 | [0.963; 1.197] |
| Right banks superior temporal sulcus | 1.07 | 7 | 2.28E-01 | 0.56 | 320 | 323 | [0.959; 1.195] |
| Right superior temporal gyrus | 1.069 | 6.9 | 2.31E-01 | 0.56 | 324 | 325 | [0.959; 1.193] |
| Right frontal pole | 1.068 | 6.8 | 2.41E-01 | 0.56 | 328 | 326 | [0.957; 1.192] |
| Left superior temporal gyrus | 1.067 | 6.7 | 2.53E-01 | 0.56 | 323 | 317 | [0.956; 1.191] |
| Left pars triangularis | 1.065 | 6.5 | 2.62E-01 | 0.56 | 327 | 325 | [0.955; 1.188] |
| Left entorhinal cortex | 1.063 | 6.3 | 2.82E-01 | 0.56 | 322 | 325 | [0.952; 1.187] |
| Left caudal anterior cingulate cortex | 1.061 | 6.1 | 2.89E-01 | 0.56 | 328 | 325 | [0.951; 1.184] |
| Right pars orbitalis | 1.06 | 6 | 3.01E-01 | 0.56 | 325 | 328 | [0.950; 1.182] |
| Right rostral middle frontal gyrus | 1.06 | 6 | 2.95E-01 | 0.56 | 327 | 328 | [0.951; 1.182] |
| Right fusiform gyrus | 1.059 | 5.9 | 3.06E-01 | 0.56 | 326 | 328 | [0.950; 1.181] |
| Left paracentral lobule | 1.058 | 5.8 | 3.16E-01 | 0.56 | 327 | 328 | [0.949; 1.180] |
| Right transverse temporal gyrus | 1.058 | 5.8 | 3.16E-01 | 0.56 | 327 | 328 | [0.949; 1.180] |
| Left fusiform gyrus | 1.057 | 5.7 | 3.21E-01 | 0.56 | 327 | 328 | [0.948; 1.179] |
| Left caudal middle frontal gyrus | 1.055 | 5.5 | 3.36E-01 | 0.567 | 328 | 327 | [0.947; 1.177] |
| Left isthmus cingulate cortex | 1.055 | 5.5 | 3.44E-01 | 0.567 | 326 | 326 | [0.946; 1.177] |
| Left precuneus | 1.054 | 5.4 | 3.50E-01 | 0.567 | 327 | 327 | [0.945; 1.175] |
| Left pars opercularis | 1.053 | 5.3 | 3.62E-01 | 0.572 | 326 | 325 | [0.944; 1.174] |
| Right caudal anterior cingulate cortex | 1.05 | 5 | 3.84E-01 | 0.593 | 328 | 328 | [0.942; 1.172] |
| Right medial orbitofrontal cortex | 1.048 | 4.8 | 4.04E-01 | 0.611 | 328 | 327 | [0.940; 1.169] |
| Right pars triangularis | 1.042 | 4.2 | 4.66E-01 | 0.689 | 328 | 328 | [0.935; 1.162] |
| Left supramarginal cortex | 1.04 | 4 | 4.86E-01 | 0.703 | 327 | 322 | [0.933; 1.161] |
| Left postcentral gyrus | 1.039 | 3.9 | 4.96E-01 | 0.703 | 327 | 326 | [0.932; 1.159] |
| Right lateral orbitofrontal cortex | 1.036 | 3.6 | 5.32E-01 | 0.738 | 326 | 328 | [0.929; 1.156] |
| Left pericalcarine cortex | 1.034 | 3.4 | 5.57E-01 | 0.757 | 327 | 325 | [0.927; 1.154] |
| Right precuneus | 1.03 | 3 | 6.08E-01 | 0.796 | 328 | 327 | [0.924; 1.148] |
| Left pars orbitalis | 1.024 | 2.4 | 6.88E-01 | 0.866 | 327 | 327 | [0.918; 1.142] |
| Right paracentral lobule | 1.018 | 1.8 | 7.61E-01 | 0.908 | 328 | 327 | [0.913; 1.135] |
| Left parahippocampal gyrus | 1.013 | 1.3 | 8.32E-01 | 0.954 | 326 | 328 | [0.908; 1.130] |
| Right pericalcarine cortex | 1.012 | 1.2 | 8.46E-01 | 0.954 | 325 | 325 | [0.907; 1.129] |
| Right cuneus | 1.011 | 1.1 | 8.55E-01 | 0.954 | 323 | 324 | [0.906; 1.128] |
| Right insula | 1.008 | 0.8 | 9.01E-01 | 0.954 | 328 | 324 | [0.903; 1.124] |
| Right caudal middle frontal gyrus | 1.007 | 0.7 | 9.05E-01 | 0.954 | 327 | 328 | [0.903; 1.123] |
| Left lateral occipital cortex | 1.006 | 0.6 | 9.26E-01 | 0.954 | 327 | 328 | [0.902; 1.121] |
| Left rostral middle frontal gyrus | 1.006 | 0.6 | 9.15E-01 | 0.954 | 327 | 327 | [0.903; 1.122] |
| Right parahippocampal gyrus | 0.999 | -0.1 | 9.90E-01 | 0.99 | 327 | 328 | [0.896; 1.115] |
| Left frontal pole | 0.997 | -0.3 | 9.67E-01 | 0.982 | 328 | 328 | [0.894; 1.113] |
| Left lingual gyrus | 0.992 | -0.8 | 8.94E-01 | 0.954 | 328 | 327 | [0.890; 1.106] |
| Right temporal pole | 0.986 | -1.4 | 8.10E-01 | 0.95 | 328 | 326 | [0.883; 1.100] |
| Right posterior cingulate cortex | 0.98 | -2 | 7.29E-01 | 0.885 | 328 | 327 | [0.879; 1.093] |
| Left rostral anterior cingulate cortex | 0.979 | -2.1 | 7.15E-01 | 0.884 | 327 | 327 | [0.878; 1.092] |
| Left posterior cingulate cortex | 0.972 | -2.8 | 6.22E-01 | 0.797 | 327 | 326 | [0.872; 1.084] |
| Left lateral orbitofrontal cortex | 0.968 | -3.2 | 5.76E-01 | 0.767 | 328 | 328 | [0.869; 1.080] |
| Left transverse temporal gyrus | 0.915 | -8.5 | 1.13E-01 | 0.365 | 328 | 327 | [0.821; 1.021] |
| CVR, coefficient of variation ratio; MDD, major depressive disorder; HC, healthy control; FDR, false discovery rate; CI, confidence interval. | | | | | | | |

**Supplementary Table S13.** ENIGMA – Major Depressive Disorder Working Group CVR differences between male MDD patients and age-matched male HCs in CT.

| Region | CVR  (male MDD vs male HC) | Higher variation in male MDD (%) | P-value | FDR | N  male MDD | N  male HC | 95% CI |
| --- | --- | --- | --- | --- | --- | --- | --- |
| Right precentral gyrus | 1.136 | 13.6 | 0.00E+00 | 0.009 | 921 | 918 | [1.064; 1.212] |
| Left rostral anterior cingulate cortex | 1.115 | 11.5 | 1.00E-03 | 0.036 | 920 | 921 | [1.045; 1.190] |
| Right temporal pole | 1.1 | 10 | 4.00E-03 | 0.098 | 921 | 918 | [1.030; 1.174] |
| Right transverse temporal gyrus | 1.093 | 9.3 | 7.00E-03 | 0.127 | 921 | 915 | [1.024; 1.167] |
| Left precentral gyrus | 1.075 | 7.5 | 3.00E-02 | 0.382 | 918 | 914 | [1.007; 1.147] |
| Left isthmus cingulate cortex | 1.072 | 7.2 | 3.70E-02 | 0.382 | 919 | 919 | [1.004; 1.144] |
| Right rostral anterior cingulate cortex | 1.069 | 6.9 | 4.50E-02 | 0.382 | 919 | 920 | [1.001; 1.141] |
| Right fusiform gyrus | 1.068 | 6.8 | 4.60E-02 | 0.382 | 917 | 922 | [1.001; 1.140] |
| Right paracentral lobule | 1.066 | 6.6 | 5.30E-02 | 0.382 | 922 | 919 | [0.999; 1.138] |
| Left fusiform gyrus | 1.064 | 6.4 | 6.00E-02 | 0.382 | 918 | 921 | [0.997; 1.135] |
| Right cuneus | 1.064 | 6.4 | 6.20E-02 | 0.382 | 916 | 916 | [0.997; 1.136] |
| Left insula | 1.06 | 6 | 7.80E-02 | 0.411 | 914 | 919 | [0.993; 1.131] |
| Right isthmus cingulate cortex | 1.059 | 5.9 | 8.50E-02 | 0.411 | 919 | 920 | [0.992; 1.130] |
| Right parahippocampal gyrus | 1.059 | 5.9 | 8.20E-02 | 0.411 | 921 | 921 | [0.993; 1.131] |
| Left parahippocampal gyrus | 1.057 | 5.7 | 9.70E-02 | 0.438 | 920 | 921 | [0.990; 1.128] |
| Right entorhinal cortex | 1.057 | 5.7 | 1.03E-01 | 0.438 | 896 | 903 | [0.989; 1.129] |
| Left temporal pole | 1.055 | 5.5 | 1.10E-01 | 0.44 | 921 | 920 | [0.988; 1.126] |
| Right medial orbitofrontal cortex | 1.053 | 5.3 | 1.17E-01 | 0.44 | 920 | 918 | [0.987; 1.124] |
| Right rostral middle frontal gyrus | 1.052 | 5.2 | 1.26E-01 | 0.44 | 918 | 921 | [0.986; 1.123] |
| Left medial orbitofrontal cortex | 1.051 | 5.1 | 1.33E-01 | 0.44 | 917 | 916 | [0.985; 1.122] |
| Right superior frontal gyrus | 1.05 | 5 | 1.44E-01 | 0.44 | 922 | 918 | [0.984; 1.120] |
| Right lateral occipital cortex | 1.049 | 4.9 | 1.45E-01 | 0.44 | 919 | 920 | [0.984; 1.120] |
| Right pericalcarine cortex | 1.049 | 4.9 | 1.49E-01 | 0.44 | 914 | 914 | [0.983; 1.120] |
| Right caudal anterior cingulate cortex | 1.047 | 4.7 | 1.71E-01 | 0.484 | 920 | 922 | [0.981; 1.117] |
| Right inferior temporal gyrus | 1.045 | 4.5 | 1.86E-01 | 0.494 | 912 | 918 | [0.979; 1.115] |
| Right supramarginal gyrus | 1.045 | 4.5 | 1.89E-01 | 0.494 | 910 | 917 | [0.979; 1.115] |
| Left supramarginal cortex | 1.042 | 4.2 | 2.18E-01 | 0.514 | 909 | 912 | [0.976; 1.112] |
| Left transverse temporal gyrus | 1.042 | 4.2 | 2.19E-01 | 0.514 | 920 | 921 | [0.976; 1.112] |
| Left entorhinal cortex | 1.041 | 4.1 | 2.34E-01 | 0.514 | 897 | 912 | [0.975; 1.112] |
| Right inferior parietal cortex | 1.041 | 4.1 | 2.22E-01 | 0.514 | 916 | 918 | [0.976; 1.111] |
| Right precuneus | 1.04 | 4 | 2.34E-01 | 0.514 | 920 | 921 | [0.975; 1.110] |
| Left precuneus | 1.039 | 3.9 | 2.48E-01 | 0.523 | 917 | 919 | [0.974; 1.109] |
| Left caudal anterior cingulate cortex | 1.037 | 3.7 | 2.79E-01 | 0.542 | 921 | 919 | [0.972; 1.107] |
| Right superior parietal cortex | 1.037 | 3.7 | 2.75E-01 | 0.542 | 922 | 920 | [0.972; 1.107] |
| Left pericalcarine cortex | 1.034 | 3.4 | 3.20E-01 | 0.598 | 920 | 915 | [0.969; 1.104] |
| Left posterior cingulate cortex | 1.033 | 3.3 | 3.25E-01 | 0.598 | 921 | 921 | [0.968; 1.103] |
| Right superior temporal gyrus | 1.032 | 3.2 | 3.50E-01 | 0.626 | 910 | 915 | [0.967; 1.101] |
| Right insula | 1.029 | 2.9 | 3.93E-01 | 0.685 | 914 | 914 | [0.964; 1.098] |
| Left inferior temporal gyrus | 1.028 | 2.8 | 4.19E-01 | 0.701 | 905 | 916 | [0.963; 1.097] |
| Left superior parietal cortex | 1.027 | 2.7 | 4.33E-01 | 0.701 | 921 | 915 | [0.962; 1.096] |
| Right lingual gyrus | 1.027 | 2.7 | 4.32E-01 | 0.701 | 917 | 919 | [0.962; 1.096] |
| Left banks superior temporal sulcus | 1.026 | 2.6 | 4.64E-01 | 0.702 | 863 | 895 | [0.960; 1.096] |
| Right posterior cingulate cortex | 1.026 | 2.6 | 4.44E-01 | 0.702 | 922 | 920 | [0.962; 1.095] |
| Right pars opercularis | 1.025 | 2.5 | 4.58E-01 | 0.702 | 919 | 920 | [0.961; 1.094] |
| Left superior frontal gyrus | 1.023 | 2.3 | 5.12E-01 | 0.756 | 919 | 920 | [0.958; 1.091] |
| Left lateral occipital cortex | 1.02 | 2 | 5.68E-01 | 0.822 | 919 | 920 | [0.956; 1.088] |
| Right banks superior temporal sulcus | 1.018 | 1.8 | 6.12E-01 | 0.854 | 912 | 912 | [0.953; 1.086] |
| Left superior temporal gyrus | 1.017 | 1.7 | 6.31E-01 | 0.854 | 883 | 904 | [0.952; 1.086] |
| Left postcentral gyrus | 1.015 | 1.5 | 6.65E-01 | 0.864 | 917 | 909 | [0.951; 1.083] |
| Left middle temporal gyrus | 1.014 | 1.4 | 6.96E-01 | 0.864 | 876 | 911 | [0.949; 1.083] |
| Right lateral orbitofrontal cortex | 1.014 | 1.4 | 6.99E-01 | 0.864 | 920 | 919 | [0.950; 1.082] |
| Left rostral middle frontal gyrus | 1.012 | 1.2 | 7.24E-01 | 0.88 | 921 | 919 | [0.949; 1.080] |
| Right frontal pole | 1.008 | 0.8 | 8.16E-01 | 0.937 | 920 | 920 | [0.944; 1.076] |
| Right middle temporal gyrus | 1.008 | 0.8 | 8.30E-01 | 0.937 | 914 | 918 | [0.944; 1.075] |
| Right postcentral gyrus | 1.007 | 0.7 | 8.55E-01 | 0.937 | 918 | 916 | [0.943; 1.074] |
| Left caudal middle frontal gyrus | 1.006 | 0.6 | 8.56E-01 | 0.937 | 921 | 916 | [0.943; 1.074] |
| Left frontal pole | 1.006 | 0.6 | 8.57E-01 | 0.937 | 922 | 922 | [0.943; 1.074] |
| Left lateral orbitofrontal cortex | 1.004 | 0.4 | 9.17E-01 | 0.959 | 920 | 919 | [0.941; 1.071] |
| Left pars triangularis | 1.003 | 0.3 | 9.32E-01 | 0.96 | 922 | 920 | [0.940; 1.070] |
| Left pars opercularis | 1.001 | 0.1 | 9.88E-01 | 0.988 | 921 | 919 | [0.938; 1.068] |
| Left cuneus | 0.999 | -0.1 | 9.76E-01 | 0.988 | 917 | 918 | [0.936; 1.066] |
| Left paracentral lobule | 0.996 | -0.4 | 9.09E-01 | 0.959 | 921 | 920 | [0.933; 1.063] |
| Left inferior parietal cortex | 0.994 | -0.6 | 8.68E-01 | 0.937 | 917 | 916 | [0.931; 1.061] |
| Right pars orbitalis | 0.991 | -0.9 | 8.03E-01 | 0.937 | 919 | 922 | [0.929; 1.058] |
| Right pars triangularis | 0.986 | -1.4 | 6.84E-01 | 0.864 | 919 | 920 | [0.924; 1.052] |
| Left lingual gyrus | 0.984 | -1.6 | 6.40E-01 | 0.854 | 920 | 915 | [0.922; 1.050] |
| Left pars orbitalis | 0.983 | -1.7 | 6.23E-01 | 0.854 | 919 | 921 | [0.921; 1.049] |
| Right caudal middle frontal gyrus | 0.963 | -3.7 | 2.54E-01 | 0.523 | 919 | 919 | [0.902; 1.027] |
| CVR, coefficient of variation ratio; MDD, major depressive disorder; HC, healthy control; FDR, false discovery rate; CI, confidence interval. | | | | | | | |

**Supplementary Table S14.** ENIGMA – Major Depressive Disorder Working Group CVR differences between female MDD patients and age-matched female HCs in CT.

| Region | CVR  (female MDD vs female HC) | Higher variation in female MDD (%) | P-value | FDR | N  female MDD | N  female HC | 95% CI |
| --- | --- | --- | --- | --- | --- | --- | --- |
| Left superior temporal gyrus | 1.08 | 8 | 2.00E-03 | 0.088 | 1613 | 1601 | [1.029; 1.135] |
| Right isthmus cingulate cortex | 1.07 | 7 | 6.00E-03 | 0.11 | 1650 | 1650 | [1.019; 1.123] |
| Right superior temporal gyrus | 1.07 | 7 | 6.00E-03 | 0.11 | 1633 | 1634 | [1.019; 1.124] |
| Left posterior cingulate cortex | 1.063 | 6.3 | 1.30E-02 | 0.178 | 1648 | 1647 | [1.013; 1.116] |
| Left precentral gyrus | 1.059 | 5.9 | 2.00E-02 | 0.228 | 1642 | 1643 | [1.009; 1.112] |
| Left fusiform gyrus | 1.057 | 5.7 | 2.40E-02 | 0.234 | 1649 | 1649 | [1.007; 1.110] |
| Left insula | 1.054 | 5.4 | 3.30E-02 | 0.254 | 1641 | 1634 | [1.004; 1.107] |
| Left middle temporal gyrus | 1.053 | 5.3 | 4.00E-02 | 0.254 | 1604 | 1614 | [1.002; 1.106] |
| Left isthmus cingulate cortex | 1.052 | 5.2 | 3.90E-02 | 0.254 | 1645 | 1650 | [1.002; 1.105] |
| Right transverse temporal gyrus | 1.051 | 5.1 | 4.40E-02 | 0.254 | 1645 | 1646 | [1.001; 1.104] |
| Left postcentral gyrus | 1.05 | 5 | 4.80E-02 | 0.254 | 1644 | 1635 | [1.000; 1.102] |
| Right insula | 1.05 | 5 | 4.90E-02 | 0.254 | 1637 | 1628 | [1.000; 1.103] |
| Left inferior temporal gyrus | 1.047 | 4.7 | 6.60E-02 | 0.301 | 1630 | 1637 | [0.997; 1.099] |
| Right inferior temporal gyrus | 1.047 | 4.7 | 6.50E-02 | 0.301 | 1641 | 1643 | [0.997; 1.099] |
| Right postcentral gyrus | 1.045 | 4.5 | 7.50E-02 | 0.32 | 1648 | 1640 | [0.995; 1.097] |
| Right fusiform gyrus | 1.043 | 4.3 | 8.60E-02 | 0.342 | 1649 | 1650 | [0.994; 1.095] |
| Left precuneus | 1.041 | 4.1 | 1.08E-01 | 0.373 | 1645 | 1650 | [0.991; 1.092] |
| Right posterior cingulate cortex | 1.041 | 4.1 | 1.05E-01 | 0.373 | 1649 | 1651 | [0.992; 1.093] |
| Left supramarginal cortex | 1.04 | 4 | 1.15E-01 | 0.373 | 1638 | 1636 | [0.990; 1.092] |
| Right cuneus | 1.04 | 4 | 1.14E-01 | 0.373 | 1639 | 1645 | [0.991; 1.092] |
| Left caudal anterior cingulate cortex | 1.039 | 3.9 | 1.22E-01 | 0.377 | 1651 | 1640 | [0.990; 1.091] |
| Left pars opercularis | 1.037 | 3.7 | 1.47E-01 | 0.4 | 1648 | 1648 | [0.988; 1.088] |
| Right precuneus | 1.037 | 3.7 | 1.43E-01 | 0.4 | 1649 | 1649 | [0.988; 1.088] |
| Left medial orbitofrontal cortex | 1.035 | 3.5 | 1.71E-01 | 0.446 | 1642 | 1635 | [0.986; 1.086] |
| Left rostral anterior cingulate cortex | 1.031 | 3.1 | 2.27E-01 | 0.564 | 1642 | 1643 | [0.982; 1.082] |
| Right lateral occipital cortex | 1.03 | 3 | 2.32E-01 | 0.564 | 1648 | 1646 | [0.981; 1.081] |
| Left paracentral lobule | 1.029 | 2.9 | 2.48E-01 | 0.582 | 1649 | 1649 | [0.980; 1.080] |
| Left frontal pole | 1.027 | 2.7 | 2.96E-01 | 0.671 | 1651 | 1649 | [0.978; 1.078] |
| Right frontal pole | 1.024 | 2.4 | 3.57E-01 | 0.729 | 1649 | 1651 | [0.975; 1.075] |
| Right rostral anterior cingulate cortex | 1.024 | 2.4 | 3.54E-01 | 0.729 | 1648 | 1648 | [0.975; 1.075] |
| Left pars orbitalis | 1.023 | 2.3 | 3.64E-01 | 0.729 | 1649 | 1645 | [0.974; 1.074] |
| Right caudal anterior cingulate cortex | 1.022 | 2.2 | 3.82E-01 | 0.731 | 1649 | 1650 | [0.974; 1.073] |
| Right banks superior temporal sulcus | 1.021 | 2.1 | 4.11E-01 | 0.731 | 1636 | 1637 | [0.972; 1.072] |
| Left pars triangularis | 1.02 | 2 | 4.38E-01 | 0.731 | 1647 | 1645 | [0.971; 1.070] |
| Right inferior parietal cortex | 1.02 | 2 | 4.41E-01 | 0.731 | 1649 | 1649 | [0.971; 1.070] |
| Right paracentral lobule | 1.02 | 2 | 4.39E-01 | 0.731 | 1650 | 1649 | [0.971; 1.070] |
| Left superior frontal gyrus | 1.018 | 1.8 | 4.69E-01 | 0.759 | 1650 | 1650 | [0.970; 1.069] |
| Left temporal pole | 1.016 | 1.6 | 5.40E-01 | 0.834 | 1647 | 1643 | [0.967; 1.067] |
| Right middle temporal gyrus | 1.015 | 1.5 | 5.67E-01 | 0.854 | 1645 | 1645 | [0.967; 1.065] |
| Right medial orbitofrontal cortex | 1.014 | 1.4 | 5.90E-01 | 0.854 | 1644 | 1645 | [0.966; 1.064] |
| Right superior frontal gyrus | 1.014 | 1.4 | 5.97E-01 | 0.854 | 1646 | 1651 | [0.966; 1.064] |
| Left lateral orbitofrontal cortex | 1.013 | 1.3 | 6.03E-01 | 0.854 | 1648 | 1642 | [0.965; 1.064] |
| Left lingual gyrus | 1.013 | 1.3 | 6.26E-01 | 0.869 | 1645 | 1645 | [0.965; 1.063] |
| Left cuneus | 1.009 | 0.9 | 7.29E-01 | 0.936 | 1646 | 1642 | [0.961; 1.059] |
| Right lingual gyrus | 1.007 | 0.7 | 7.90E-01 | 0.969 | 1646 | 1648 | [0.959; 1.057] |
| Left parahippocampal gyrus | 1.004 | 0.4 | 8.76E-01 | 0.979 | 1646 | 1651 | [0.956; 1.054] |
| Right precentral gyrus | 1.003 | 0.3 | 8.97E-01 | 0.979 | 1644 | 1646 | [0.956; 1.053] |
| Right supramarginal gyrus | 1.002 | 0.2 | 9.35E-01 | 0.979 | 1644 | 1645 | [0.955; 1.052] |
| Left caudal middle frontal gyrus | 1.001 | 0.1 | 9.68E-01 | 0.979 | 1645 | 1646 | [0.954; 1.051] |
| Left rostral middle frontal gyrus | 0.999 | -0.1 | 9.79E-01 | 0.979 | 1648 | 1648 | [0.952; 1.049] |
| Left superior parietal cortex | 0.999 | -0.1 | 9.66E-01 | 0.979 | 1648 | 1647 | [0.952; 1.048] |
| Right pars triangularis | 0.998 | -0.2 | 9.40E-01 | 0.979 | 1650 | 1646 | [0.951; 1.048] |
| Right rostral middle frontal gyrus | 0.998 | -0.2 | 9.33E-01 | 0.979 | 1647 | 1647 | [0.951; 1.047] |
| Left entorhinal cortex | 0.996 | -0.4 | 8.90E-01 | 0.979 | 1605 | 1613 | [0.948; 1.047] |
| Left banks superior temporal sulcus | 0.995 | -0.5 | 8.50E-01 | 0.979 | 1587 | 1601 | [0.947; 1.045] |
| Left inferior parietal cortex | 0.995 | -0.5 | 8.64E-01 | 0.979 | 1648 | 1648 | [0.948; 1.045] |
| Left lateral occipital cortex | 0.995 | -0.5 | 8.52E-01 | 0.979 | 1647 | 1647 | [0.948; 1.044] |
| Left transverse temporal gyrus | 0.993 | -0.7 | 7.84E-01 | 0.969 | 1647 | 1645 | [0.946; 1.042] |
| Right pars opercularis | 0.993 | -0.7 | 7.98E-01 | 0.969 | 1646 | 1648 | [0.946; 1.043] |
| Right superior parietal cortex | 0.991 | -0.9 | 7.29E-01 | 0.936 | 1649 | 1648 | [0.944; 1.040] |
| Left pericalcarine cortex | 0.99 | -1 | 7.04E-01 | 0.936 | 1642 | 1644 | [0.943; 1.040] |
| Right pericalcarine cortex | 0.99 | -1 | 6.86E-01 | 0.933 | 1639 | 1641 | [0.942; 1.039] |
| Right entorhinal cortex | 0.984 | -1.6 | 5.29E-01 | 0.834 | 1575 | 1589 | [0.936; 1.034] |
| Right lateral orbitofrontal cortex | 0.979 | -2.1 | 3.98E-01 | 0.731 | 1648 | 1647 | [0.933; 1.028] |
| Right parahippocampal gyrus | 0.979 | -2.1 | 3.97E-01 | 0.731 | 1649 | 1650 | [0.932; 1.028] |
| Right caudal middle frontal gyrus | 0.975 | -2.5 | 3.12E-01 | 0.685 | 1649 | 1647 | [0.929; 1.024] |
| Right pars orbitalis | 0.964 | -3.6 | 1.39E-01 | 0.4 | 1650 | 1651 | [0.918; 1.012] |
| Right temporal pole | 0.928 | -7.2 | 3.00E-03 | 0.088 | 1649 | 1644 | [0.883; 0.974] |
| CVR, coefficient of variation ratio; MDD, major depressive disorder; HC, healthy control; FDR, false discovery rate; CI, confidence interval. | | | | | | | |

**Supplementary Table S15.** ENIGMA – Major Depressive Disorder Working Group ANOVA of clinical characteristics within the MDD group on PBSI scores in CT.

| Analysis of Variance Table | | | | | | |
| --- | --- | --- | --- | --- | --- | --- |
| **Response: PBSI** | | | | | | |
|  | Df | Sum Sq | Mean Sq | F value | Pr(>F) |  |
| AD | 1.00E+00 | 1.37E-02 | 1.37E-02 | 1.35E+01 | 2.39E-04 | *** |
| Recur | 1.00E+00 | 8.36E-04 | 8.36E-04 | 8.22E-01 | 3.64E-01 |  |
| AO | 1.00E+00 | 1.76E-03 | 1.76E-03 | 1.74E+00 | 1.87E-01 |  |
| Age | 1.00E+00 | 6.88E-03 | 6.88E-03 | 6.76E+00 | 9.37E-03 | ** |
| Sex | 1.00E+00 | 4.81E-06 | 4.81E-06 | 4.72E-03 | 9.45E-01 |  |
| Residuals | 2.55E+03 | 2.59E+00 | 1.01E-03 |  |  |  |
| Signif. codes: 0.0001 ‘***’ 0.001 ‘**’ 0.01 ‘*’ 0.05 ‘.’ 0.1 ‘ ’ 1 | | | | | | |
| PBSI, person-based similarity index; Df, degrees of freedom; Sq, square; AD, antidepressant; Recur, recurrence; AO, age of illness onset. | | | | | | |

**Supplementary Table S16.** ENIGMA – Major Depressive Disorder Working Group PBSI score differences between MDD patients using ADs, MDD patients not using ADs and age-/sex-matched HCs in CT.

| Metric | Contrast | Cohen’s d  (95% CI) | Mean (SD)  AD use | Mean (SD)  no AD use | Mean (SD)  HC | estimate | SE | df | t.ratio | FDR |
| --- | --- | --- | --- | --- | --- | --- | --- | --- | --- | --- |
| CT | no AD use - HC | -0.097  [-0.165, -0.029] |  | 0.806  (3.16E-02) | 0.809  (2.85E-02) | 2.50E-03 | 1.06E-03 | 5.17E+03 | 2.35E+00 | 5.60E-02 |
|  | AD use - HC | -0.386  [-0.448, -0.324] | 0.797  (3.19E-02) |  | 0.809  (2.85E-02) | 1.17E-02 | 9.97E-04 | 5.17E+03 | 1.18E+01 | 5.13E-31 |
|  | AD use - no AD use | -0.272  [-0.341, -0.203] | 0.797  (3.19E-02) | 0.806  (3.16E-02) |  | 9.22E-03 | 1.19E-03 | 5.17E+03 | 7.72E+00 | 4.13E-14 |
| SD, standard deviation; AD, antidepressant; HC, healthy control; SE, standard error; df, degrees of freedom; FDR, false discovery rate; CT, cortical thickness. | | | | | | | | | | |

**Supplementary Table S17.** ENIGMA – Major Depressive Disorder Working Group CVR differences between MDD patients using ADs and age-/sex-matched HCs in CT.

| Region | CVR  (AD-use vs HC) | Higher variation  in AD-use (%) | P-value | FDR | N  AD-user | N  HC | 95% CI |
| --- | --- | --- | --- | --- | --- | --- | --- |
| Left precentral gyrus | 1.119 | 11.9 | 3.19E-05 | 0.002 | 1397 | 1396 | [1.062; 1.180] |
| Right precentral gyrus | 1.095 | 9.5 | 7.98E-04 | 0.016 | 1401 | 1401 | [1.038; 1.154] |
| Right fusiform gyrus | 1.094 | 9.4 | 8.14E-04 | 0.016 | 1400 | 1404 | [1.038; 1.153] |
| Right transverse temporal gyrus | 1.094 | 9.4 | 9.32E-04 | 0.016 | 1403 | 1395 | [1.038; 1.153] |
| Left insula | 1.089 | 8.9 | 1.60E-03 | 0.022 | 1391 | 1402 | [1.033; 1.148] |
| Left fusiform gyrus | 1.078 | 7.8 | 5.07E-03 | 0.043 | 1402 | 1403 | [1.023; 1.136] |
| Left posterior cingulate cortex | 1.078 | 7.8 | 5.11E-03 | 0.043 | 1403 | 1403 | [1.023; 1.136] |
| Right insula | 1.078 | 7.8 | 5.11E-03 | 0.043 | 1386 | 1399 | [1.023; 1.137] |
| Left precuneus | 1.068 | 6.8 | 1.36E-02 | 0.102 | 1401 | 1403 | [1.014; 1.126] |
| Right inferior temporal gyrus | 1.067 | 6.7 | 1.54E-02 | 0.105 | 1388 | 1402 | [1.012; 1.125] |
| Left paracentral lobule | 1.064 | 6.4 | 2.04E-02 | 0.126 | 1404 | 1402 | [1.010; 1.122] |
| Right paracentral lobule | 1.061 | 6.1 | 2.73E-02 | 0.155 | 1405 | 1401 | [1.007; 1.118] |
| Right cuneus | 1.058 | 5.8 | 3.49E-02 | 0.182 | 1393 | 1402 | [1.004; 1.116] |
| Right precuneus | 1.057 | 5.7 | 3.81E-02 | 0.185 | 1403 | 1402 | [1.003; 1.114] |
| Left superior temporal gyrus | 1.055 | 5.5 | 4.92E-02 | 0.209 | 1351 | 1373 | [1.000; 1.113] |
| Right rostral anterior cingulate cortex | 1.054 | 5.4 | 4.81E-02 | 0.209 | 1403 | 1403 | [1.000; 1.112] |
| Left medial orbitofrontal cortex | 1.053 | 5.3 | 5.39E-02 | 0.216 | 1398 | 1391 | [0.999; 1.110] |
| Left caudal anterior cingulate cortex | 1.05 | 5 | 6.76E-02 | 0.239 | 1405 | 1395 | [0.996; 1.107] |
| Left pars opercularis | 1.05 | 5 | 6.70E-02 | 0.239 | 1402 | 1403 | [0.997; 1.107] |
| Left rostral anterior cingulate cortex | 1.05 | 5 | 7.03E-02 | 0.239 | 1399 | 1400 | [0.996; 1.107] |
| Left isthmus cingulate cortex | 1.046 | 4.6 | 9.39E-02 | 0.278 | 1399 | 1402 | [0.992; 1.103] |
| Right isthmus cingulate cortex | 1.046 | 4.6 | 9.13E-02 | 0.278 | 1402 | 1404 | [0.993; 1.103] |
| Right superior temporal gyrus | 1.046 | 4.6 | 9.35E-02 | 0.278 | 1388 | 1398 | [0.992; 1.103] |
| Left superior frontal gyrus | 1.045 | 4.5 | 1.01E-01 | 0.285 | 1403 | 1403 | [0.991; 1.101] |
| Right posterior cingulate cortex | 1.043 | 4.3 | 1.20E-01 | 0.308 | 1404 | 1403 | [0.989; 1.099] |
| Right superior frontal gyrus | 1.043 | 4.3 | 1.20E-01 | 0.308 | 1402 | 1403 | [0.989; 1.099] |
| Left superior parietal cortex | 1.042 | 4.2 | 1.27E-01 | 0.308 | 1404 | 1395 | [0.988; 1.098] |
| Left supramarginal cortex | 1.042 | 4.2 | 1.29E-01 | 0.308 | 1387 | 1392 | [0.988; 1.098] |
| Right banks superior temporal sulcus | 1.041 | 4.1 | 1.36E-01 | 0.308 | 1390 | 1396 | [0.987; 1.097] |
| Right inferior parietal cortex | 1.041 | 4.1 | 1.35E-01 | 0.308 | 1398 | 1399 | [0.988; 1.097] |
| Left temporal pole | 1.04 | 4 | 1.47E-01 | 0.323 | 1403 | 1397 | [0.986; 1.096] |
| Left postcentral gyrus | 1.039 | 3.9 | 1.52E-01 | 0.323 | 1399 | 1386 | [0.986; 1.096] |
| Left lingual gyrus | 1.038 | 3.8 | 1.60E-01 | 0.325 | 1403 | 1400 | [0.985; 1.095] |
| Right postcentral gyrus | 1.037 | 3.7 | 1.83E-01 | 0.356 | 1401 | 1394 | [0.983; 1.093] |
| Left caudal middle frontal gyrus | 1.031 | 3.1 | 2.65E-01 | 0.500 | 1401 | 1397 | [0.978; 1.086] |
| Left transverse temporal gyrus | 1.029 | 2.9 | 2.99E-01 | 0.536 | 1404 | 1399 | [0.976; 1.085] |
| Left cuneus | 1.028 | 2.8 | 3.13E-01 | 0.536 | 1399 | 1402 | [0.975; 1.083] |
| Right superior parietal cortex | 1.028 | 2.8 | 3.12E-01 | 0.536 | 1404 | 1401 | [0.975; 1.083] |
| Left lateral orbitofrontal cortex | 1.025 | 2.5 | 3.55E-01 | 0.589 | 1401 | 1396 | [0.973; 1.081] |
| Left banks superior temporal sulcus | 1.024 | 2.4 | 3.89E-01 | 0.610 | 1313 | 1376 | [0.971; 1.081] |
| Left inferior temporal gyrus | 1.024 | 2.4 | 3.81E-01 | 0.610 | 1375 | 1398 | [0.972; 1.080] |
| Right supramarginal gyrus | 1.023 | 2.3 | 3.94E-01 | 0.610 | 1390 | 1404 | [0.971; 1.079] |
| Right caudal anterior cingulate cortex | 1.022 | 2.2 | 4.30E-01 | 0.639 | 1404 | 1404 | [0.969; 1.077] |
| Right middle temporal gyrus | 1.021 | 2.1 | 4.58E-01 | 0.651 | 1399 | 1401 | [0.968; 1.076] |
| Right lateral occipital cortex | 1.02 | 2 | 4.65E-01 | 0.651 | 1400 | 1403 | [0.968; 1.075] |
| Right parahippocampal gyrus | 1.02 | 2 | 4.69E-01 | 0.651 | 1404 | 1403 | [0.968; 1.075] |
| Right frontal pole | 1.012 | 1.2 | 6.69E-01 | 0.868 | 1404 | 1403 | [0.960; 1.067] |
| Left frontal pole | 1.011 | 1.1 | 7.12E-01 | 0.871 | 1405 | 1403 | [0.958; 1.065] |
| Left middle temporal gyrus | 1.011 | 1.1 | 6.88E-01 | 0.868 | 1337 | 1386 | [0.959; 1.067] |
| Left rostral middle frontal gyrus | 1.011 | 1.1 | 6.89E-01 | 0.868 | 1404 | 1402 | [0.960; 1.066] |
| Left lateral occipital cortex | 1.01 | 1 | 7.25E-01 | 0.871 | 1399 | 1403 | [0.958; 1.064] |
| Right lingual gyrus | 1.009 | 0.9 | 7.43E-01 | 0.871 | 1400 | 1401 | [0.958; 1.064] |
| Right pars opercularis | 1.009 | 0.9 | 7.56E-01 | 0.871 | 1402 | 1404 | [0.957; 1.063] |
| Right rostral middle frontal gyrus | 1.009 | 0.9 | 7.43E-01 | 0.871 | 1402 | 1400 | [0.958; 1.064] |
| Left inferior parietal cortex | 1.007 | 0.7 | 7.96E-01 | 0.893 | 1401 | 1399 | [0.956; 1.062] |
| Left parahippocampal gyrus | 1.007 | 0.7 | 8.01E-01 | 0.893 | 1403 | 1404 | [0.955; 1.062] |
| Right pericalcarine cortex | 1.003 | 0.3 | 9.09E-01 | 0.942 | 1388 | 1397 | [0.952; 1.058] |
| Left entorhinal cortex | 1 | 0 | 9.89E-01 | 0.990 | 1352 | 1386 | [0.947; 1.055] |
| Right temporal pole | 1 | 0 | 9.90E-01 | 0.990 | 1404 | 1400 | [0.948; 1.054] |
| Left pars triangularis | 0.997 | -0.3 | 9.14E-01 | 0.942 | 1404 | 1402 | [0.946; 1.051] |
| Right entorhinal cortex | 0.996 | -0.4 | 8.82E-01 | 0.937 | 1331 | 1371 | [0.943; 1.051] |
| Right lateral orbitofrontal cortex | 0.995 | -0.5 | 8.71E-01 | 0.937 | 1402 | 1399 | [0.944; 1.049] |
| Left pericalcarine cortex | 0.993 | -0.7 | 8.20E-01 | 0.899 | 1398 | 1398 | [0.942; 1.047] |
| Left pars orbitalis | 0.986 | -1.4 | 6.02E-01 | 0.802 | 1403 | 1403 | [0.935; 1.039] |
| Right medial orbitofrontal cortex | 0.983 | -1.7 | 5.40E-01 | 0.735 | 1402 | 1399 | [0.933; 1.036] |
| Right caudal middle frontal gyrus | 0.979 | -2.1 | 4.32E-01 | 0.639 | 1402 | 1400 | [0.929; 1.032] |
| Right pars orbitalis | 0.973 | -2.7 | 3.15E-01 | 0.536 | 1404 | 1405 | [0.923; 1.026] |
| Right pars triangularis | 0.963 | -3.7 | 1.63E-01 | 0.325 | 1404 | 1401 | [0.914; 1.015] |
| CVR, coefficient of variation ratio; AD, antidepressant; HC, healthy control; FDR, false discovery rate; CI, confidence interval. | | | | | | | |

**Supplementary Table S18.** ENIGMA – Major Depressive Disorder Working Group CVR differences between MDD patients not using ADs and age-/sex-matched HCs in CT.

| Region | CVR  (no AD-use vs HC) | Higher variation in no AD-use (%) | P-value | FDR | N  no AD-use | N  HC | 95% CI |
| --- | --- | --- | --- | --- | --- | --- | --- |
| Right transverse temporal gyrus | 1.06 | 6 | 5.00E-02 | 0.989 | 1163 | 1159 | [1.000; 1.123] |
| Right isthmus cingulate cortex | 1.056 | 5.6 | 6.30E-02 | 0.989 | 1167 | 1168 | [0.997; 1.119] |
| Left inferior temporal gyrus | 1.049 | 4.9 | 1.07E-01 | 0.989 | 1160 | 1163 | [0.990; 1.111] |
| Right precentral gyrus | 1.048 | 4.8 | 1.12E-01 | 0.989 | 1164 | 1165 | [0.989; 1.110] |
| Left isthmus cingulate cortex | 1.045 | 4.5 | 1.33E-01 | 0.989 | 1165 | 1165 | [0.987; 1.108] |
| Left middle temporal gyrus | 1.045 | 4.5 | 1.39E-01 | 0.989 | 1143 | 1155 | [0.986; 1.107] |
| Left temporal pole | 1.041 | 4.1 | 1.77E-01 | 0.989 | 1165 | 1161 | [0.982; 1.103] |
| Right superior temporal gyrus | 1.041 | 4.1 | 1.77E-01 | 0.989 | 1155 | 1159 | [0.982; 1.103] |
| Right inferior temporal gyrus | 1.036 | 3.6 | 2.28E-01 | 0.989 | 1165 | 1168 | [0.978; 1.098] |
| Left caudal anterior cingulate cortex | 1.035 | 3.5 | 2.47E-01 | 0.989 | 1167 | 1160 | [0.977; 1.097] |
| Left fusiform gyrus | 1.035 | 3.5 | 2.45E-01 | 0.989 | 1165 | 1167 | [0.977; 1.096] |
| Right entorhinal cortex | 1.03 | 3 | 3.23E-01 | 0.989 | 1140 | 1144 | [0.972; 1.093] |
| Left cuneus | 1.026 | 2.6 | 3.94E-01 | 0.989 | 1164 | 1165 | [0.968; 1.087] |
| Left postcentral gyrus | 1.026 | 2.6 | 3.96E-01 | 0.989 | 1162 | 1154 | [0.968; 1.087] |
| Left rostral anterior cingulate cortex | 1.025 | 2.5 | 4.09E-01 | 0.989 | 1163 | 1163 | [0.967; 1.086] |
| Left precentral gyrus | 1.023 | 2.3 | 4.42E-01 | 0.989 | 1163 | 1160 | [0.966; 1.084] |
| Right fusiform gyrus | 1.023 | 2.3 | 4.46E-01 | 0.989 | 1166 | 1167 | [0.966; 1.084] |
| Left posterior cingulate cortex | 1.022 | 2.2 | 4.65E-01 | 0.989 | 1166 | 1163 | [0.965; 1.083] |
| Right cuneus | 1.022 | 2.2 | 4.67E-01 | 0.989 | 1162 | 1165 | [0.965; 1.083] |
| Left caudal middle frontal gyrus | 1.02 | 2 | 5.08E-01 | 0.989 | 1165 | 1163 | [0.963; 1.081] |
| Left entorhinal cortex | 1.019 | 1.9 | 5.34E-01 | 0.989 | 1150 | 1152 | [0.961; 1.081] |
| Left insula | 1.019 | 1.9 | 5.38E-01 | 0.989 | 1164 | 1162 | [0.962; 1.079] |
| Right pars opercularis | 1.019 | 1.9 | 5.38E-01 | 0.989 | 1163 | 1165 | [0.962; 1.079] |
| Right rostral anterior cingulate cortex | 1.019 | 1.9 | 5.39E-01 | 0.989 | 1164 | 1165 | [0.962; 1.079] |
| Left superior temporal gyrus | 1.017 | 1.7 | 5.76E-01 | 0.989 | 1145 | 1137 | [0.960; 1.078] |
| Left pars triangularis | 1.016 | 1.6 | 5.95E-01 | 0.989 | 1165 | 1163 | [0.959; 1.077] |
| Right caudal anterior cingulate cortex | 1.015 | 1.5 | 6.31E-01 | 0.989 | 1165 | 1168 | [0.958; 1.075] |
| Right caudal middle frontal gyrus | 1.015 | 1.5 | 6.22E-01 | 0.989 | 1166 | 1162 | [0.958; 1.075] |
| Right superior frontal gyrus | 1.014 | 1.4 | 6.52E-01 | 0.989 | 1166 | 1168 | [0.957; 1.074] |
| Right parahippocampal gyrus | 1.013 | 1.3 | 6.87E-01 | 0.989 | 1166 | 1167 | [0.956; 1.073] |
| Right medial orbitofrontal cortex | 1.012 | 1.2 | 6.97E-01 | 0.989 | 1162 | 1163 | [0.955; 1.072] |
| Left precuneus | 1.011 | 1.1 | 7.35E-01 | 0.989 | 1161 | 1166 | [0.954; 1.071] |
| Right middle temporal gyrus | 1.011 | 1.1 | 7.11E-01 | 0.989 | 1160 | 1165 | [0.955; 1.072] |
| Right rostral middle frontal gyrus | 1.008 | 0.8 | 7.94E-01 | 0.989 | 1163 | 1163 | [0.952; 1.068] |
| Left superior frontal gyrus | 1.005 | 0.5 | 8.65E-01 | 0.989 | 1166 | 1167 | [0.949; 1.065] |
| Left supramarginal cortex | 1.005 | 0.5 | 8.76E-01 | 0.989 | 1160 | 1157 | [0.948; 1.065] |
| Right supramarginal gyrus | 1.005 | 0.5 | 8.82E-01 | 0.989 | 1164 | 1165 | [0.948; 1.064] |
| Left pars opercularis | 1.004 | 0.4 | 9.09E-01 | 0.989 | 1167 | 1164 | [0.947; 1.063] |
| Right pericalcarine cortex | 1.004 | 0.4 | 8.96E-01 | 0.989 | 1165 | 1162 | [0.948; 1.064] |
| Left frontal pole | 1.002 | 0.2 | 9.58E-01 | 0.989 | 1168 | 1166 | [0.945; 1.062] |
| Right lateral occipital cortex | 1.002 | 0.2 | 9.39E-01 | 0.989 | 1167 | 1164 | [0.946; 1.062] |
| Right pars triangularis | 1.002 | 0.2 | 9.60E-01 | 0.989 | 1165 | 1164 | [0.945; 1.061] |
| Right lingual gyrus | 1.001 | 0.1 | 9.71E-01 | 0.989 | 1163 | 1164 | [0.945; 1.061] |
| Right paracentral lobule | 1.001 | 0.1 | 9.86E-01 | 0.989 | 1167 | 1164 | [0.944; 1.060] |
| Right posterior cingulate cortex | 1 | 0 | 9.89E-01 | 0.989 | 1167 | 1167 | [0.944; 1.059] |
| Right superior parietal cortex | 0.999 | -0.1 | 9.70E-01 | 0.989 | 1167 | 1165 | [0.943; 1.058] |
| Left parahippocampal gyrus | 0.998 | -0.2 | 9.40E-01 | 0.989 | 1163 | 1167 | [0.941; 1.057] |
| Left medial orbitofrontal cortex | 0.996 | -0.4 | 9.06E-01 | 0.989 | 1161 | 1153 | [0.940; 1.056] |
| Left pericalcarine cortex | 0.994 | -0.6 | 8.45E-01 | 0.989 | 1164 | 1163 | [0.938; 1.053] |
| Right postcentral gyrus | 0.992 | -0.8 | 7.98E-01 | 0.989 | 1165 | 1160 | [0.936; 1.051] |
| Left paracentral lobule | 0.991 | -0.9 | 7.60E-01 | 0.989 | 1166 | 1165 | [0.935; 1.049] |
| Left pars orbitalis | 0.991 | -0.9 | 7.77E-01 | 0.989 | 1165 | 1165 | [0.935; 1.050] |
| Left rostral middle frontal gyrus | 0.99 | -1 | 7.36E-01 | 0.989 | 1165 | 1166 | [0.934; 1.048] |
| Left lateral orbitofrontal cortex | 0.989 | -1.1 | 7.32E-01 | 0.989 | 1167 | 1160 | [0.934; 1.048] |
| Right lateral orbitofrontal cortex | 0.988 | -1.2 | 6.90E-01 | 0.989 | 1166 | 1164 | [0.932; 1.046] |
| Left superior parietal cortex | 0.987 | -1.3 | 6.80E-01 | 0.989 | 1165 | 1163 | [0.932; 1.046] |
| Right frontal pole | 0.986 | -1.4 | 6.43E-01 | 0.989 | 1165 | 1166 | [0.930; 1.045] |
| Right inferior parietal cortex | 0.986 | -1.4 | 6.49E-01 | 0.989 | 1167 | 1164 | [0.931; 1.045] |
| Right precuneus | 0.986 | -1.4 | 6.53E-01 | 0.989 | 1166 | 1166 | [0.931; 1.045] |
| Right insula | 0.985 | -1.5 | 6.24E-01 | 0.989 | 1165 | 1159 | [0.930; 1.044] |
| Left lingual gyrus | 0.984 | -1.6 | 5.88E-01 | 0.989 | 1162 | 1163 | [0.928; 1.042] |
| Right banks superior temporal sulcus | 0.983 | -1.7 | 5.70E-01 | 0.989 | 1158 | 1157 | [0.928; 1.041] |
| Left inferior parietal cortex | 0.976 | -2.4 | 4.18E-01 | 0.989 | 1164 | 1165 | [0.921; 1.034] |
| Right temporal pole | 0.974 | -2.6 | 3.71E-01 | 0.989 | 1166 | 1164 | [0.919; 1.032] |
| Left lateral occipital cortex | 0.972 | -2.8 | 3.42E-01 | 0.989 | 1167 | 1165 | [0.918; 1.030] |
| Right pars orbitalis | 0.969 | -3.1 | 2.87E-01 | 0.989 | 1165 | 1168 | [0.914; 1.026] |
| Left transverse temporal gyrus | 0.967 | -3.3 | 2.58E-01 | 0.989 | 1163 | 1165 | [0.912; 1.025] |
| Left banks superior temporal sulcus | 0.966 | -3.4 | 2.53E-01 | 0.989 | 1137 | 1144 | [0.912; 1.024] |
| CVR, coefficient of variation ratio; AD, antidepressant; HC, healthy control; FDR, false discovery rate; CI, confidence interval. | | | | | | | |

**Supplementary Table S19.** ENIGMA – Major Depressive Disorder Working Group Clinical and demographic differences between *infra-norm*, *norm*, and *supra-norm* MDD patients in CT.

| Variable  (cont.) | Contrast | Cohen’s d  (95% CI) | Mean (SD)  Infra Norm | Mean (SD)  Norm | Mean (SD)  Supra Norm | N  Infra Norm | N  Norm | N  Supra Norm | W.statistic | P-Value |
| --- | --- | --- | --- | --- | --- | --- | --- | --- | --- | --- |
| Age | Infra Norm - Norm | 0.056  [-0.100, 0.212] | 39.74  (1.54E+01) | 38.91  (1.49E+01) |  | 168 | 1875 |  | 1.63E+05 | 2.15E-01 |
|  | Infra Norm - Supra Norm | -0.034  [-0.602, 0.534] | 39.74  (1.54E+01) |  | 40.27 (1.72E+01) | 168 |  | 11 | 9.15E+02 | 5.23E-01 |
|  | Norm - Supra Norm | -0.092  [-0.662, 0.478] |  | 38.91  (1.49E+01) | 40.27 (1.72E+01) |  | 1875 | 11 | 9.86E+03 | 5.99E-01 |
| AO | Infra Norm - Norm | 0.003  [-0.153, 0.159] | 29.13  (1.49E+01) | 29.09  (1.40E+01) |  | 168 | 1875 |  | 1.57E+05 | 5.38E-01 |
|  | Infra Norm - Supra Norm | 0.015  [-0.553, 0.583] | 29.13  1.49E+01) |  | 28.91 (1.40E+01) | 168 |  | 11 | 9.44E+02 | 4.55E-01 |
|  | Norm - Supra Norm | 0.013  [-0.557, 0.583] |  | 29.09  (1.40E+01) | 28.91 (1.40E+01) |  | 1875 | 11 | 1.04E+04 | 4.79E-01 |
| Sev | Infra Norm - Norm | 0.252  [-0.033, 0.537] | 4.15  (3.00E+00) | 3.47  (2.66E+00) |  | 59 | 548 |  | 1.81E+04 | 6.54E-02 |
|  | Infra Norm - Supra Norm | 0.465  [-0.502, 1.432] | 4.15  (3.00E+00) |  | 2.75 (3.20E+00) | 59 |  | 4 | 1.56E+02 | 1.47E-01 |
|  | Norm - Supra Norm | 0.272  [-0.699, 1.243] |  | 3.47  (2.66E+00) | 2.75 (3.20E+00) |  | 548 | 4 | 1.29E+03 | 2.73E-01 |
| BDI | Infra Norm - Norm | 0.368  [0.110, 0.626] | 26.91  (1.24E+01) | 22.57  (1.18E+01) |  | 68 | 811 |  | 3.32E+04 | 2.76E-03 |
|  | Infra Norm - Supra Norm | -0.068  [-1.036, 0.900] | 26.91  (1.24E+01) |  | 27.75 (8.66E+00) | 68 |  | 4 | 1.31E+02 | 5.59E-01 |
|  | Norm - Supra Norm | -0.441  [-1.415, 0.533] |  | 22.57  (1.18E+01) | 27.75 (8.66E+00) |  | 811 | 4 | 1.18E+03 | 8.25E-01 |
| HDRS | Infra Norm - Norm | 0.196  [0.018, 0.374] | 15.49  (8.35E+00) | 13.90  (8.11E+00) |  | 129 | 1458 |  | 1.04E+05 | 2.83E-02 |
|  | Infra Norm - Supra Norm | 0.228  [-0.700, 1.156] | 15.49  (8.35E+00) |  | 13.60 (5.41E+00) | 129 |  | 5 | 3.60E+02 | 3.32E-01 |
|  | Norm - Supra Norm | 0.037  [-0.883, 0.957] |  | 13.90  (8.11E+00) | 13.60 (5.41E+00) |  | 1458 | 5 | 3.74E+03 | 4.61E-01 |

| Epi | Infra Norm - Norm | -0.043  [-0.199, 0.113] | 3.55  (4.30E+00) | 3.79  (5.65E+00) |  |  | 1751 |  | 1.34E+05 | 4.39E-01 |
| --- | --- | --- | --- | --- | --- | --- | --- | --- | --- | --- |
|  | Infra Norm - Supra Norm | -1.006  [-2.081, 0.069] | 3.55  (4.30E+00) |  | 8.60 (1.18E+01) | 152 |  | 10 | 6.66E+02 | 7.51E-01 |
|  | Norm - Supra Norm | -0.845  [-1.899, 0.209] |  | 3.79  (5.65E+00) | 8.60 (1.18E+01) | 152 | 1751 | 10 | 7.67E+03 | 7.57E-01 |
| Cont, continuous, SD, standard deviation; AO, age at illness onset; Sev, severity (number of diagnostic criteria for major depressive disorder meet according to Diagnostic and Statistic Manual 4th edition); BDI, Becks Depression Inventory 1st and 2nd edition; HDRS, Hamilton Depression Rating Scale 17 items; Epi, number of episodes. | | | | | | | | | | |

*(continued on next page)*

**Supplementary Table S19.** *(continued)*

| Variable  (cat.) | Contrast | χ² | P-Value | N  Infra Norm | | N  Norm | | N  Supra Norm | |
| --- | --- | --- | --- | --- | --- | --- | --- | --- | --- |
| Sex | Infra Norm - Norm | 5.14E+00 | 2.34E-02 | Male | Female | Male | Female | Male | Female |
|  | Infra Norm - Supra Norm | 7.18E-01 | 3.97E-01 | 61 | 150 | 854 | 1477 | 6 | 8 |
|  | Norm - Supra Norm | 4.69E-02 | 8.28E-01 |  |  |  |  |  |  |
| Recur | Infra Norm - Norm | 4.20E-06 | 9.98E-01 | Recurrent | First Epi. | Recurrent | First Epi. | Recurrent | First Epi. |
|  | Infra Norm - Supra Norm | 2.29E-03 | 9.62E-01 | 125 | 86 | 1404 | 927 | 6 | 8 |
|  | Norm - Supra Norm | 2.04E-03 | 9.64E-01 |  |  |  |  |  |  |
| AD | Infra Norm - Norm | 4.27E-01 | 5.13E-01 | AD-use | no AD-use | AD-use | no AD-use | AD-use | no AD-use |
|  | Infra Norm - Supra Norm | 3.16E-01 | 5.74E-01 | 111 | 100 | 1281 | 1050 | 5 | 9 |
|  | Norm - Supra Norm | 6.43E-01 | 4.23E-01 |  |  |  |  |  |  |
| Rem | Infra Norm - Norm | 9.95E-01 | 3.19E-01 | Remitted | Depressed | Remitted | Depressed | Remitted | Depressed |
|  | Infra Norm - Supra Norm | 9.95E-01 | 1.00E+00 | 23 | 145 | 319 | 1556 | 1 | 10 |
|  | Norm - Supra Norm | 9.95E-01 | 7.02E-01 |  |  |  |  |  |  |
| Cat, categorical, Recur, recurrence status; AD, antidepressant; Rem, remission status. | | | | | | | | | |

**Supplementary Table S20.** ENIGMA – Major Depressive Disorder Working Group Clinical and demographic differences between *infra-norm*, *norm*, and *supra-norm* MDD patients in CSA.

| Variable  (cont.) | Contrast | Cohen’s d  (95% CI) | Mean (SD)  Infra Norm | Mean (SD)  Norm | Mean (SD)  Supra Norm | N  Infra Norm | N  Norm | N  Supra Norm | W.statistic | P-Value |
| --- | --- | --- | --- | --- | --- | --- | --- | --- | --- | --- |
| Age | Infra Norm - Norm | 0.159  [-0.001, 0.319] | 41.22 (1.47E+01) | 38.85 (1.49E+01) |  | 149 | 1883 |  | 1.55E+05 | 3.88E-02 |
|  | Infra Norm - Supra Norm | 0.181  [-0.231, 0.593] | 41.22 (1.47E+01) |  | 38.50 (1.65E+01) | 149 |  | 32 | 2.62E+03 | 3.79E-01 |
|  | Norm - Supra Norm | 0.024 [−0.374, 0.422] |  | 38.85 (1.49E+01) | 38.50 (1.65E+01) |  | 1883 | 32 | 3.05E+04 | 9.05E-01 |
| AO | Infra Norm - Norm | 0.076  [-0.084, 0.236] | 30.15 (1.37E+01) | 29.07 (1.41E+01) |  | 149 | 1883 |  | 1.48E+05 | 2.47E-01 |
|  | Infra Norm - Supra Norm | 0.073  [-0.339, 0.485] | 30.15 (1.37E+01) |  | 29.13 (1.49E+01) | 149 |  | 32 | 2.54E+03 | 5.76E-01 |
|  | Norm - Supra Norm | -0.004  [-0.402, 0.394] |  | 29.07 (1.41E+01) | 29.13 (1.49E+01) |  | 1883 | 32 | 3.03E+04 | 9.48E-01 |
| Sev | Infra Norm - Norm | 0.020  [-0.340, 0.380] | 3.58 (2.73E+00) | 3.53 (2.69E+00) |  | 36 | 564 |  | 1.02E+04 | 9.23E-01 |
|  | Infra Norm - Supra Norm | 0.089  [-0.619, 0.797] | 3.58 (2.73E+00) |  | 3.33 (3.20E+00) | 36 |  | 9 | 1.76E+02 | 7.10E-01 |
|  | Norm - Supra Norm | 0.072  [-0.637, 0.781] |  | 3.53 (2.69E+00) | 3.33 (3.20E+00) |  | 564 | 9 | 2.71E+03 | 7.28E-01 |
| BDI | Infra Norm - Norm | 0.099  [-0.161, 0.359] | 23.83 (1.23E+01) | 22.65 (1.19E+01) |  | 63 | 816 |  | 2.67E+04 | 6.19E-01 |
|  | Infra Norm - Supra Norm | -0.095  [-0.865, 0.675] | 23.83 (1.23E+01) |  | 25.00 (1.32E+01) | 63 |  | 15 | 4.36E+02 | 6.43E-01 |
|  | Norm - Supra Norm | -0.197  [-0.977, 0.583] |  | 22.65 (1.19E+01) | 25.00 (1.32E+01) |  | 816 | 15 | 5.44E+03 | 4.62E-01 |
| HDRS | Infra Norm - Norm | 0.132  [-0.046, 0.310] | 14.98 (8.36E+00) | 13.91 (8.13E+00) |  | 122 | 1457 |  | 9.53E+04 | 1.87E-01 |
|  | Infra Norm - Supra Norm | 0.344  [-0.147, 0.835] | 14.98 (8.36E+00) |  | 12.13 (8.10E+00) | 122 |  | 24 | 1.75E+03 | 1.29E-01 |
|  | Norm - Supra Norm | 0.219  [-0.274, 0.712] |  | 13.91 (8.13E+00) | 12.13 (8.10E+00) |  | 1457 | 24 | 1.96E+04 | 3.17E-01 |

| Epi | Infra Norm - Norm | -0.032  [-0.192, 0.128] | 3.66 (3.75E+00) | 3.85 (5.80E+00) |  | 139 | 1752 |  | 1.29E+05 | 2.31E-01 |
| --- | --- | --- | --- | --- | --- | --- | --- | --- | --- | --- |
|  | Infra Norm - Supra Norm | 0.492  [0.122, 0.862] | 3.66 (3.75E+00) |  | 1.97 (1.30E+00) | 139 |  | 31 | 2.81E+03 | 6.66E-03 |
|  | Norm - Supra Norm | 0.326  [0.056, 0.596] |  | 3.85 (5.80E+00) | 1.97 (1.30E+00) |  | 1752 | 31 | 3.36E+04 | 1.93E-02 |
| Cont, continuous, SD, standard deviation; AO, age at illness onset; Sev, severity (number of diagnostic criteria for major depressive disorder meet according to Diagnostic and Statistic Manual 4th edition); BDI, Becks Depression Inventory 1st and 2nd edition; HDRS, Hamilton Depression Rating Scale 17 items; Epi, number of episodes. | | | | | | | | | | |

*(continued on next page)*

**Supplementary Table S20.** *(continued)*

| Variable  (cat.) | Contrast | χ² | P-Value | N  Infra Norm | | N  Norm | | N  Supra Norm | |
| --- | --- | --- | --- | --- | --- | --- | --- | --- | --- |
| Sex | Infra Norm - Norm | 6.96E-02 | 7.92E-01 | Male | Female | Male | Female | Male | Female |
|  | Infra Norm - Supra Norm | 5.70E-03 | 9.40E-01 | 65 | 122 | 843 | 1498 | 11 | 23 |
|  | Norm - Supra Norm | 6.82E-02 | 7.94E-01 |  |  |  |  |  |  |
| Recur | Infra Norm - Norm | 1.73E-04 | 9.90E-01 | Recurrent | First Epi. | Recurrent | First Epi. | Recurrent | First Epi. |
|  | Infra Norm - Supra Norm | 1.45E+00 | 2.28E-01 | 112 | 75 | 1410 | 931 | 16 | 18 |
|  | Norm - Supra Norm | 1.91E+00 | 1.67E-01 |  |  |  |  |  |  |
| AD | Infra Norm - Norm | 8.70E-30 | 1.00E+00 | AD-use | no AD-use | AD-use | no AD-use | AD-use | no AD-use |
|  | Infra Norm - Supra Norm | 9.12E-02 | 7.63E-01 | 102 | 85 | 1281 | 1060 | 17 | 17 |
|  | Norm - Supra Norm | 1.41E-01 | 7.07E-01 |  |  |  |  |  |  |
| Rem | Infra Norm - Norm | 1.44E+00 | 4.88E-01 | Remitted | Depressed | Remitted | Depressed | Remitted | Depressed |
|  | Infra Norm - Supra Norm | 1.44E+00 | 2.21E-01 | 21 | 128 | 324 | 1559 | 5 | 27 |
|  | Norm - Supra Norm | 1.44E+00 | 2.98E-01 |  |  |  |  |  |  |
| Cat, categorical, Recur, recurrence status; AD, antidepressant; Rem, remission status. | | | | | | | | | |

**Supplementary Table S21.** ENIGMA – Major Depressive Disorder Working Group Clinical and demographic differences between *infra-norm*, *norm*, and *supra-norm* MDD patients in SV.

| Variable  (cont.) | Contrast | Cohen’s d  (95% CI) | Mean (SD)  Infra Norm | Mean (SD)  Norm | Mean (SD)  Supra Norm | N  Infra Norm | N  Norm | N  Supra Norm | W.statistic | P-Value |
| --- | --- | --- | --- | --- | --- | --- | --- | --- | --- | --- |
| Age | Infra Norm - Norm | 0.129  [-0.027, 0.285] | 40.82 (1.50E+01) | 38.89 (1.49E+01) |  | 172 | 1881 |  | 1.74E+05 | 8.90E-02 |
|  | Infra Norm - Supra Norm | NA | 40.82 (1.50E+01) |  | 15.00  (NA) | 172 |  | 1 | NA | NA |
|  | Norm - Supra Norm | NA |  | 38.89 (1.49E+01) | 15.00  (NA) |  | 1881 | 1 | NA | NA |
| AO | Infra Norm - Norm | 0.204  [0.034, 0.374] | 31.83 (1.50E+01) | 28.94 (1.40E+01) |  | 172 | 1881 |  | 1.79E+05 | 2.25E-02 |
|  | Infra Norm - Supra Norm | NA | 31.83 (1.50E+01) |  | 15.00  (NA) | 172 |  | 1 | NA | NA |
|  | Norm - Supra Norm | NA |  | 28.94 (1.40E+01) | 15.00  (NA) |  | 1881 | 1 | NA | NA |
| Sev | Infra Norm - Norm | -0.086  [-0.414, 0.242] | 3.31 (2.84E+00) | 3.55 (2.68E+00) |  | 48 | 561 |  | 1.26E+04 | 4.59E-01 |
|  | Infra Norm - Supra Norm | NA | 3.31 (2.84E+00) |  | 2.00  (NA) | 48 |  | 1 | NA | NA |
|  | Norm - Supra Norm | NA |  | 3.55 (2.68E+00) | 2.00  (NA) |  | 561 | 1 | NA | NA |
| BDI | Infra Norm - Norm | -0.201  [-0.461, 0.059] | 20.56 (1.20E+01) | 22.95 (1.19E+01) |  | 63 | 824 |  | 2.27E+04 | 9.74E-02 |
|  | Infra Norm - Supra Norm | NA | 20.56 (1.20E+01) |  | NA | 63 |  | 0 | NA | NA |
|  | Norm - Supra Norm | NA |  | 22.95 (1.19E+01) | NA |  | 824 | 0 | NA | NA |
| HDRS | Infra Norm - Norm | -0.065  [-0.243, 0.113] | 13.46 (8.33E+00) | 13.99 (8.08E+00) |  | 134 | 1462 |  | 9.41E+04 | 4.56E-01 |
|  | Infra Norm - Supra Norm | NA | 13.46 (8.33E+00) |  | NA | 134 |  | 0 | NA | NA |
|  | Norm - Supra Norm | NA |  | 13.99 (8.08E+00) | NA |  | 1462 | 0 | NA | NA |

| Epi | Infra Norm - Norm | 0.102  [-0.066, 0.270] | 4.33 (7.03E+00) | 3.76 (5.51E+00) |  | 163 | 1747 |  | 1.43E+05 | 9.11E-01 |
| --- | --- | --- | --- | --- | --- | --- | --- | --- | --- | --- |
|  | Infra Norm - Supra Norm | NA | 4.33 (7.03E+00) |  | 1.00 (NA) | 163 |  | 1 | NA | NA |
|  | Norm - Supra Norm | NA |  | 3.76 (5.51E+00) | 1.00 (NA) |  | 1747 | 1 | NA | NA |
| Cont, continuous, SD, standard deviation; AO, age at illness onset; Sev, severity (number of diagnostic criteria for major depressive disorder meet according to Diagnostic and Statistic Manual 4th edition); BDI, Becks Depression Inventory 1st and 2nd edition; HDRS, Hamilton Depression Rating Scale 17 items; Epi, number of episodes. | | | | | | | | | | |

*(continued on next page)*

**Supplementary Table S21.** *(continued)*

| Variable  (cat.) | Contrast | χ² | P-Value | N  Infra Norm | | N  Norm | | N  Supra Norm | |
| --- | --- | --- | --- | --- | --- | --- | --- | --- | --- |
| Sex | Infra Norm - Norm | 8.88E-01 | 3.46E-01 | Male | Female | Male | Female | Male | Female |
|  | Infra Norm - Supra Norm | 8.88E-01 | 1.00E+00 | 75 | 154 | 838 | 1482 | 0 | 1 |
|  | Norm - Supra Norm | 8.88E-01 | 1.00E+00 |  |  |  |  |  |  |
| Recur | Infra Norm - Norm | 3.27E-01 | 5.67E-01 | Recurrent | First Epi. | Recurrent | First Epi. | Recurrent | First Epi. |
|  | Infra Norm - Supra Norm | 3.27E-01 | 4.22E-01 | 133 | 96 | 1398 | 922 | 0 | 1 |
|  | Norm - Supra Norm | 3.27E-01 | 3.98E-01 |  |  |  |  |  |  |
| AD | Infra Norm - Norm | 7.82E-01 | 3.77E-01 | AD-use | no AD-use | AD-use | no AD-use | AD-use | no AD-use |
|  | Infra Norm - Supra Norm | 7.82E-01 | 4.26E-01 | 132 | 97 | 1261 | 1059 | 1 | 0 |
|  | Norm - Supra Norm | 7.82E-01 | 4.57E-01 |  |  |  |  |  |  |
| Rem | Infra Norm - Norm | 5.28E+00 | 7.13E-02 | Remitted | Depressed | Remitted | Depressed | Remitted | Depressed |
|  | Infra Norm - Supra Norm | 5.28E+00 | 1.00E+00 | 31 | 141 | 318 | 1563 | 0 | 1 |
|  | Norm - Supra Norm | 5.28E+00 | 1.00E+00 |  |  |  |  |  |  |
| Cat, categorical, Recur, recurrence status; AD, antidepressant; Rem, remission status. | | | | | | | | | |
