# Supplementary material for: Decomposing neuroanatomical heterogeneity in depression: insights from an ENIGMA major depressive disorder working group study in 5146 individuals
Source: Transl Psychiatry. 2026 Jun 23;16:452. doi: 10.1038/s41398-026-04189-x (PMC13547417; doi:10.1038/s41398-026-04189-x)
Supplement: Supplementary file 3 [file 41398_2026_4189_MOESM3_ESM.docx]

**Decomposing neuroanatomical heterogeneity in depression – Supplementary Figures**

Content:

Validation of harmonization

**Supplementary Figure S1.** Multidimensional scaling plots of site-related structure before and after neuroComBat harmonization in SV, CSA, and CT.

Global and regional neuroanatomical heterogeneity results

**Supplementary Figure S2.** Global heterogeneity differences between MDDs and age-/sex-matched HCs in CT, CSA and SV.

**Supplementary Figure S3.** Regional heterogeneity differences between MDD patients and age-/sex-matched HCs in CSA.

**Supplementary Figure S4.** Regional heterogeneity differences between MDD patients and age-/sex-matched HCs in SV.

Moderator analyses on global and regional neuroanatomical heterogeneity results

**Supplementary Figure S5.** Regional heterogeneity differences between adult MDD patients and sex-matched adolescent MDD patients in CT.

**Supplementary Figure S6.** Regional heterogeneity differences between male MDD patients and age-matched female MDD patients in CT.

**Supplementary Figure S7.** Regional heterogeneity differences between MDD patients using ADs and age-/sex-matched MDD patients not using ADs in CT.

**Supplementary Figure S8.** Regional heterogeneity differences between recurrent MDD patients and age-/sex-matched HCs in CT.

**Supplementary Figure S9.** Regional heterogeneity differences between first episode MDD patients and age-/sex-matched HCs in CT.

**Supplementary Figure S10.** Regional heterogeneity differences between adult-onset MDD patients and age-/sex-matched HCs in CT.

**Supplementary Figure S11.** Regional heterogeneity differences between adolescent-onset MDD patients and age-/sex-matched HCs in CT.

Patient-specific neuroanatomical variability results

**Supplementary Figure S12.** Classification of MDD patients into “infra-norm”, “norm”, “supra-norm” subgroups in CT, CSA, SV.

**Supplementary Figure S1.** Multidimensional scaling plots of site-related structure before and after neuroComBat harmonization in SV, CSA, and CT.

Multidimensional scaling (MDS) plots of each neuroanatomical metric before and after neuroComBat harmonization, illustrating clustering by scanning site in the raw and harmonized data. Axes represent the first two MDS dimensions (MDS1 and MDS2). For visualization purposes, the number of participants was capped at 150 per site.

CT, cortical thickness; CSA, cortical surface area; MDS, multidimensional scaling; SV, subcortical volume.

**Supplementary Figure S2.** Global heterogeneity differences between MDDs and age-/sex-matched HCs in CT, CSA and SV.

(a) Boxplot of global CSA heterogeneity (PBSI scores) in MDD patients and age- and sex-matched HCs (median, IQR) with axis fit to scale.

(b) Boxplot of global SV heterogeneity (PBSI scores) in MDD patients and age- and sex-matched HCs (median, IQR) with axis fit to scale.

(c) Boxplot of global heterogeneity across all neuroanatomical measures in MDD patients and age- and sex-matched HCs (median, IQR) with fixed axis; ****FDR < .0001.

CT, cortical thickness; FDR, false discovery rate; HC, healthy control; IQR, interquartile range; MDD, major depressive disorder; PBSI, person-based similarity index.

**Supplementary Figure S3.** Regional heterogeneity differences between MDD patients and age-/sex-matched HCs in CSA.

Forest plot of CVR for CSA across neuroanatomical regions, where CVR > 1 indicates greater heterogeneity in MDD patients.

CVR, coefficient of variation ratio; CSA, cortical surface area; FDR, false discovery rate; HC, healthy control; LH, left hemisphere; MDD, major depressive disorder; RH, right hemisphere.

**Supplementary Figure S4.** Regional heterogeneity differences between MDD patients and age-/sex-matched HCs in SV.

Forest plot of CVR for SV across neuroanatomical regions, where CVR > 1 indicates greater heterogeneity in MDD patients.

CVR, coefficient of variation ratio; SV, subcortical volume; FDR, false discovery rate; HC, healthy control; LH, left hemisphere; MDD, major depressive disorder; RH, right hemisphere.

**Supplementary Figure S5.** Regional heterogeneity differences between adult MDD patients and sex-matched adolescent MDD patients in CT.

Forest plot of CVR for CT across neuroanatomical regions, where CVR > 1 indicates greater heterogeneity in adult MDD patients.

CVR, coefficient of variation ratio; CT, cortical thickness; FDR, false discovery rate; LH, left hemisphere; MDD, major depressive disorder; RH, right hemisphere.

**Supplementary Figure S6.** Regional heterogeneity differences between male MDD patients and age-matched female MDD patients in CT.

Forest plot of CVR for CT across neuroanatomical regions, where CVR > 1 indicates greater heterogeneity in male MDD patients.

CVR, coefficient of variation ratio; CT, cortical thickness; FDR, false discovery rate; LH, left hemisphere; MDD, major depressive disorder; RH, right hemisphere.

**Supplementary Figure S7.** Regional heterogeneity differences between MDD patients using ADs and age-/sex-matched MDD patients not using ADs in CT.

Forest plot of CVR for CT across neuroanatomical regions, where CVR > 1 indicates greater heterogeneity in male MDD patients.

AD, antidepressant; CVR, coefficient of variation ratio; CT, cortical thickness; FDR, false discovery rate; LH, left hemisphere; MDD, major depressive disorder; RH, right hemisphere.

**Supplementary Figure S8.** Regional heterogeneity differences between recurrent MDD patients and age-/sex-matched HCs in CT.

Forest plot of CVR for CT across neuroanatomical regions, where CVR > 1 indicates greater heterogeneity in MDD patients.

CVR, coefficient of variation ratio; CT, cortical thickness; FDR, false discovery rate; HC, healthy control; LH, left hemisphere; MDD, major depressive disorder; RH, right hemisphere.

**Supplementary Figure S9.** Regional heterogeneity differences between first episode MDD patients and age-/sex-matched HCs in CT.

Forest plot of CVR for CT across neuroanatomical regions, where CVR > 1 indicates greater heterogeneity in MDD patients.

CVR, coefficient of variation ratio; CT, cortical thickness; FDR, false discovery rate; HC, healthy control; LH, left hemisphere; MDD, major depressive disorder; RH, right hemisphere.

**Supplementary Figure S10.** Regional heterogeneity differences between adult-onset MDD patients and age-/sex-matched HCs in CT.

Forest plot of CVR for CT across neuroanatomical regions, where CVR > 1 indicates greater heterogeneity in MDD patients.

CVR, coefficient of variation ratio; CT, cortical thickness; FDR, false discovery rate; HC, healthy control; LH, left hemisphere; MDD, major depressive disorder; RH, right hemisphere.

**Supplementary Figure S11.** Regional heterogeneity differences between adolescent-onset MDD patients and age-/sex-matched HCs in CT.

Forest plot of CVR for CT across neuroanatomical regions, where CVR > 1 indicates greater heterogeneity in MDD patients.

CVR, coefficient of variation ratio; CT, cortical thickness; FDR, false discovery rate; HC, healthy control; LH, left hemisphere; MDD, major depressive disorder; RH, right hemisphere.

**Supplementary Figure S12.** Classification of MDD patients into *infra-norm*, *norm*, *supra-norm* subgroups in CT, CSA, SV.

Distributions of the norm-PBSI values for each MDD patients in all neuroanatomical metrics. The vertical dashed lines indicate percentile-based classification thresholds: *infra-norm* (<5th percentile), *norm* (5th–95th percentile), and *supra-norm* (>95th percentile). The orange boxplots depict the median (IQR).

CT, cortical thickness; IQR, interquartile range; MDD, major depressive disorder; PBSI, person-based similarity index.
